# Convergent Total Synthesis of (−)-Methyl Gummiferolate Enabled by Pattern Recognition and Radical Cyclization

**DOI:** 10.1021/jacs.6c15246

**Published:** 2026-09-28

**Authors:** Weizhao Zhao, Jingsong Zhen, Mingji Dai

**Affiliations:** † Department of Chemistry, 1371 Emory University, Atlanta, Georgia 30322, United States; ‡ Department of Pharmacology and Chemical Biology, School of Medicine, Emory University, Atlanta, Georgia 30322, United States

## Abstract

We report herein an enantioselective total synthesis of (−)-methyl gummiferolate, a complex diterpene natural product with a tetracyclic *ent*-atisane core including a bicyclo[2.2.2]­octane subunit and three all-carbon quaternary centers. Gummiferolic acid has demonstrated potent plant growth-regulatory activity similar or superior to gibberellic acid. Using pattern recognition analysis, the tetracyclic *ent*-atisane core of (−)-methyl gummiferolate was retrosynthetically traced back to chiral pool molecule (*S*)-perillaldehyde and a six-membered ring containing starting material which can be accessed using an enantioselective Diels–Alder reaction with the Rawal diene. Our forward synthesis features a substrate-controlled stereoselective MHAT-initiated Baran reductive olefin cross coupling (BROC), a powerful radical cyclization approach, to close the six-membered B ring and an intramolecular alkylation to build the bicyclo[2.2.2]­octane subunit. The BROC precursor was assembled in a convergent manner using a 1,2-addition of an alkyl lithium to a Weinreb amide derived from (*S*)-perillaldehyde. The alkyl lithium precursor was prepared from a known cyclohexenone (**12**) using key steps including CBS reduction, 2,3-Wittig–Still rearrangement, and Appel iodination. Overall, (−)-methyl gummiferolate was prepared in 18 steps from **12**.

## Graphical abstract

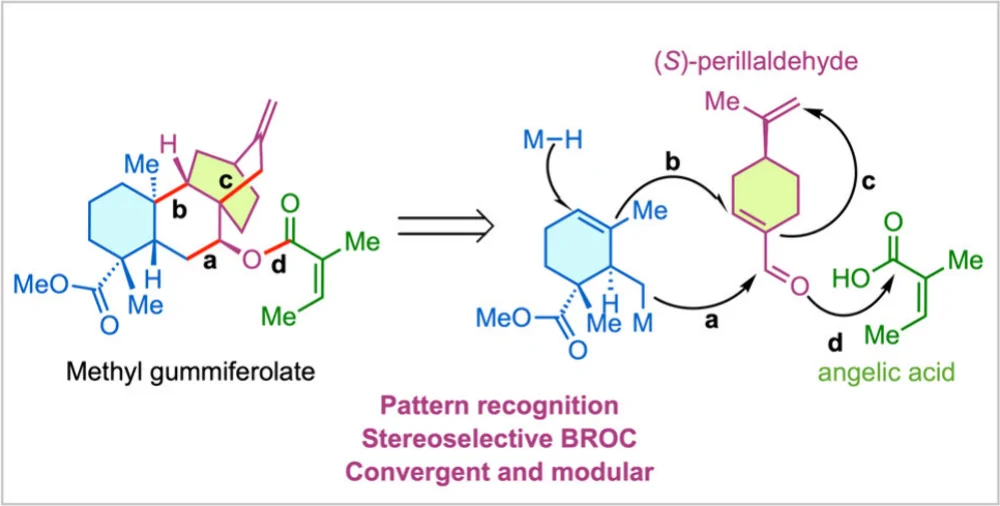

Gummiferolic acid (**1a**, Figure 1A) was isolated from *Margotia gummifera* in 1978 by Pinar and co-workers.^1^ It belongs to the *ent*-atisane diterpenoids, a large family of natural products that have attracted a significant amount of synthetic and medicinal interest.^2^ Structurally, gummiferolic acid contains a characteristic tetracyclic carbon skeleton with a *trans* decalin AB ring system fused with a bicyclo[2.2.2]­octane CD ring system and an angelate group at C7 of the B ring. In addition, it has seven stereogenic centers, three of which are all-carbon quaternary centers. Biosynthetically, like many other diterpene natural products, gummiferolic acid is synthesized from geranylgeranyl pyrophosphate (GGPP) via a series of cationic cyclizations and carbon skeleton rearrangements. Originally, the *ent*-atisane core of gummiferolic acid (cf. **2**) was proposed to be derived from the beyeranyl cation (**3**) via a 1,3-hydride shift from C12 to C13 followed by C13-to-C12 alkyl group migration.^3^ However, quantum chemical calculations conducted by Tantillo and Hong revealed that secondary carbocation intermediates such as **3** and the subsequent C12-to-C13 1,3-hydride shift intermediate are likely to resemble transition-state structures rather than intermediates.^4^ In addition, they suggested that **2** could be biosynthesized from kauranyl cation **4** via a concerted process which involves C12-to-C16 alkyl shift, C12-to-C13 1,3-hydride shift, and C13-to-C12 alkyl group migration. Loss of a proton from carbocation intermediate **2** followed by C–H oxidations would lead to gummiferolic acid and its natural congeners. Biologically, gummiferolic acid has demonstrated similar or even superior activity to gibberellic acid in regulating plant growth.^5^ Gummiferolic acid has also been used as an important starting point in microbiological transformations to access other analogues.^6^ These biological promises coupled with its complex and unique structural features make gummiferolic acid and its derivatives appealing targets for total synthesis practices.

**Figure 1:**
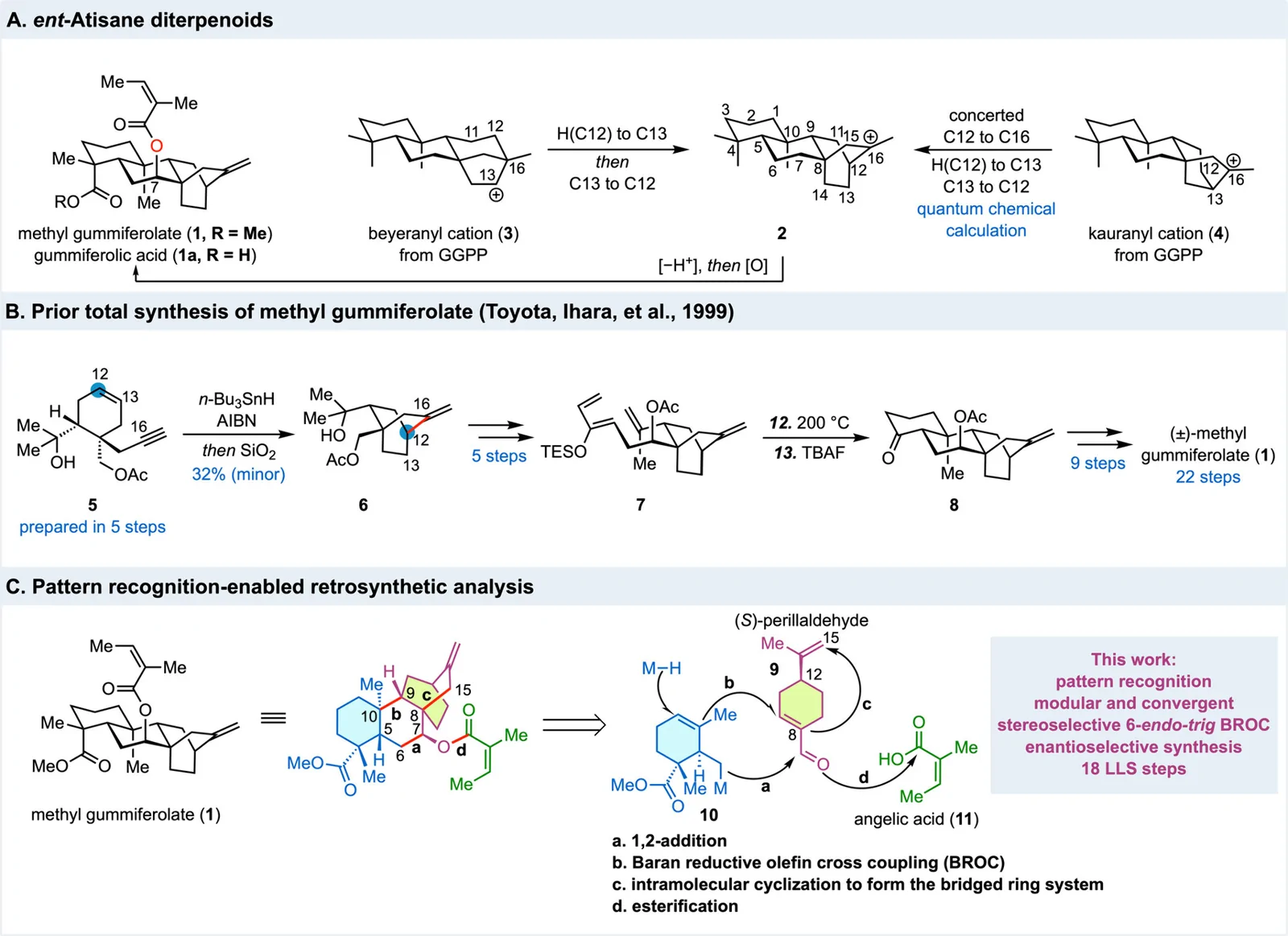
Structures, bioskeletal rearrangement, prior synthesis, and retrosynthetic analysis.

Interestingly, despite many recent efforts concentrated on the total synthesis of *ent*-atisane diterpenoids,^7,8^ there is only one reported total synthesis of the methyl ester form of gummiferolic acid (methyl gummiferolate, **1**) in racemic form (Figure 1B), which was completed by Toyota, Ihara, and co-workers in 1999.^9^ In their total synthesis, a tributyltin hydride-initiated radical cyclization was used to prepare the bicyclo[2.2.2]­octane CD ring system, but the desired product was produced as a minor product (**5**→**6**). Another key step in their total synthesis is an intramolecular Diels–Alder cycloaddition to build the AB decalin ring system. Overall, they prepared (±)-methyl gummiferolate in 22 steps. Herein, we report an enantioselective total synthesis of (−)-methyl gummiferolate.

Retrosynthetically (Figure 1C), using pattern recognition analysis,^10^ we disassembled methyl gummiferolate into three building blocks: (*S*)-perillaldehyde (**9**), a ten-carbon unit, an organometallic intermediate (**10**), another ten-carbon unit, and angelic acid (**11**) as the ester side chain. We proposed a convergent approach to unite the two ten-carbon units **9** and **10** via a nucleophilic 1,2-addition (step a) to form the bond between C6 and C7 and complete the twenty-carbon assembly for the total synthesis. Our recent interest^11^ in using metal-hydride hydrogen atom transfer (MHAT)-initiated radical cyclization for strategic bond formations in natural product total synthesis prompted us to explore the possibility of using the Baran reductive olefin cross coupling (BROC)^12^ to close the six-membered B ring by forming the bond between C10 and C9 via a 6-*endo-trig* cyclization (step b).^13^ Since we planned to use all the ten carbon atoms of (*S*)-perillaldehyde (**9**), it would then require a C–C bond formation between C15 and C8 to construct the bicyclo[2.2.2]­octane ring system (step c), while such a ring system is often formed by Diels–Alder cycloadditions.^7,8^ We anticipated that the *exo*-methylene and the carbonyl functionality of (*S*)-perillaldehyde would allow us to explore various intramolecular cyclizations for this bond formation. In the end, appending the angelic acid at C7 (step d) would complete the total synthesis.

Our exploration started with known compound **12** (86% ee, Scheme 1), which was readily prepared via an enantioselective Diels–Alder cycloaddition between the Rawal diene and methacrolein followed by acetal formation.^14^ Compound **12** was then advanced to iodide **17** for the subsequent coupling with a (*S*)-perillaldehyde derivative **19**. Johnson α-iodination^15^ followed by Suzuki–Miyaura cross coupling^16^ converted **12** to **13** in 94% yield. To introduce the iodomethyl group at C5 and control the corresponding stereochemistry, CBS reduction^17^ was used to reduce the ketone of **13** and give **14** in 98% yield and 4.5/1 diastereoselectivity. The resulting alcohol was then alkylated with tributyl (iodomethyl)­stannane (**15**).^18^ After lithium-stannane exchange, 2,3-Wittig–Still rearrangement^19^ occurred to provide **16** in 48% yield from **14**. This two-step sequence introduced a hydroxymethyl group at C5 with high fidelity in chirality transfer. Primary alcohol **16** was then converted to primary iodide **17** via Appel reaction in 90% yield. After treating **17** with *t*-BuLi, lithium-iodide exchange took place to afford alkyl lithium intermediate **18**. Meanwhile, (*S*)-perillaldehyde (**9**) was advanced to Weinreb amide **19** in three steps including oxidation of its aldehyde to carboxylic acid, EDCI-promoted condensation of the resulting carboxylic acid with *N*-methoxymethylamine, and epoxidation of the *exo*-methylene with *m*-CPBA. While a 1:1 mixture of diastereomers was obtained for the epoxidation, it is inconsequential because the C16 stereochemistry will be eliminated at a later stage. In addition, with the epoxide, we hoped a tandem 6-*endo-trig* BROC and epoxide ring opening to build both the six-membered B ring and the bicyclo[2.2.2]­octane ring system.^13e,20^ Nucleophilic 1,2-addition of alkyl lithium **18** to **19** gave enone product **21** in 55% yield from **17** and set up the stage for the 6-*endo-trig* BROC. When **21** was treated with Fe­(dpm)_3_, PhSiH_3_ in EtOH at elevated temperature, monocyclization product **22** was obtained in 81% yield. While the enolate intermediate did not further react with the epoxide to form the bicyclo[2.2.2]­octane ring system, the C12 stereocenter and the C5 stereocenter worked together to ensure high stereochemical outcome at C10 and C9. Other conditions and substrates were also explored to realize the tandem sequence but were unsuccessful (see the Supporting Information) presumably due to the severe steric hindrance for a concave face alkylation within a substrate containing a *cis*-decalin AB ring system and an axial methyl group at C4. To close the bridged ring system, compound **22** was first advanced to enone **23** via α-selenation followed by oxidative elimination. This maneuver opens the AB ring system and makes the intramolecular alkylation more accessible. The epoxide group of **23** was then opened with MgI_2_ and the resulting tertiary alcohol was masked as TMS ether in a one-pot procedure to give primary iodide **24** in 81% yield. The latter was then treated with LDA to generate a lithium enolate, which upon adding HMPA cyclized with the primary iodide to close the bridged ring. After the TMS group was removed in the same pot by adding TBAF, **25** was successfully produced. Notably, the purity of **24** is critical for the alkylation step and attempts to convert **23** to **25** via an intramolecular epoxide ring opening were unsuccessful. In addition, **21** was converted to the corresponding alkyl iodide substrate to explore a tandem BROC reaction and intramolecular alkylation, but only the BROC product was obtained (see the Supporting Information). These results indicate that both the conformation of the tricyclic ring system and the identity of the electrophile are important for the intramolecular alkylation to form the bridged ring system.

**Scheme 1:**
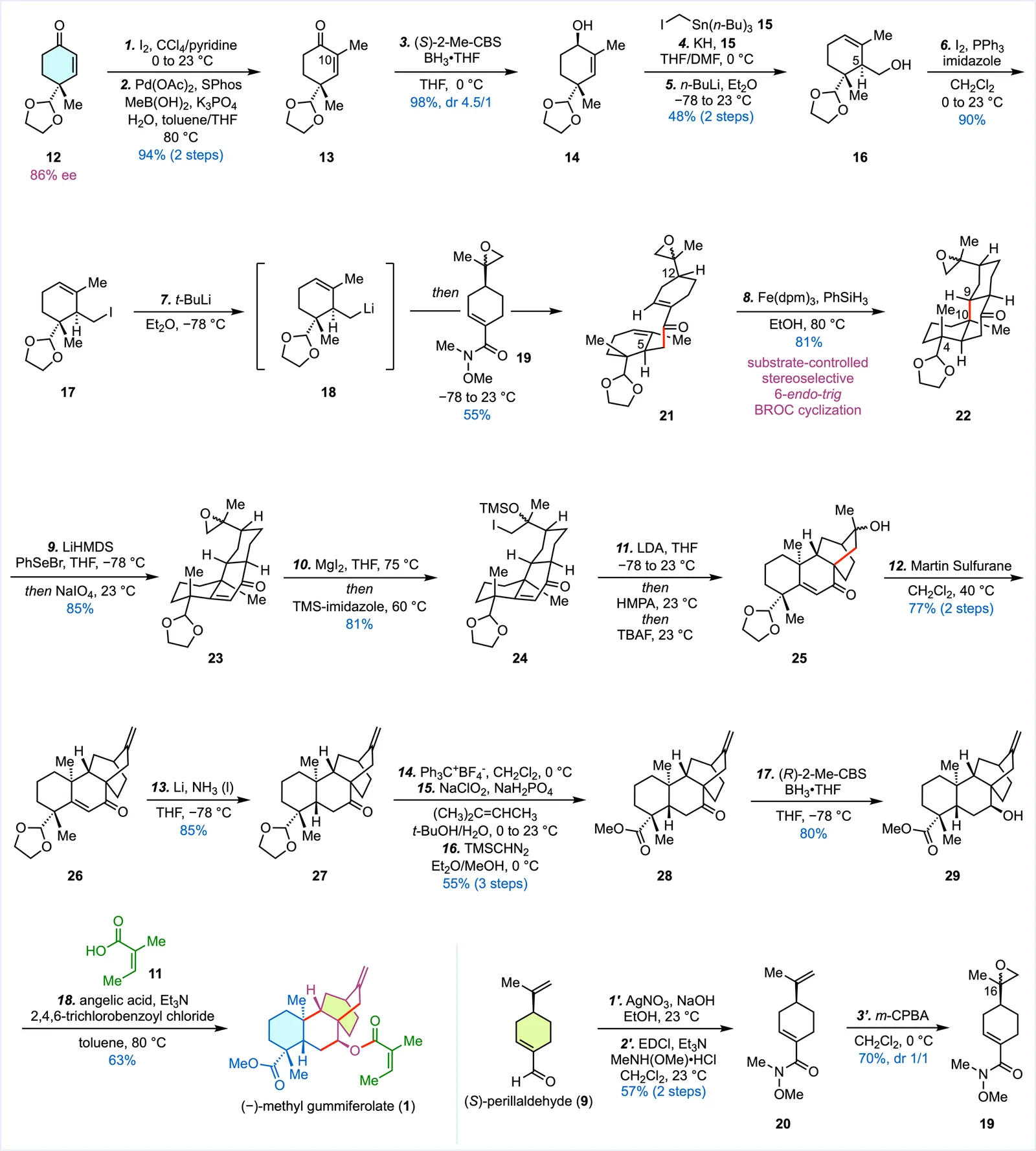
Total Synthesis of (−)-Methyl Gummiferolate

To continue the synthesis, compound **26** with the entire tetracyclic skeleton was produced in 77% yield from **24** with the Martin sulfurane-promoted dehydration.^21^ At this stage, Birch reduction of the enone functionality gave the desired *trans* decalin and provided **27** in 85% yield.^22^ The acetal group was next removed with tritylium tetrafluoroborate to release the aldehyde,^23^ which was next oxidized to the carboxylic acid with Pinnick oxidation. The resulting carboxylic acid was then treated with TMS diazomethane to give methyl ester **28** in 55% yield from **27**. To append the angelate, different conditions were explored to reduce ketone **28**. While most of the hydride-based and single-electron transfer reductions gave the undesired stereochemical outcome, CBS reduction with (*R*)-2-Me-CBS delivered **29** in 80% yield. Treating **29** with angelic acid (**11**) under the Yamaguchi conditions successfully completed the total synthesis of (−)-methyl gummiferolate (**1**).

In summary, an asymmetric total synthesis of (−)-methyl gummiferolate was completed in 18 steps from a readily available starting material, which was prepared via an enantioselective Diels–Alder reaction using the Rawal diene. The use of the ten-carbon (*S*)-perillaldehyde as a precursor of the bicyclo[2.2.2]­octane moiety is novel and different from the previous approaches in constructing such bridged ring systems. This strategy could be extended to the total synthesis of natural products including other *ent*-atisane diterpenoids with a similar bicyclo[2.2.2]­octane moiety. The MHAT-initiated radical cyclization, namely the BROC reaction, enabled an efficient construction of the six-membered B ring via a stereoselective 6-*endo-trig* cyclization, the stereochemistry of which was controlled by the existing stereocenter (C12) of (*S*)-perillaldehyde and C5. Other key steps of this synthesis include a combination of CBS reduction and 2,3-Wittig–Still rearrangement to introduce a hydroxymethyl group, a Weinreb ketone synthesis to unite two ten-carbon building blocks in a highly convergent manner, and an intramolecular α-alkylation to form the bicyclo[2.2.2]­octane moiety. Overall, this synthesis is modular and convergent and could be adapted for other *ent*-atisane diterpene synthesis.

## Supplementary Material



## Notes

Experimental procedures and NMR spectra for all new compounds (PDF)

## Abbreviations

MHAT: metal-hydride hydrogen atom transfer
*t*-BuLi: tert-butyllithium
EDCI: N-(3-(dimethylamino)­propyl)­prop-1-ene-1,1-diamine hydrochloride
*m*-CPBA: *meta*-chloroperoxybenzoic acid
Fe­(dpm)_3_: Iron tris­(2,2,6,6-tetramethyl-3,5-heptanedionate)
PhSiH_3_: phenylsilane
TMS: trimethylsilyl
LDA: lithium diisopropylamide
HMPA: hexamethylphosphoramide
TBAF: tetra-*n*-butylammonium fluoride
