# Supplementary material for: Convergent Total Synthesis of (−)-Methyl Gummiferolate Enabled by Pattern Recognition and Radical Cyclization
Source: J Am Chem Soc. 2026 Sep 28;148(40):42898–903. doi: 10.1021/jacs.6c15246 (PMC13674072; doi:10.1021/jacs.6c15246)
Supplement: Supplementary file 1 [file ja6c15246_si_001.pdf]

Supporting Information

Convergent Total Synthesis of (−)-Methyl Gummiferolate Enabled by Pattern Recognition and Radical Cyclization

Weizhao Zhao,[1] Jingsong Zhen,[1] and Mingji Dai*,[1,2]

[1]Department of Chemistry, Emory University, Atlanta, Georgia 30322, United States

[2]Department of Pharmacology and Chemical Biology, School of Medicine, Emory University, Atlanta, Georgia 30322, United States

Table of Contents

Part 1. Experimental Procedures and Spectra Data.......S3
Part 2. Natural Product NMR Comparison.......S23
Part 3. References.......S26
Part 4. ^{1}H and ^{13}C NMR Spectra.......S27

Part 1. Experimental Procedures and Spectra Data

A. General Methods

All commercially available compounds were purchased from Sigma-Aldrich, Alfa-Aesar, Oakwood chemicals, Ambeed, and ChemScene unless otherwise noted. Materials obtained from commercial suppliers were used without further purification. NMR spectra were recorded on Bruker spectrometers (^{1}H at 400 MHz, 600 MHz, 800 MHz and ^{13}C at 101 MHz, 151 MHz, 201 MHz). Chemical shifts (δ) were given in ppm with reference to solvent signals [$CDCl_3$ δ 7.26 ppm ^{1}H NMR, δ 77.2 ppm ^{13}C NMR); CD_3OD, δ 3.31 ppm ^{1}H NMR, δ 49.0 ppm ^{13}C NMR]. ^{1}H NMR data are reported as follows: chemical shift (δ ppm), multiplicity (s = singlet, d = doublet, t = triplet, q = quartet, m = multiplet, br = broad), coupling constant (Hz), and integration. IR spectra were collected on a Nicolet iS10 FT-IR spectrometer. Mass spectra were taken on a Thermo Finnigan LTQ-FTMS spectrometer with APCI, ESI. Optical rotation was determined by Autopol IV (Rudolph Research Analytical). Column chromatography was performed on silica gel. All reactions sensitive to air or moisture were conducted under argon atmosphere in dry solvents under anhydrous conditions, unless otherwise noted. Dry THF (tetrahydrofuran), DMF (dimethylformamide), DCM (CH_2Cl_2) were processed via PureProcessTechnology GS-SPS-5-CM system. All other solvents and reagents were used as obtained from commercial sources without further purification. Room temperature (r.t.) is around 23 °C.

B. Experiment Procedure and Spectra Data

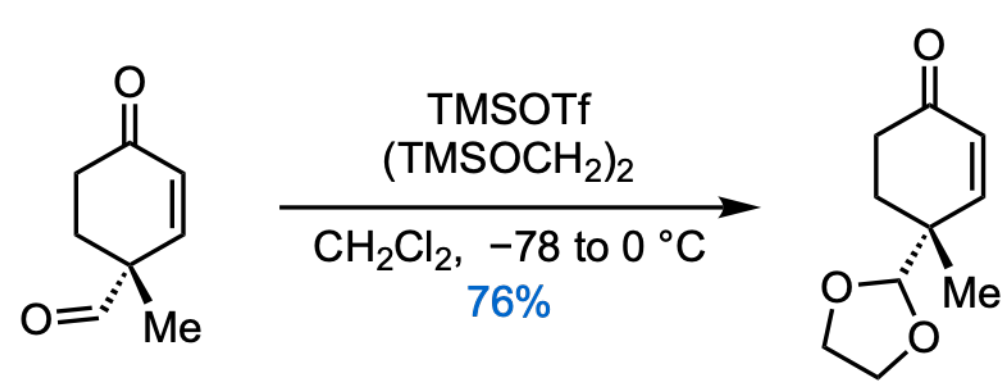

S1 12

Compound 12. To a solution of aldehyde **S1** [1] (3.1 g, 22.44 mmol, 1.0 equiv.) in dry DCM (224 mL) was added TMSOTf (0.41 mL, 2.24 mmol, 0.1 equiv) at −78 °C, after 5 min, $(TMSOCH_2)_2$ (6.6 mL, 26.93 mmol, 1.2 equiv.) was added. The reaction mixture was stirred for 30 min at −78 °C then warmed to 0 °C and stirred for another 30 min, The reaction was quenched with saturated aqueous $NaHCO_3$ (100 mL), and the resulting solution was extracted with DCM (100 mL × 3). The combined organic phases were washed with brine (150 mL), dried over Na_2SO_4, filtered and concentrated under reduced pressure. The crude

residue was purified by flash chromatography (20~30% ethyl acetate in hexane) to afford known compound **12** (3.1 g, 76%, 86% ee) as a colorless oil. [1b]

Compound 12:

R_f = 0.36 (hexane/ethyl acetate = 3/1).

$[\alpha]_D^{23}$ = +23.6 (c = 0.1 in $CHCl_3$).

IR (film) λ_{max}: 2962, 2929, 1644, 1436, 1374, 1184, 1107, 969, 885 cm^{-1}.

^{1}H NMR (400 MHz, $CDCl_3$) δ 6.77 (dd, J = 10.3, 1.2 Hz, 1H), 5.99 (d, J = 10.2 Hz, 1H), 4.74 (s, 1H), 4.02 – 3.85 (m, 4H), 2.65 – 2.38 (m, 2H), 2.16 (ddd, J = 13.6, 10.2, 5.4 Hz, 1H), 1.84 – 1.74 (m, 1H), 1.18 (s, 3H).

^{13}C NMR (101 MHz, $CDCl_3$) δ 199.6, 153.5, 129.5, 108.2, 65.7, 65.5, 40.2, 33.9, 29.9, 20.3.

HRMS m/z (ESI): calc. for $C_{10}H_{14}O_3Na^+$ $[M+Na]^+$: 205.0835, found: 205.0836.

HPLC Chromatogram: **12** (racemic)

```
==================================================================
Acq. Operator   : SYSTEM                          Seq. Line :   3
Acq. Instrument : Agilent 1100                     Location :   52
Injection Date  : 4/28/2026 12:03:27 PM                 Inj :   1
                                               Inj Volume : 10.000 µl
Method          : C:\Chem32\1\Data\28-Apr-2026\28-Apr-2026 2026-04-28 11-31-24\ASH_60min_1.0ML_
                  20%_2024.M (Sequence Method)
Last changed    : 4/28/2026 11:19:59 AM by SYSTEM
Additional Info : Peak(s) manually integrated
```

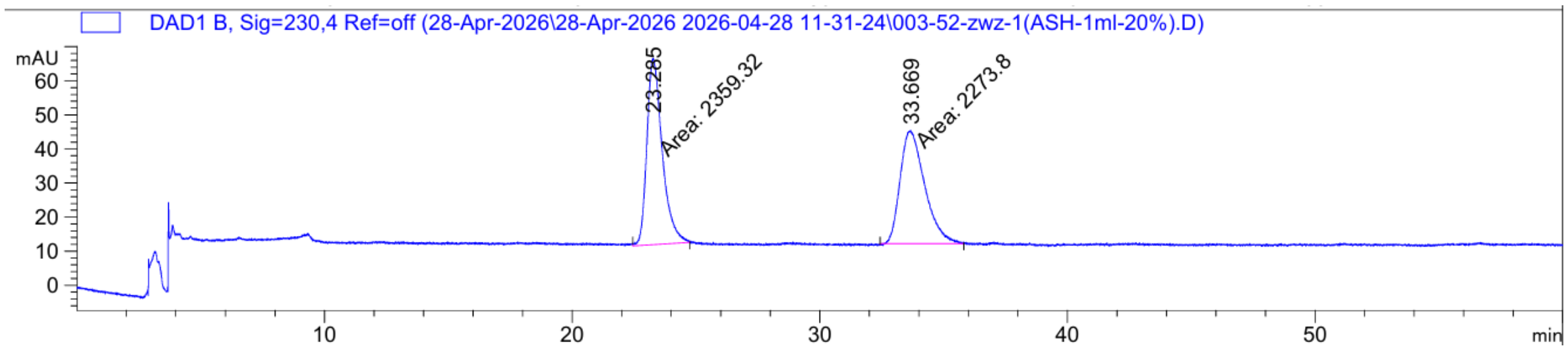

Signal 2: DAD1 B, Sig=230,4 Ref=off

Peak #	RetTime [min]	Type	Width [min]	Area [mAU*s]	Height [mAU]	Area %
1	23.285	MM	0.7182	2359.31714	54.75434	50.9229
2	33.669	MM	1.1399	2273.80151	33.24434	49.0771
Totals :				4633.11865	87.99868	

HPLC Chromatogram: **12** (enantioenriched)

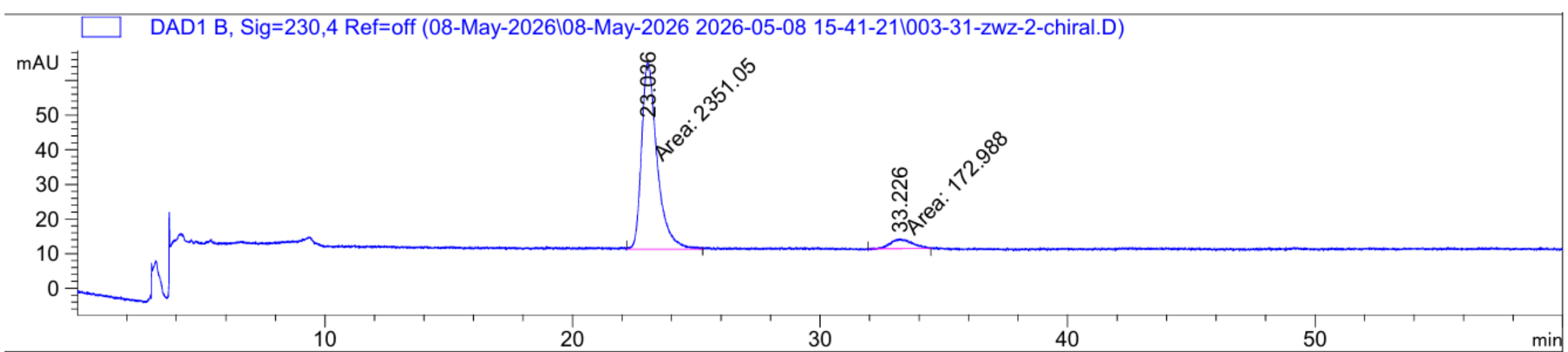

Signal 2: DAD1 B, Sig=230,4 Ref=off

Peak #	RetTime [min]	Type	Width [min]	Area [mAU*s]	Height [mAU]	Area %
1	23.036	MM	0.7284	2351.04639	53.79557	93.1464
2	33.226	MM	1.0006	172.98792	2.88143	6.8536
Totals :				2524.03430	56.67700	

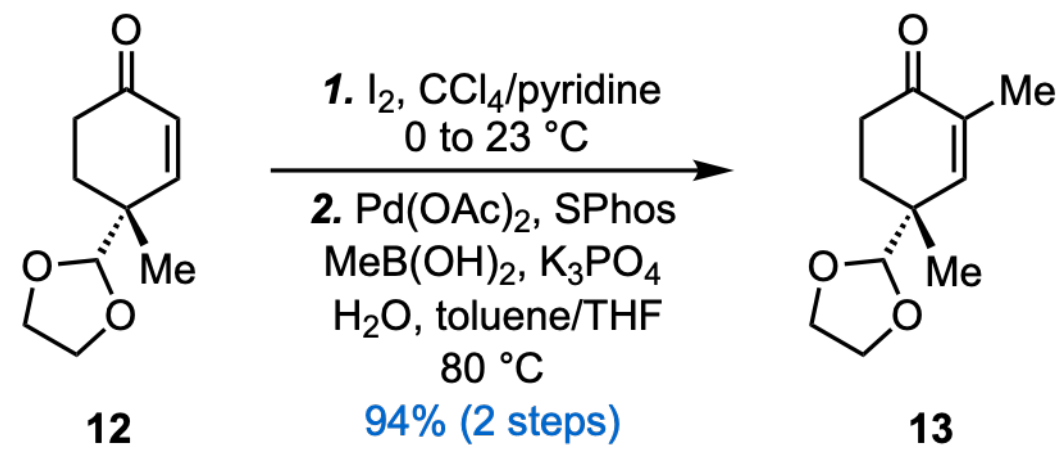

Compound 13. To a solution of **12** (3.0 g, 16.48 mmol, 1.0 equiv.) in CCl_4/pyridine (8 mL/8 mL) was slowly added a solution of I_2 (6.27 g, 24.72 mmol, 1.5 equiv.) in CCl_4/pyridine (8 mL/8 mL) at 0 °C. The resulting mixture was gradually warmed to room temperature and stirred for overnight. Upon complete consumption of the starting material as indicated by TLC, the reaction was quenched with saturated aqueous $Na_2S_2O_3$ (80 mL). The resulting mixture was extracted with EtOAc (50 mL × 3). The combined organic phases were washed with brine (100 mL), dried over anhydrous Na_2SO_4, filtered and concentrated under reduced pressure. The crude residue was purified briefly by a short flash chromatography on silica gel (10% ~ 30% ethyl acetate in hexane) to give the iodination product which was used for next step without further purification because of its limited stability.

To a solution of the above product in toluene/THF (160 mL/20 mL) was added SPhos (1.35 g, 3.3 mmol, 0.2 equiv.), K_3PO_4 (14.0 g, 65.92 mmol, 4.0 equiv.), methylboronic acid (5.12 mL, 82.4 mmol, 5.0 equiv.), $Pd(OAc)_2$ (370.4 mg, 1.65 mmol, 0.1 equiv.) and H_2O (3 mL, 164.8 mmol, 10.0 equiv.) at room temperature. The resulting mixture was degassed with an argon balloon for 10 minutes before it was moved to a preheated 80 °C oil bath and stirred for 2 h. Upon complete consumption of the starting material as indicated by TLC, the reaction was quenched with saturated aqueous $NaHCO_3$ (100 mL). The resulting mixture was extracted with EtOAc (80 mL × 3). The combined organic phases were washed with brine (100 mL), dried over anhydrous Na_2SO_4, filtered and concentrated under reduced pressure. The crude residue was purified by flash chromatography (10% ~30% ethyl acetate in hexane) to afford **13** (3.04 g, 94%) as a colorless oil.

Compound 13:

R_f = 0.36 (hexane/ethyl acetate = 3/1).

$[\alpha]_D^{23}$ = +8.0 (c = 0.5 in $CHCl_3$).

IR (film) λmax: 2954, 2924, 2850, 1675, 1109, 1097, 1035, 783, 762 cm-1.

^{1}H NMR (400 MHz, $CDCl_3$) δ 6.50 (t, J = 1.4 Hz, 1H), 4.70 (s, 1H), 3.99 – 3.84 (m, 4H), 2.59 – 2.39 (m, 2H), 2.11 (ddd, J = 13.5, 10.3, 5.5 Hz, 1H), 1.81 – 1.72 (m, 1H) (overlap with CH_3), 1.77 (d, J = 1.4 Hz, 3H) 1.13 (s, 3H).

^{13}C NMR (101 MHz, $CDCl_3$) δ 199.8, 148.7, 135.4, 108.4, 65.6, 65.5, 40.3, 34.0, 30.1, 20.3, 16.5.

HRMS m/z (ESI): calc. for $C_{11}H_{16}O_3{}^{23}Na^+$ $[M+Na]^+$: 219.0992, found: 219.0983.

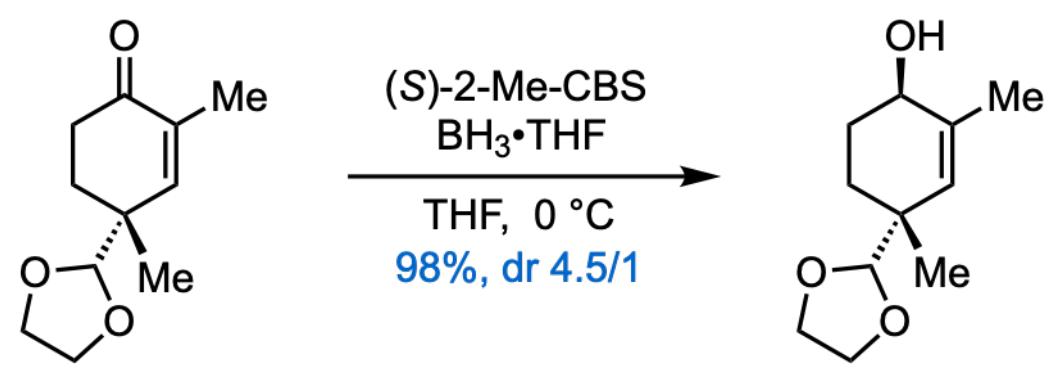

13 **14**

Compound 14. To a mixture of **13** (4.57 g, 23.3 mmol, 1.0 equiv.) and (*S*)-2-Me-CBS (1.94 g, 6.99 mmol, 0.3 equiv.) in THF (233 mL) was added dropwise a solution of $BH_3{\cdot}THF$ (1.0 M in THF, 27.96 mL, 27.96 mmol, 1.2 equiv.) at 0 °C. The resulting mixture was stirred for 10 min at 0 °C. Upon complete consumption of the starting material as indicated by TLC, the reaction was carefully quenched with saturated aqueous $NaHCO_3$ (150 mL). The resultant mixture was extracted with EtOAc (3 × 80 mL). The combined organic phases were washed with brine (150 mL), dried over anhydrous Na_2SO_4, filtered and concentrated under reduced pressure. The crude residue was purified by flash chromatography (50% ethyl acetate in hexane) to afford a mixture of diastereomers (4.52 g, 98%, 4.5/1 dr). The mixture of diastereomers was further purified by flash chromatography (20% ~50% ethyl acetate in hexane) to afford **14** as a pale-yellow oil.

Note: *The recovered minor isomer was oxidized with DMP to regenerate the starting material and used in the next reaction cycle.*

Compound 14:

R_f = 0.40 (hexane/ethyl acetate = 2/1).

$[\alpha]_D^{23}$ = +17.2 (*c* = 0.5 in $CHCl_3$).

IR (film) λ_{max}: 2935, 2867, 1134, 1098, 1026, 971, 860, 832 cm^{-1}.

^{1}H NMR (600 MHz, $CDCl_3$) δ 5.32 (q, *J* = 1.3 Hz, 1H), 4.57 (s, 1H), 4.02 (t, *J* = 6.0 Hz, 1H), 3.97 – 3.88 (m, 2H), 3.88 – 3.80 (m, 2H), 2.06 – 1.96 (m, 1H), 1.79 (dd, *J* = 1.5, 0.8 Hz, 3H), 1.70 (ddd, *J* = 13.1, 10.0, 3.1 Hz, 1H), 1.66 – 1.59 (m, 1H), 1.53 (s, 1H), 1.50 – 1.42 (m, 1H), 1.00 (s, 3H).

^{13}C NMR (151 MHz, $CDCl_3$) δ 137.0, 129.4, 108.9, 69.2, 65.5, 65.4, 39.4, 29.1, 27.3, 21.7, 20.4.

HRMS m/z (APCI): calc. for $C_{11}H_{19}O_3^+$ [M+H]$^+$: 199.1329, found: 199.1334.

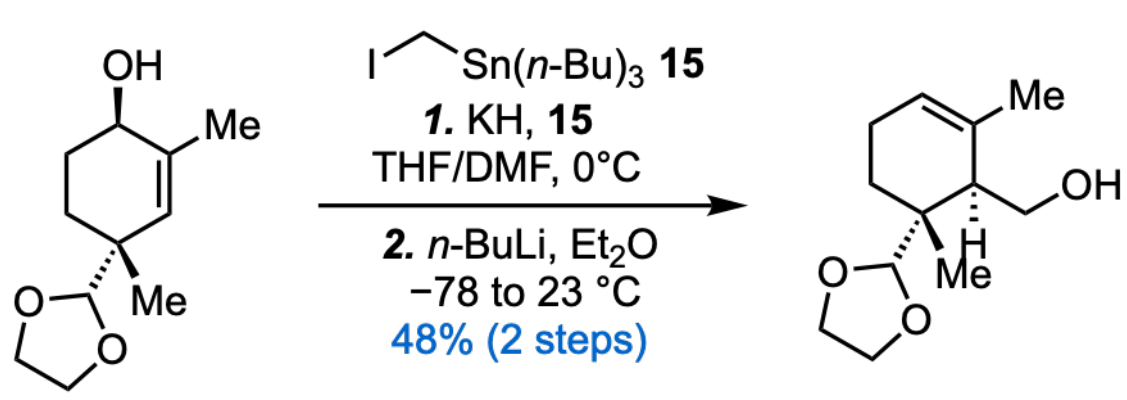

14 **16**

Compound 16. To a mixture of KH (30 wt % dispersion in mineral oil, 2.37 g, 59.06 mmol, 3.0 equiv.) in THF (20 mL) and DMF (40 mL) was added dropwise a solution of **14** (3.9 g, 19.68 mmol, 1.0 equiv.) in THF (10 mL) at 0 °C. The reaction mixture was stirred at 0 °C for 45 min, then $ICH_2Sn(n\text{-}Bu)_3$ (9.33 g, 21.65 mmol, 1.1 equiv.) was added. The reaction mixture was stirred for additional 1 h at 0 °C. Upon complete consumption of the starting material as indicated by TLC, the resultant mixture was carefully quenched with saturated aqueous $NaHCO_3$ (50 mL) and extracted with EtOAc (3 × 50 mL). The combined organic phases were washed with brine (80 mL), dried over anhydrous Na_2SO_4, filtered and concentrated under reduced pressure. The crude residue was purified by flash chromatography (5% ~ 10% ethyl acetate in hexane) to afford the organotin reagent which was used for next step without further purification because of its limited stability.

To a solution of the above product in Et_2O (196 mL) was added *n*-BuLi (2.5 M in Hexane, 9.5 mL, 23.62 mmol, 1.2 equiv.) at −78 °C. The reaction mixture was then slowly warmed to room temperature and stirred for 3 h before it was carefully quenched with saturated aqueous $NaHCO_3$ (50 mL) and extracted with EtOAc (3 × 100 mL). The combined organic phases were washed with brine (150 mL), dried over anhydrous Na_2SO_4, filtered and concentrated under reduced pressure. The crude residue was purified by flash chromatography (10% ~ 50% ethyl acetate in hexane) to afford **16** (2.0 g, 48% for 2 steps) as colorless oil.

Compound 16:

$\mathbf{R_f}$ = 0.25 (hexane/ethyl acetate = 2/1).

$[\alpha]_D^{23}$ = −47.7 (*c* = 0.3 in $CHCl_3$).

IR (film) λ_{max}: 2883, 1451, 1396, 1225, 1146, 1099, 1074, 1027 cm^{-1}.

^{1}H NMR (600 MHz, CDCl$_3$) δ 5.72 – 5.64 (m, 1H), 4.77 (s, 1H), 4.00 – 3.82 (m, 4H), 3.80 (ddd, *J* = 16.3, 10.0, 5.3 Hz, 2H), 2.15 – 2.11 (m, 1H), 2.11 – 1.97 (m, 2H), 1.76 (d, *J* = 1.8 Hz, 3H), 1.71 – 1.58 (m, 3H), 0.97 (s, 3H).

^{13}C NMR (151 MHz, CDCl$_3$) δ 131.6, 125.9, 106.8, 65.4, 65.2, 61.1, 46.6, 39.0, 28.6, 22.8, 22.4, 17.1.

HRMS m/z (ESI): calc. for $C_{12}H_{20}O_3Na^+$ $[M+Na]^+$: 235.1305, found: 235.1303.

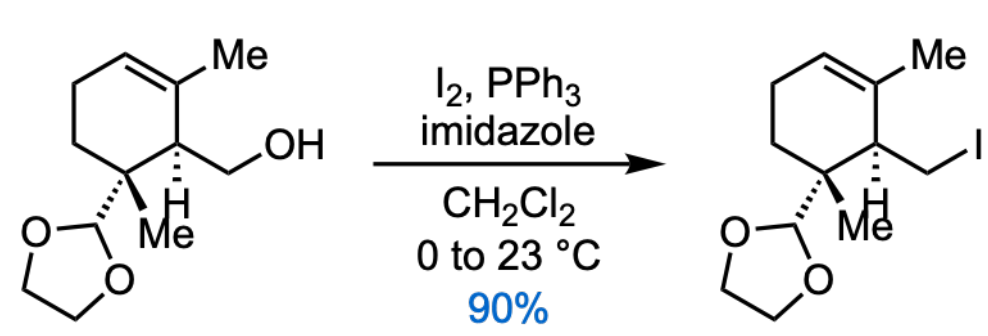

16 17

Compound 17. To a mixture of imidazole (456 mg, 6.74 mmol, 1.3 equiv.) and PPh_3 (1.77 g, 6.74 mmol, 1.3 equiv.) in DCM (10 mL) was added dropwise a solution of I_2 (1.58 g, 5.22 mmol, 1.2 equiv.) in DCM (35 mL) at 0 °C. The reaction mixture was stirred for 10 min, followed by the dropwise addition of a solution of **16** (1.1 g, 5.19 mmol, 1.0 equiv.). The resulting mixture was then warmed to room temperature and stirred for 48 h. Upon complete consumption of the starting material as indicated by TLC, the reaction was quenched with saturated aqueous $NaHCO_3$ (50 mL). The resultant mixture was extracted with DCM (3 × 100 mL). The combined organic phase was washed with brine (50 mL), dried over anhydrous Na_2SO_4, filtered and concentrated under reduced pressure. The crude residue was purified by flash chromatography (5% ~ 10% ethyl acetate in hexane) to afford **17** (1.5 g, 90%) as colorless oil.

Compound 17:

R_f = 0.35 (hexane/ethyl acetate = 10/1).

$[\alpha]_D^{23}$ = −87.1 (c = 0.5 in $CHCl_3$).

IR (film) λ_{max}: 2935, 2882, 1467, 1451, 1394, 1282, 1166, 1094, 972, 876 cm^{-1}.

^{1}H NMR (400 MHz, $CDCl_3$) δ 5.64 – 5.51 (m, 1H), 4.73 (s, 1H), 4.00 – 3.78 (m, 4H), 3.50 – 3.30 (m, 2H), 2.29 (t, J = 3.9 Hz, 1H), 2.03 – 1.96 (m, 2H), 1.89 – 1.80 (m, 1H), 1.75 (d, J = 1.9 Hz, 3H), 1.49 (dt, J = 13.6, 4.6 Hz, 1H), 1.00 (s, 3H).

^{13}C NMR (101 MHz, $CDCl_3$) δ 133.7, 124.4, 106.5, 65.4, 65.2, 44.1, 39.9, 26.3, 22.8, 22.2, 16.8, 7.5.

HRMS m/z (APCI): calc. for $C_{12}H_{19}IO_2^+$ $[M+H]^+$: 323.0503, found: 323.0500.

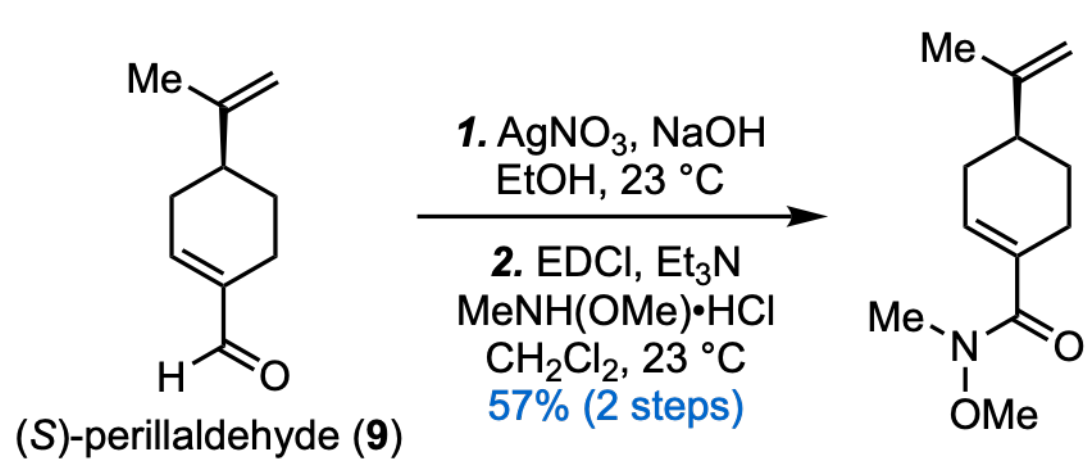

20

Compound 20. To a solution of $AgNO_3$ (11.33 g, 66.66 mmol, 2.0 equiv.) in H_2O (60 mL) was added a solution of NaOH (5.33 g, 13.33 mmol, 0.4 equiv.) in H_2O (60 mL) at room temperature. The resulting brown suspension was stirred for 5 min at room temperature and a solution of (S)-perillaldehyde (5.0 g, 33.33 mmol, 1.0 equiv.) in EtOH (70 mL) was added. The reaction mixture was stirred for 3 h and then filtered. The filtrate was diluted with water, washed with EtOAc (100 mL), acidified with hydrochloric acid and extracted with EtOAc (50 mL × 3). The combined organic phases were washed with brine (150 mL), dried over Na_2SO_4, filtered and concentrated under reduced pressure to obtain (S)-perillic acid as a white solid.[2] The crude product was used directly in the next step without further purification.

To a mixture of the above crude (*S*)-perillic acid and MeNH(OMe)·HCl (3.6 g, 36.66 mmol, 1.1 equiv.) in DCM (167 mL) was added EDCI (7.03 g, 36.66 mmol, 1.1 equiv.) and Et_3N (5.1 mL, 36.66 mmol, 1.1 equiv.) at room temperature. The resulting mixture was stirred for 2 h at room temperature. Upon complete consumption of the starting material as indicated by TLC, the reaction mixture was quenched with saturated aqueous $NaHCO_3$ (100 mL). The resulting mixture was extracted with EtOAc (60 mL × 3). The combined organic phases were washed with brine (100 mL), dried over anhydrous Na_2SO_4, filtered and concentrated under reduced pressure. The crude residue was purified by flash chromatography (30% ~ 50% ethyl acetate in hexane) to afford **20** (3.8 g, 57%) as a pale-yellow oil.

Compound 20:
R_f = 0.30 (hexane/ethyl acetate = 1/1).
$[\alpha]_D^{23}$ = −18.7 (c = 0.5 in $CHCl_3$).
IR (film) λ_{max}: 2962, 2929, 1644, 1436, 1374, 1184, 1107, 969, 885 cm^{-1}.
^{1}H NMR (400 MHz, $CDCl_3$) δ 6.20 – 6.15 (m, 1H), 4.74 – 4.71 (m, 1H), 4.70 – 4.67 (m, 1H), 3.62 (s, 3H), 3.20 (s, 3H), 2.38 – 2.28 (m, 2H), 2.31 – 2.13 (m, 2H), 2.10 – 1.97 (m, 1H), 1.90 – 1.79 (m, 1H), 1.72 (s, 3H), 1.55 – 1.42 (m, 1H).
^{13}C NMR (101 MHz, $CDCl_3$) δ 171.7, 149.1, 133.5, 130.7, 109.1, 61.2, 40.3, 33.7, 30.5, 27.2, 26.1, 20.8.
HRMS m/z (APCI): calc. for $C_{12}H_{20}O_2N^+$ $[M+H]^+$: 210.1489, found: 210.1491.

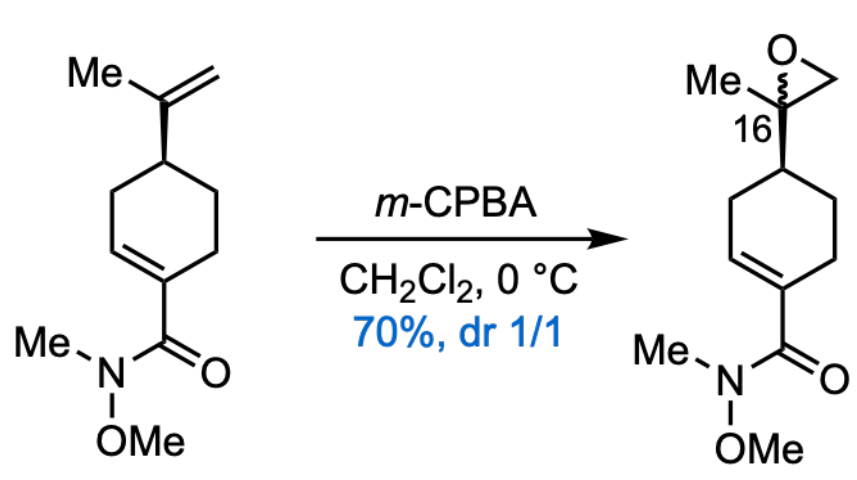

20 **19**

Compound 19. To a solution of **20** (3.3 g, 15.78 mmol, 1.0 equiv.) in DCM (80 mL) was slowly added a solution of *m*-CPBA (75%, 3.63 g, 15.78 mmol, 1.0 equiv) in DCM (80 mL) at 0 °C. The resulting mixture was stirred for 2 h at 0 °C. Upon complete consumption of the starting material as indicated by TLC, the reaction mixture was carefully quenched with saturated aqueous $NaHCO_3$ (200 mL) and stirred for 2 h at room temperature. The resulting solution was extracted with DCM (100 mL × 3). The combined organic phases were washed with brine (150 mL), dried over Na_2SO_4, filtered and concentrated under reduced pressure. The crude residue was purified by flash chromatography (20~40% ethyl acetate in hexane) to afford **19** (2.5 g, 70%, dr 1/1 at the epoxide carbon) as a colorless oil.

Note: *m-CPBA is explosive and should be used with caution!*

Compound 19 (inseparable mixture of diastereomers, dr 1/1):

$\mathbf{R_f}$ **=** 0.26 (hexane/ethyl acetate = 1/1).

$[\alpha]_D^{23}$ = −47.3 (*c* = 0.5 in $CHCl_3$).

IR (film) λ_{max}: 2930, 1626, 1437, 1379, 1357, 1185, 1146, 972, 904, 825, 763 cm^{-1}.

^{1}**H NMR (400 MHz, $CDCl_3$)** δ 6.19 – 6.10 (m, 1H), 3.60 (s, 3H), 3.18 (s, 3H), 2.65 – 2.61 (m, 1H), 2.55 (d, *J* = 7.0, 0.5H), 2.54 (d, *J* = 7.0, 0.5H), 2.40 – 2.11 (m, 3H), 2.08 – 1.81 (m, 2H), 1.62 – 1.47 (m, 1H), 1.49 – 1.26 (m, 1H), 1.25 (s, 3H).

^{13}**C NMR (101 MHz, $CDCl_3$)** δ 171.52, 171.49, 133.9, 133.7, 130.1, 129.9, 61.2, 59.1, 58.9, 53.1, 52.8, 39.4, 39.2, 33.7, 33.6, 27.24, 27.22, 26.0, 25.7, 24.41, 24.38, 18.5, 18.2.

HRMS m/z (APCI): calc. for $C_{12}H_{20}O_3N^+$ $[M+H]^+$: 226.1438, found: 226.1435.

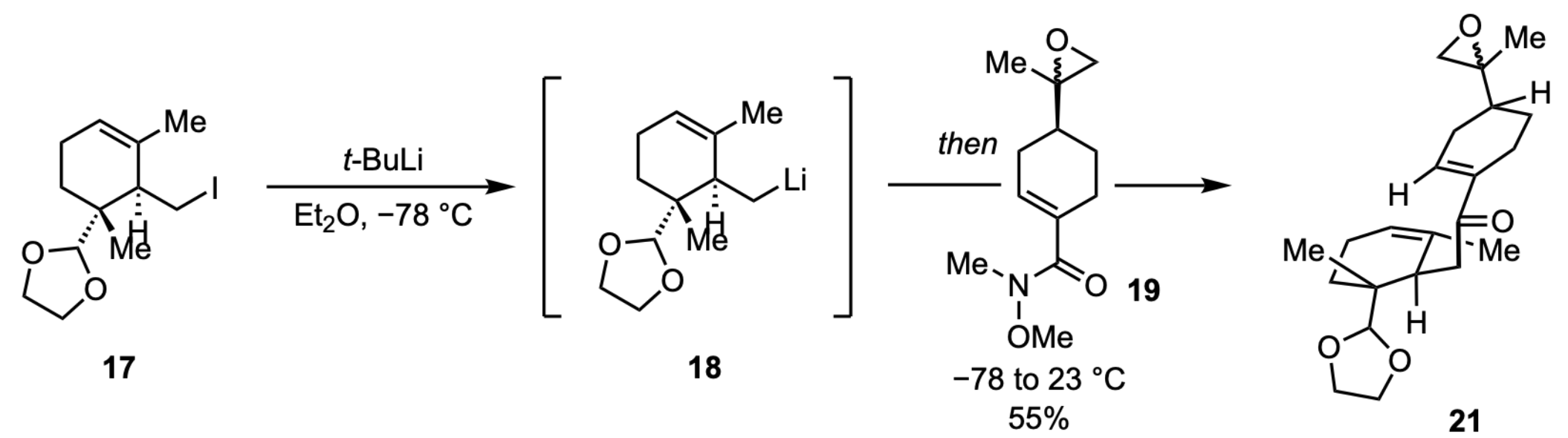

Compound 21. To a solution of **17** (1.36 g, 4.22 mmol, 1.0 equiv.) in Et_2O (28 mL) was added dropwise a solution of *t*-BuLi (1.7 M in pentane, 5.46 mL, 9.28 mmol, 2.2 equiv.) at −78 °C. The reaction mixture was stirred for 15 min at −78 °C, followed by dropwise addition of a solution of **19** (2.85 g, 12.66 mmol, 3.0 equiv.) in Et_2O (12 mL). After stirred at −78 °C for 1 h, the resulting mixture was slowly warmed to 23 °C and then quenched with saturated aqueous $NaHCO_3$ (30 mL). The resultant mixture was extracted with EtOAc (3 × 30 mL). The combined organic phases were washed with brine (100 mL), dried over anhydrous Na_2SO_4, filtered and concentrated under reduced pressure. The crude residue was purified by flash chromatography (10 ~ 30% ethyl acetate in hexane) to afford **21** (836 mg, 55%, dr 1/1 at the epoxide carbon) as a pale-yellow oil.

Compound 21 (inseparable mixture of diastereomers, dr 1/1 at the epoxide carbon):

$\mathbf{R_f}$ **=** 0.35 (hexane/ethyl acetate = 3/1).

$[\alpha]_D^{23}$ = −29.2 (*c* = 0.5 in $CHCl_3$).

IR (film) λ_{max}: 2924, 1668, 1643, 1456, 1380, 1172, 1144, 1097, 1036, 921, 850 cm^{-1}.

^{1}H NMR (400 MHz, CDCl$_3$) δ 6.92 – 6.85 (m, 1H), 5.42 – 5.34 (m, 1H), 4.78 (s, 1H), 3.97 – 3.76 (m, 4H), 3.00 – 2.82 (m, 2H), 2.68 – 2.64 (m, 1H), 2.61 – 2.47 (m, 2H), 2.42 (dt, J = 16.7, 4.3 Hz, 1H), 2.37 – 1.86 (m, 6H), 1.58 – 1.50 (m, 4H, overlap with Me), 1.47 – 1.31 (m, 2H), 1.30 (s, 1.5H), 1.29 (s, 1.5H), 1.26 – 1.19 (m, 1H), 0.71 (s, 3H).

^{13}C NMR (101 MHz, CDCl$_3$) δ 200.34, 200.30, 139.4, 139.3, 137.5, 137.2, 135.8, 121.9, 106.9, 65.4, 65.2, 59.1, 58.9, 53.2, 53.0, 39.4, 39.20, 39.17, 38.70, 38.67, 38.3, 28.1, 28.0, 27.6, 24.5, 24.4, 24.1, 23.7, 22.8, 22.4, 18.6, 18.4, 16.7.

HRMS m/z (APCI): calc. for $C_{22}H_{33}O_4^+$ $[M+H]^+$: 361.2373, found: 361.2377.

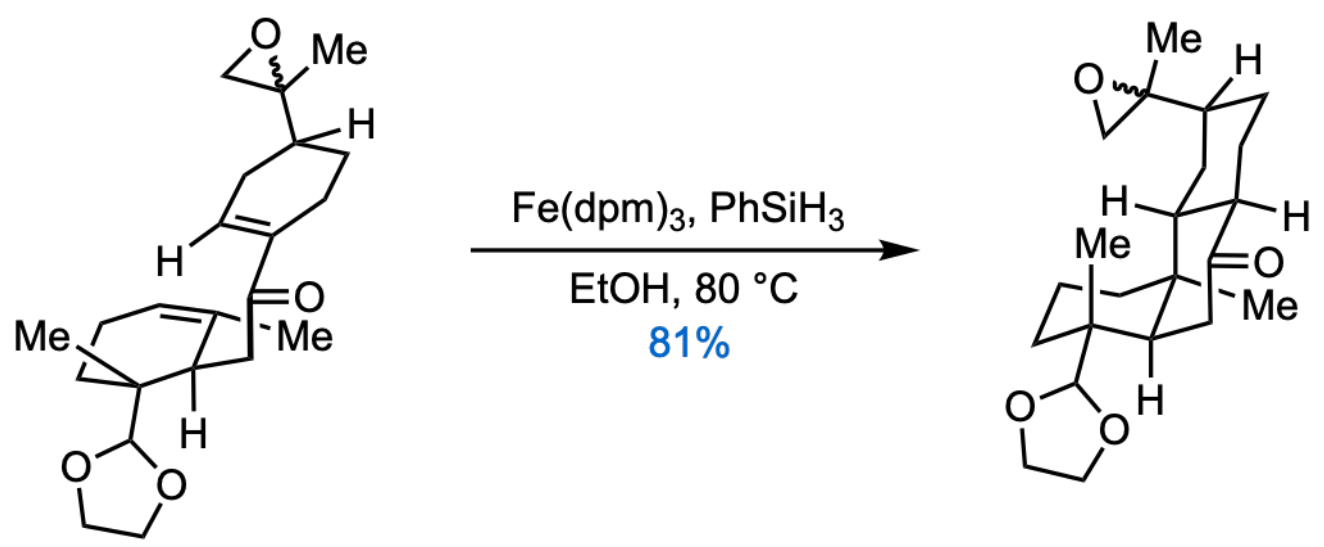

21 **22**

Compound 22. To a mixture of **21** (217 mg, 0.6 mmol, 1.0 equiv.) and Fe(dpm)$_3$ (364 mg, 0.6 mmol, 1.0 equiv.) in EtOH (12 mL) was added PhSiH$_3$ (223 μL, 1.8 mmol, 3.0 equiv.) at 80 ˚C. The resulting mixture was stirred for 12 h at 80 ˚C. Upon complete consumption of the starting material as indicated by TLC, the reaction mixture was filtered through a short silica gel column and washed with EtOAc. The combined filtrate was concentrated under reduced pressure. The crude residue was purified by flash chromatography (10 ~ 30% ethyl acetate in hexane) to afford product **22** (176 mg, 81%, dr 1/1 at the epoxide carbon) as a pale-yellow oil.

Compound 22 (inseparable mixture of diastereomers, dr 1/1 at the epoxide carbon):

R$_f$ = 0.34 (hexane/ethyl acetate = 3/1).

$[\alpha]_D^{23}$ = −18.3 (c = 0.2 in MeOH).

IR (film) λ_{max}: 2934, 2867, 1701, 1455, 1380, 1135, 1097, 1073, 985, 918 cm^{-1}.

^{1}H NMR (400 MHz, CDCl$_3$) δ 4.594 (s, 0.5H), 4.587 (s, 0.5H), 3.96 – 3.77 (m, 4H), 2.87 – 2.72 (m, 2H), 2.59 – 2.29 (m, 3H), 2.11 – 1.72 (m, 8H), 1.56 – 1.36 (m, 5H), 1.34 (s, 3H), 1.32 – 1.18 (m, 2H), 1.11 (s, 3 × 0.5H), 1.09 (s, 3 × 0.5H), 0.94 (s, 3 × 0.5H), 0.91 (s, 3 × 0.5H).

^{13}C NMR (101 MHz, CDCl$_3$) δ 213.7, 213.6, 109.80, 109.77, 65.3, 65.0, 59.8, 58.8, 52.1, 51.4, 49.6, 48.5, 48.4, 41.3, 39.13, 39.11, 38.4, 38.1, 36.4, 36.21, 36.17, 35.9, 35.6, 33.0, 28.33, 28.25, 26.6, 25.6, 24.6, 24.5, 22.7, 22.5, 22.3, 22.2, 18.2, 18.1, 17.0, 16.9.

HRMS m/z (ESI): calc. for $C_{22}H_{35}O_4^+$ $[M+H]^+$: 363.2530, found: 363.2531.

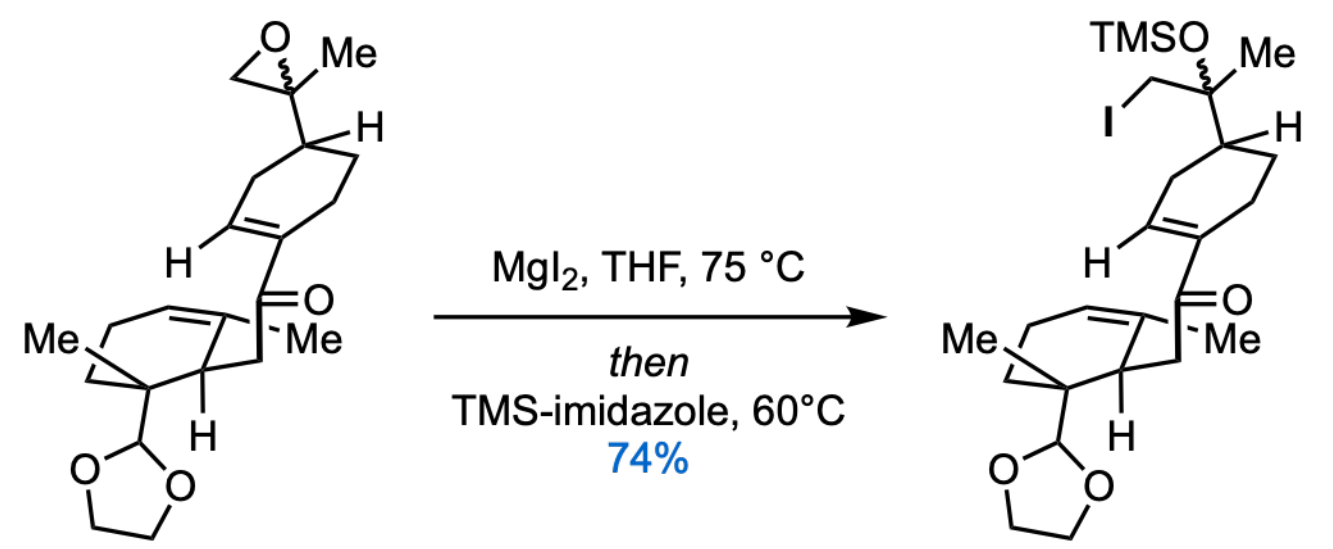

Compound S2. To a mixture of **21** (40 mg, 0.11 mmol, 1.0 equiv.) and MgI_2 (92 mg, 0.33 mmol, 3.0 equiv.) was added THF (2.2 mL,) at room temperature. The reaction mixture was heated to 75 °C with an oil bath and stirred for 30 min. Upon full consumption of the starting material as indicated by TLC, the reaction mixture was cooled to 60 °C. Then TMS-imidazole (161 µL, 1.1 mmol, 10.0 equiv.) was added dropwise and the reaction mixture was stirred at this temperature. Upon full consumption of the intermediate as indicated by TLC, the reaction was quenched with saturated aqueous $NaHCO_3$ (10 mL) and extracted with EtOAc (3 × 15 mL). The combined organic phases were dried over anhydrous Na_2SO_4, filtered and concentrated under reduced pressure. The crude residue was purified by flash column chromatography on silica gel (10 ~ 20% ethyl acetate in hexane) to afford **S2** (46 mg, 74%, dr 1/1 at the tertiary alcohol TMS silyl ether) as a yellow oil.

Compound S2 (inseparable mixture of diastereomers, dr 1/1):

R_f = 0.3 (hexane/ethyl acetate = 5/1).

$[\alpha]_D^{23}$ = − 64.5 (c = 0.65 in MeOH).

IR (film) λ_{max}: 2954, 1667, 1435, 1376, 1312, 1250, 1177, 1117, 1097, 1046, 801, 750 cm^{-1}.

^{1}H NMR (600 MHz, $CDCl_3$) δ 6.93 – 6.91 (m, 0.5H), 6.89 – 6.87 (m, 0.5H), 5.40 – 5.38 (m, 1H), 4.79 (s, 0.5H, overlap), 4.78 (s, 0.5H, overlap), 3.93 – 3.79 (m, 4H), 3.35 – 3.26 (m, 2H), 2.99 – 2.89 (m, 2H), 2.58 – 2.54 (m, 1H), 2.46 – 2.45 (m, 0.5H), 2.43 – 2.42 (m, 0.5H) 2.33 – 2.23 (m, 1H), 2.17 – 1.97 (m, 4H), 1.93 – 1.81 (m, 2H), 1.58 – 1.54 (m, 4H, overlap with Me), 1.44 – 1.36 (m, 4H, overlap with Me), 1.12 (m, 1H), 1.27 – 1.17 (m, 2H), 0.719 (s, 3 × 0.5H, overlap), 0.717 (s, 3 × 0.5H, overlap),0.15 – 0.14 (m, 9H).

^{13}C NMR (151 MHz, $CDCl_3$) δ 200.22, 200.21, 139.4, 139.1, 138.4, 137.9, 136.0, 121.8, 107.0, 75.50, 75.47, 65.4, 65.2, 42.0, 41.8, 39.2, 38.58, 38.56, 38.4, 38.3, 27.6, 27.3, 26.7, 24.60, 24.56, 24.5, 24.1, 23.1, 22.8, 22.6, 22.4, 18.9, 18.6, 16.7, 2.6.

HRMS m/z (APCI): calc. for $C_{25}H_{41}O_4ISi^+$ $[M+H]^+$: 561.1892, found: 561.1893.

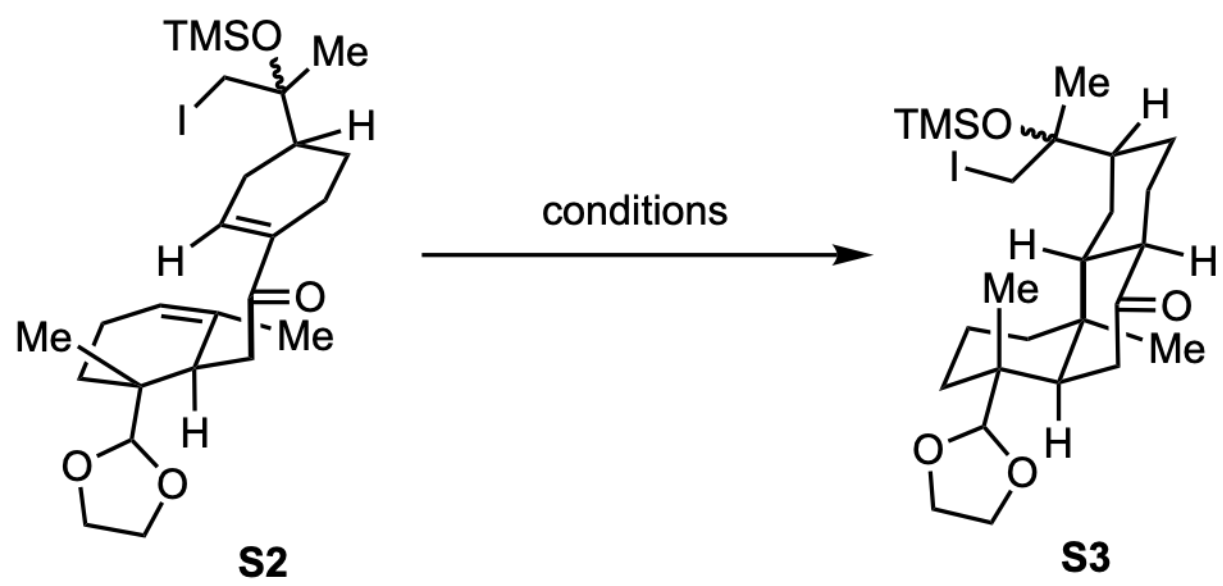

Entry	Conditions	result
1	$Fe(acac)_3$, $PhSiH_3$, EtOH, 60°C, 1 h	**S2** + messy
2	$Fe(acac)_3$, $PhSiH_3$, EtOH, 60°C, 12 h	messy
3	$Fe(acac)_3$, $PhSiH_3$, EtOH, 80°C, 12 h	47% nmr yield
4	$Mn(dpm)_3$, $PhSiH_3$, EtOH, 80°C, 12 h	5% nmr yield
5	$Fe(dibm)_3$, $PhSiH_3$, EtOH, 80°C, 12 h	58% nmr yield
6	$Fe(dpm)_3$, $PhSiH_2$(O*i-Pr*), Na_2HPO_4 EtOH, CF_3Ph, 90°C, 12 h	trace
7	$Fe(dpm)_3$, $PhSiH_3$, EtOH, 80°C, 12 h	62% isolated yield
8	$Fe(dpm)_3$, $PhSiH_3$, EtOH/ethylene glycol , 80°C, 12 h	38% nmr yield
9	$Fe(dpm)_3$, $PhSiH_2$(Oi-Pr), EtOH, 80°C, 12 h	21% nmr yield

Table S1. Condition optimization with S2.

Compound S3 (conditions in Entry 7, Table S1): To a mixture of **S2** (21 mg, 0.037 mmol, 1.0 equiv.) and $Fe(dpm)_3$ (22 mg, 0.037 mmol, 1.0 equiv.) in EtOH (0.74 mL) was added $PhSiH_3$ (14 µL, 0.111 mmol, 3.0 equiv.) at 80 ˚C. The resulting mixture was stirred for 12 h at 80 ˚C. Upon complete consumption of the starting material as indicated by TLC, the reaction mixture was filtered through a short silica gel column and washed with EtOAc. The combined filtrate was concentrated under reduced pressure. The crude residue was purified by flash chromatography (10 ~ 30% ethyl acetate in hexane) to afford product **S3** (13 mg, 62%, dr 1/1 at the tertiary alcohol TMS silyl ether) as a pale-yellow oil.

Compound S3 (inseparable mixture of diastereomers, dr 1/1):

R_f = 0.28 (hexane/ethyl acetate = 5/1).

$[\alpha]_D^{24}$ = −12.3 (*c* = 0.4 in MeOH).

IR (film) λ_{max}: 2929, 1701, 1457, 1373, 1250, 1098, 1039, 983, 911, 753 cm^{-1}.

^{1}H NMR (600 MHz, CDCl$_3$) δ 4.59 (s, 0.5H), 4.58 (s, 0.5H), 3.93 – 3.79 (m, 4H), 3.38 – 3.23 (m, 2H), 2.78 – 2.74 (m, 1H), 2.56 – 2.52 (m, 1H), 2.37 – 2.25 (m, 1H), 2.14 – 2.07 (m, 1H), 1.99 – 1.87 (m, 4H), 1.77 – 1.62 (m, 2H), 1.49 – 1.29 (m, 11H, overlap with Me), 1.12 (s, 6 × 0.5H, overlap with Me), 0.89 (s, 6 × 0.5H, overlap with Me), 0.14 (s, 9H).

^{13}C NMR (151 MHz, CDCl$_3$) δ 214.2, 214.1, 109.9, 109.8, 77.7, 77.3, 65.4, 65.0, 48.6, 48.4, 48.3, 46.8, 41.30, 41.25, 39.9, 39.5, 39.3, 37.8, 37.4, 36.6, 36.5, 36.33, 36.30, 33.1, 33.0, 29.9, 27.0, 26.6, 26.4, 25.7, 24.94, 24.87, 23.9, 22.5, 22.3, 22.2, 19.4, 18.8, 18.6, 17.8, 17.1, 17.0, 2.94, 2.85.

HRMS m/z (ESI): calc. for $C_{22}H_{44}O_4ISi^+$ $[M+H]^+$: 563.2048, found: 563.2039.

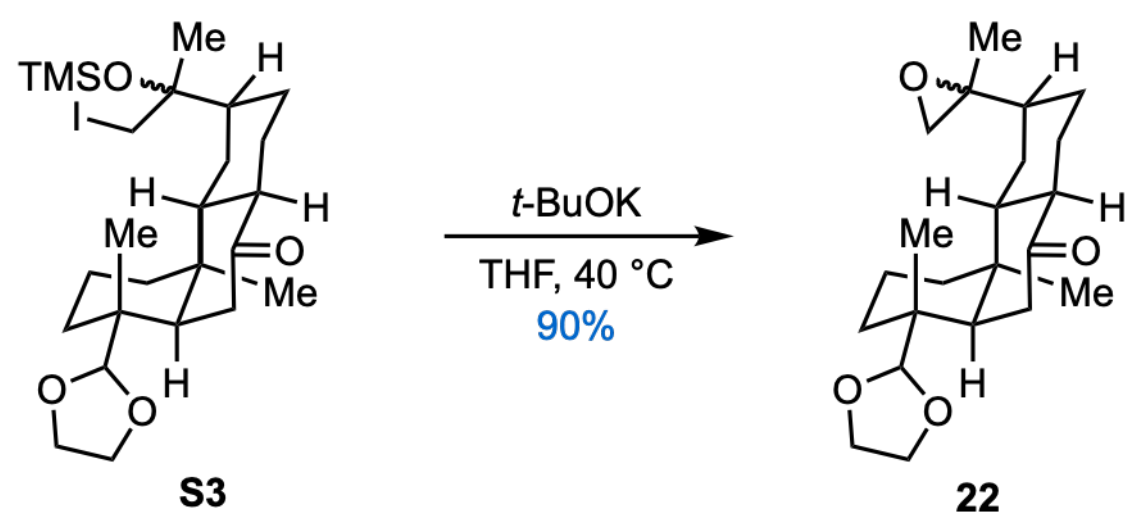

Compound 22. To a solution of **S3** (5 mg, 0.009 mmol, 1.0 equiv.) in THF (0.3 mL) was added *t*-BuOK (3 mg, 0.027 mmol, 3.0 equiv.) at room temperature. The mixture was heated to 40˚C and stirred for 2 h. Upon complete consumption of the starting material as indicated by TLC, the reaction mixture was filtered through a short silica gel column and washed with EtOAc. The combined filtrate was concentrated under reduced pressure. The crude residue was purified by flash chromatography (10 ~ 30% ethyl acetate in hexane) to afford product **22** (3 mg, 90%, dr 1/1 at the epoxide carbon) as a pale-yellow oil. The characterization data for compound **22** are the same as described above.

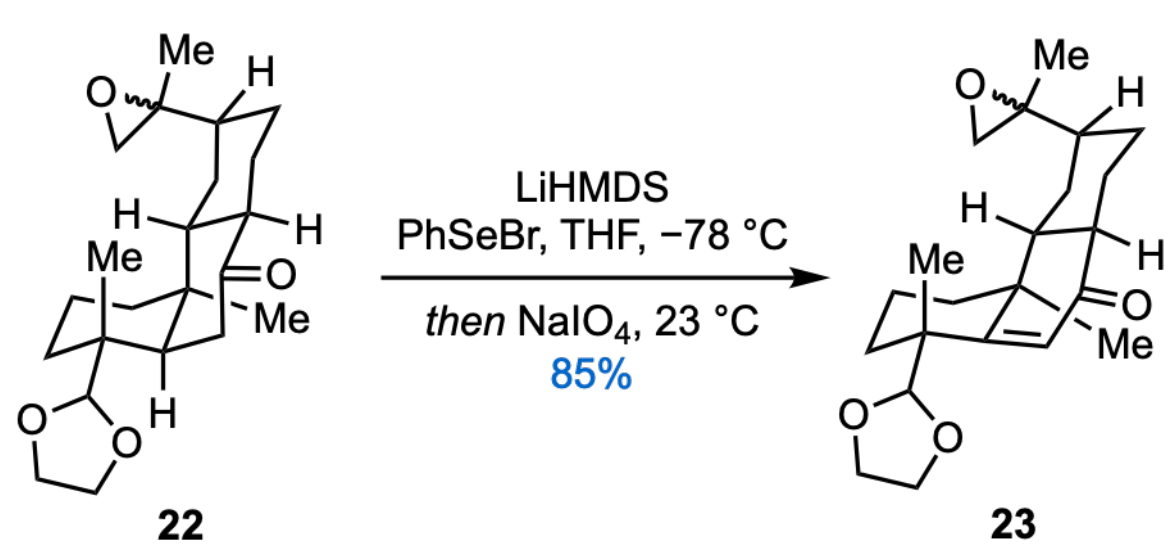

Compound 23. To a solution of **22** (149 mg, 0.4 mmol, 1.0 equiv.) in THF (5 mL) were added dropwise a solution of LiHMDS (1.0 M in THF, 1.6 mL, 1.6 mmol, 4.0 equiv.) at −78 ℃. The reaction mixture was stirred for 30 min at −78 ℃, followed by dropwise addition of a solution of PhSeBr (378 mg, 1.6 mmol, 4.0 equiv.) in THF (3 mL). The resulting mixture was stirred for another 30 min at −78 ℃. Upon full

consumption of the starting material as indicated by TLC, an aqueous solution of $NaIO_4$ (1.37 g, 6.4 mmol, 16.0 equiv.) was added at −78 °C. The resulting mixture was warmed to room temperature and stirred for 2 h. The resultant mixture was quenched carefully with $NaHSO_3$ (10 mL), then extracted with EtOAc (3 × 20 mL). The combined organic phases were washed with brine (80 mL), dried over anhydrous Na_2SO_4, filtered and concentrated under reduced pressure. The crude residue was purified by flash chromatography (10 ~ 30% ethyl acetate in hexane) to afford **23** (122 mg, 85%, dr 1/1 at the epoxide carbon) as a white form.

Note: *PhSeBr should be stored in a glovebox freezer and handled inside the glovebox.*

Compound 23 (inseparable mixture of diastereomers, dr 1/1 at the epoxide carbon):

R*f* = 0.32 (hexane/ethyl acetate = 3/1).

$[\alpha]_D^{24}$ = + 6.0 (*c* = 0.5 in MeOH).

IR (film) λ_{max}: 2934, 2872, 1660, 1454, 1380, 1314, 1218, 1115, 1031, 846, 764 cm^{-1}.

^{1}H NMR (400 MHz, $CDCl_3$) δ 6.02 (s, 1H), 5.034 (s, 0.5H), 5.031 (s, 0.5H), 3.99 – 3.77 (m, 4H), 2.80 (ddd, *J* = 20.5, 4.6, 0.9 Hz, 1H), 2.49 (dd, *J* = 8.6, 4.5 Hz, 1H), 2.25 – 1.98 (m, 4H), 1.99 – 1.73 (m, 5H), 1.55 – 1.35 (m, 4H), 1.33 (s, 3H), 1.30 – 1.17 (m, 2H), 1.15 (s, 3 × 0.5H), 1.13 (s, 3 × 0.5H), 1.09 (s, 3H).

^{13}C NMR (101 MHz, $CDCl_3$) δ 201.24, 201.20, 172.29, 172.28, 127.7, 106.50, 106.47, 65.6, 65.3, 59.0, 58.9, 52.3, 51.9, 46.7, 46.4, 45.2, 45.1, 43.8, 38.7, 38.6, 36.33, 36.28, 35.7, 35.4, 33.5, 26.7, 26.2, 25.9, 25.3, 23.51, 23.49, 23.3, 22.9, 22.3, 22.1, 18.6, 18.5, 17.4.

HRMS m/z (ESI): calc. for $C_{22}H_{33}O_4^+$ $[M+H]^+$: 361.2373, found: 361.2376.

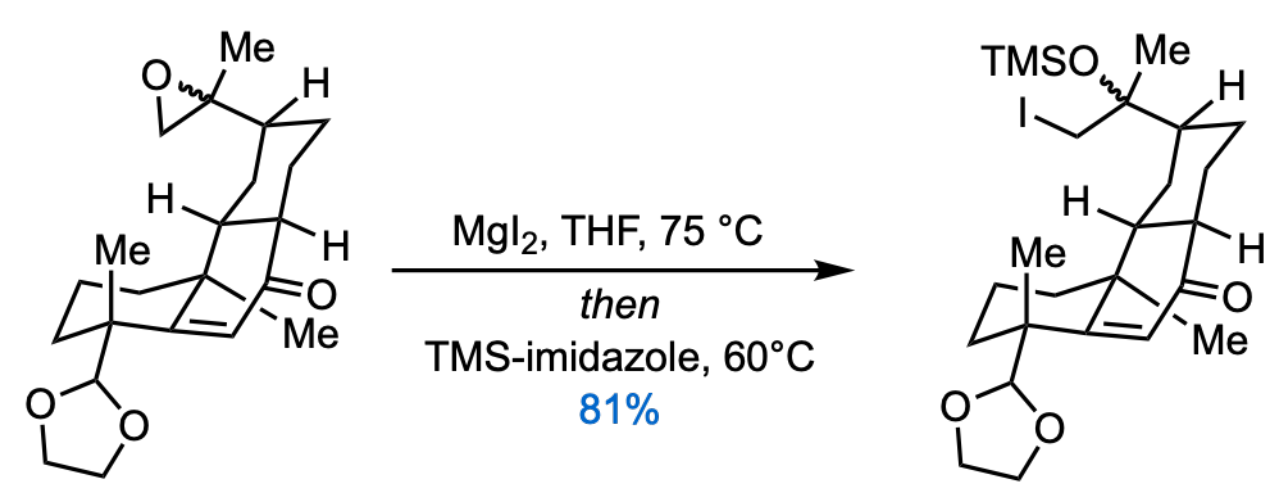

23 24

Compound 24. To a mixture of **23** (107 mg, 0.31 mmol, 1.0 equiv.) and MgI_2 (259 mg, 0.93 mmol, 3.0 equiv.) was added THF (6 mL,) at room temperature. The reaction mixture was heated to 75 °C with an oil bath and stirred for 30 min. Upon full consumption of the starting material as indicated by TLC, the reaction mixture was cooled to 60 °C. Then TMS-imidazole (454 μL, 3.1 mmol, 10.0 equiv.) was added dropwise and the reaction mixture was stirred at this temperature. Upon full consumption of the intermediate as indicated by TLC, the reaction was quenched with saturated aqueous $NaHCO_3$ (10 mL) and extracted with EtOAc (3 × 15 mL). The combined organic phases were dried over anhydrous Na_2SO_4, filtered and concentrated under reduced pressure. The crude residue was purified by flash column chromatography on

silica gel (10 ~ 20% ethyl acetate in hexane) to afford **24** (141 mg, 81%, dr 1/1 at the tertiary TMS silyl ether carbon) as a yellow oil.

Compound 24 (inseparable mixture of diastereomers, dr 1/1 at the tertiary TMS silyl ether carbon):

R_f = 0.3 (hexane/ethyl acetate = 5/1).

$[\alpha]_D^{24}$ = − 6.5 (*c* = 0.2 in MeOH).

IR (film) λ_{max}: 2929, 1663, 1456, 1374, 1250, 1109, 1047, 983, 840, 755 cm^{-1}.

^{1}H NMR (600 MHz, $CDCl_3$) δ 6.014 (s, 0.5H), 6.011 (s, 0.5H), 5.03 (s, 0.5H), 5.02 (s, 0.5H), 3.97 – 3.79 (m, 4H), 3.39 – 3.29 (m, 1H), 3.24 – 3.20 (m, 1H), 2.24 – 2.13 (m, 2H), 2.07 – 1.99 (m, 2H), 1.92 – 1.73 (m, 4H), 1.73 – 1.65 (m, 1H), 1.63 – 1.56 (m, 1H), 1.52 – 1.44 (m, 1H), 1.44 (s, 3 × 0.5H), 1.43 (s, 3 × 0.5H), 1.32 (m, 2H), 1.27 – 1.17 (m, 2H), 1.15 (s, 3 × 0.5H), 1.14 (s, 3 × 0.5H), 1.09 (s, 3 × 0.5H), 1.08 (s, 3 × 0.5H), 0.10 (s, 9H).

^{13}C NMR (151 MHz, $CDCl_3$) δ 202.3, 202.2, 172.8, 172.7, 127.6, 127.5, 106.7, 106.6, 77.9, 77.8, 65.7, 65.4, 45.7, 45.3, 44.22, 44.18, 43.9, 43.8, 39.0, 38.94, 38.85, 38.6, 36.5, 36.4, 33.64, 33.62, 26.7, 26.2, 25.1, 24.8, 24.2, 24.0, 23.83, 23.75, 22.7, 22.4, 18.4, 18.2, 17.9, 17.8, 17.5, 2.84, 2.75.

HRMS m/z (APCI): calc. for $C_{25}H_{42}O_4ISi^+$ $[M+H]^+$: 561.1892, found: 561.1895.

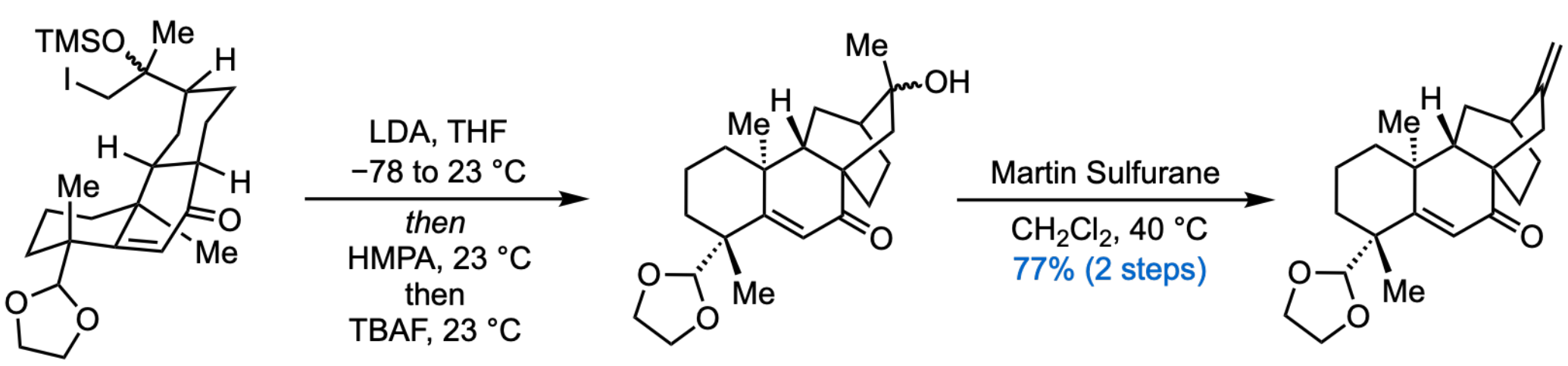

24 25 26

Compound 26. To a solution of **24** (64 mg, 0.114 mmol, 1.0 equiv.) in THF (2.3 mL) was added a solution of LDA (1.0 M in THF, 285 μL, 0.285 mmol, 2.5 equiv.) at −78 °C. The reaction mixture was stirred for 10 min at −78 °C, then warmed to room temperature and stirred for an additional 10 min before HMPA (96 μL, 0.57 mmol, 5.0 equiv.) was added. The reaction mixture was stirred for 6 h before a solution of TBAF (1.0 M in THF, 228 μL, 0.228 mmol, 2.0 equiv.) was added. The resulting mixture was stirred for an additional 10 min and then quenched with saturated aqueous $NaHCO_3$ (20 mL), extracted with EtOAc (3 × 30 mL). The combined organic phases were washed with brine (30 mL), dried over anhydrous Na_2SO_4, filtered and concentrated under reduced pressure. The resulting residue was filtered through a short silica gel column and washed with 50% ethyl acetate in hexane. The combined filtrate was concentrated under

reduced pressure to afford crude product **25**, which was used directly subjected to the next step without further purification.

Note: *The starting material for this reaction must be of high purity; otherwise, product formation is significantly hindered.*

To the above crude product and Martin Sulfurane dehydrating reagent (153 mg, 0.228 mmol, 2.0 equiv.) was added DCM (2.3 mL) at room temperature. The reaction mixture was moved to a 40 °C oil bath and stirred for 30 min. Upon full consumption of the starting material as indicated by TLC. The resultant mixture was purified directly by flash column chromatography on silica gel (10 ~ 30% ethyl acetate in hexane) to afford **26** (30 mg, 77% for 2 steps) as a white foam.

Compound 26:

R_f = 0.35 (hexane/ethyl acetate = 3/1).

$[\alpha]_D^{24}$ = + 8.4 (*c* = 0.2 in MeOH).

IR (film) λ_{max}: 2924, 2868, 1655, 1455, 1376, 1309, 1159,1112, 1075, 879 cm^{-1}.

^{1}H NMR (400 MHz, $CDCl_3$) δ 6.03 (s, 1H), 5.05 (s, 1H), 4.82 (q, *J* = 2.1 Hz, 1H), 4.67 (q, *J* = 2.0 Hz, 1H), 4.00 – 3.77 (m, 4H), 2.69 – 2.60 (m, 1H), 2.39 – 2.35 (m, 1H), 2.14 – 1.88 (m, 4H), 1.86 – 1.65 (m, 5H), 1.53 – 1.46 (m, 2H), 1.31 (ddd, *J* = 14.1, 12.6, 4.5 Hz, 2H), 1.26 (s, 3H), 1.18 (td, *J* = 13.2, 4.5 Hz, 1H), 1.08 (s, 3H).

^{13}C NMR (101 MHz, $CDCl_3$) δ 204.2, 173.3, 150.6, 127.4, 106.7, 106.2, 77.4, 65.7, 65.4, 43.8, 43.5, 42.1, 39.0, 36.8, 36.4, 34.4, 28.9, 26.5, 25.9, 23.1, 21.0, 17.0.

HRMS m/z (ESI): calc. for $C_{22}H_{31}O_3^+$ $[M+H]^+$: 343.2268, found: 343.2261.

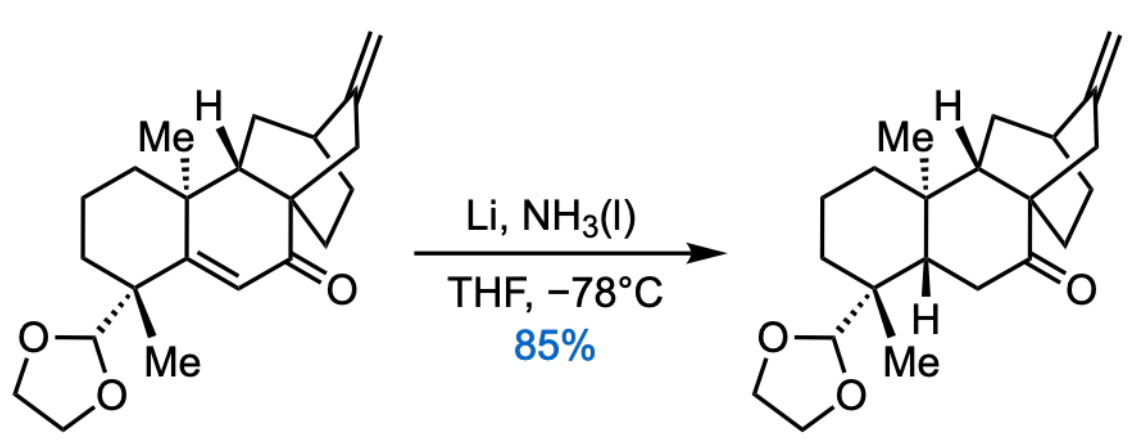

26 **27**

Compound 27. Lithium (20 mg, 2.92 mmol, 40.0 equiv.) was added to a 10 mL round-bottom flask under an argon atmosphere. The flask was then cooled to −78 °C. The argon balloon was then replaced with an NH_3 balloon, while a needle was inserted through the rubber septum to vent the system. Approximately 3-5 mL of NH_3 was then condensed into the flask. A solution of compound **26** (25 mg, 0.073 mmol, 1.0 equiv.) in THF (3.7 mL) was added dropwise at −78 °C. The resulting mixture was stirred for 1 h before solid NH_4Cl was added until the blue color disappeared. The resultant mixture was quenched with saturated

aqueous NH_4Cl (3 mL) and extracted with EtOAc (3 × 10 mL). The combined organic phases were dried over anhydrous Na_2SO_4, filtered and concentrated under reduced pressure. The crude residue was purified by flash column chromatography on silica gel (10 ~ 30% ethyl acetate in hexane) to afford **27** (21.3 mg, 85%) as a white foam.

Compound 27:

R_f = 0.45 (hexane/ethyl acetate = 3/1).

$[\alpha]_D^{24}$ = − 13.4 (c = 0.1 in MeOH).

IR (film) λ_{max}: 2924, 2850, 1653, 1636, 1097, 1076, 990, 885 cm^{-1}.

^{1}H NMR (600 MHz, $CDCl_3$) δ 5.20 (s, 1H), 4.78 (q, J = 2.2 Hz, 1H), 4.66 (q, J = 2.0 Hz, 1H), 4.01 – 3.73 (m, 4H), 2.80 (dd, J = 16.1, 14.7 Hz, 1H), 2.65 (dt, J = 17.3, 2.6 Hz, 1H), 2.52 – 2.46 (m, 1H), 2.33 – 2.28 (m, 1H), 2.09 (ddt, J = 17.3, 3.9, 2.1 Hz, 1H), 2.04 – 1.91 (m, 2H), 1.73 – 1.61 (m, 5H), 1.54 – 1.34 (m, 4H), 1.34 – 1.23 (m, 1H), 1.18 (s, 3H), 1.08 (td, J = 13.9, 4.0 Hz, 1H), 0.93 (td, J = 12.5, 4.4 Hz, 1H), 0.84 (s, 3H).

^{13}C NMR (151 MHz, $CDCl_3$) δ 215.8, 150.5, 106.2, 105.5, 66.0, 64.2, 54.5, 53.0, 47.5, 40.4, 40.0, 39.3, 38.6, 37.7, 37.6, 36.0, 28.7, 27.6, 26.2, 21.6, 17.7, 14.2.

HRMS m/z (APCI): calc. for $C_{22}H_{31}O_3^+$ $[M+H]^+$: 345.2424, found: 345.2427.

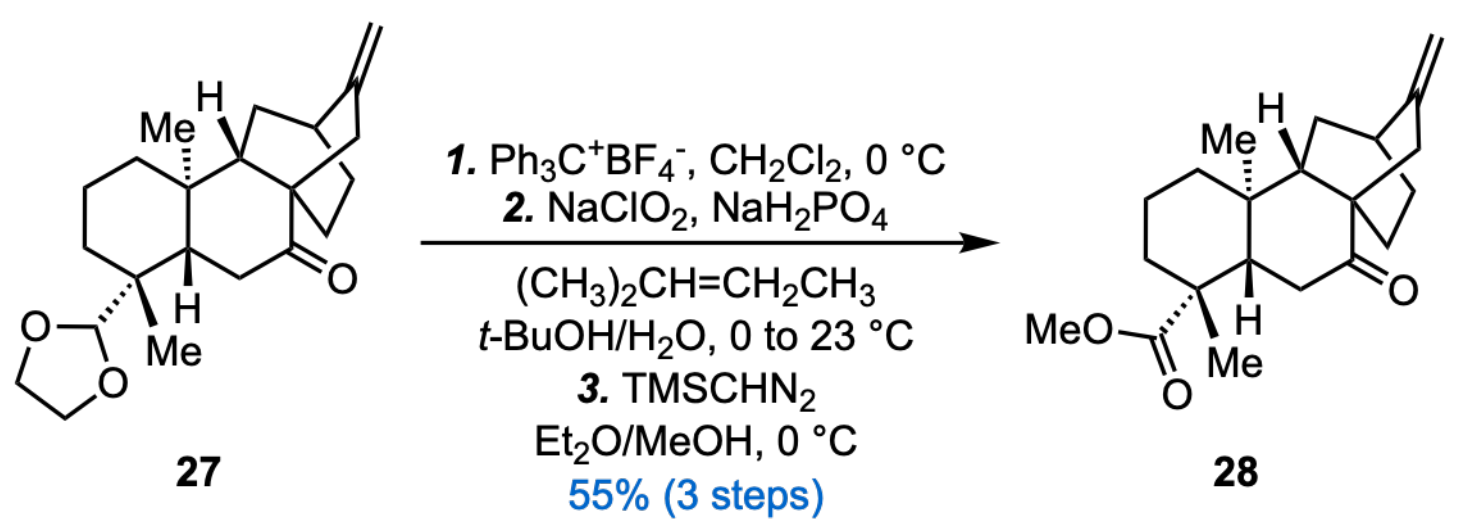

Compound 28. To a solution of **27** (17 mg, 0.049 mmol, 1.0 equiv.) in DCM (2.5 mL) was added $Ph_3C^+BF_4^-$ (32.6 mg, 0.098 mmol, 2.0 equiv.) at 0 °C. The resulting mixture was stirred for 30 min at 0 °C. Upon full consumption of the starting material as indicated by TLC, the resultant mixture was quenched with $NaHCO_3$ (5 mL) and extracted with DCM (3 × 10 mL). The combined organic phases were washed with brine (20 mL), dried over anhydrous Na_2SO_4, filtered and concentrated under reduced pressure. The resulting residue was filtered through a short silica gel column and washed with 20% ethyl acetate in hexane. The combined filtrate was concentrated under reduced pressure to afford the crude product which was directly proceeded to the next step without further purification and.

To a solution of the above crude product in t-BuOH/H_2O (1.2 mL/0.6 mL) was added 2-methyl-2-buetene (111 μL, 0.98 mmol, 20.0 equiv.), NaH_2PO_4 (41 mg, 0.294 mmol, 6.0 equiv.), and $NaClO_2$ (22 mg, 0.245

mmol, 5.0 equiv.) at 0 °C. The resulting mixture was warmed up to room temperature and stirred for 20 min before it was quenched with H_2O (5 mL) and extracted with EtOAc (3 × 10 mL). The combined organic phases were washed with brine (20 mL), dried over anhydrous Na_2SO_4, filtered and concentrated under reduced pressure. The crude acid was used for the next step without further purification.

To the crude acid obtained above in Et_2O/MeOH (1 mL/0.3 mL) was added $TMSCHN_2$ (10% in hexane) dropwise at 0 °C until the yellowish color remained. The mixture was stirred for another 30 min. The solvent was removed under vacuum. The crude product was purified by flash column chromatography (10 ~ 25% ethyl acetate in hexane) to afford **28** (8.9 mg, 55% for 3 steps) as a white foam.

Compound 28:

R_f = 0.45 (hexane/ethyl acetate = 5/1).

$[\alpha]_D^{24}$ = − 31.0 (*c* = 0.1 in $CHCl_3$).

IR (film) λ_{max}: 2924, 2851, 1730, 1648, 1435, 1373, 1258, 885, 872 cm^{-1}.

^{1}H NMR (600 MHz, $CDCl_3$) δ 4.79 (q, *J* = 2.4 Hz, 1H), 4.68 (q, *J* = 2.0 Hz, 1H), 3.68 (s, 3H), 3.00 (dd, *J* = 15.7, 14.4 Hz, 1H), 2.68 – 2.59 (m, 2H), 2.32 (dq, *J* = 5.3, 3.0 Hz, 1H), 2.25 – 2.21 (m, 1H), 2.13 – 2.08 (m, 1H), 2.08 – 1.99 (m, 1H), 1.89 – 1.79 (m, 1H), 1.72 – 1.61 (m, 4H), 1.54 – 1.42 (m, 4H), 1.37 – 1.29 (m, 1H), 1.17 (s, 3H), 1.03 (td, *J* = 13.6, 4.1 Hz, 1H), 0.97 (d, *J* = 0.9 Hz, 3H), 0.92 (td, *J* = 13.4, 4.3 Hz, 1H).

^{13}C NMR (151 MHz, $CDCl_3$) δ 215.4, 177.3, 150.2, 106.5, 54.2, 52.0, 51.6, 47.7, 44.0, 40.0, 39.3, 38.5, 38.0, 37.9, 36.0, 28.5, 28.3, 27.8, 26.2, 18.8, 11.9.

HRMS m/z (ESI): calc. for $C_{21}H_{31}O_3^+$ $[M+H]^+$: 331.2268, found: 331.2266.

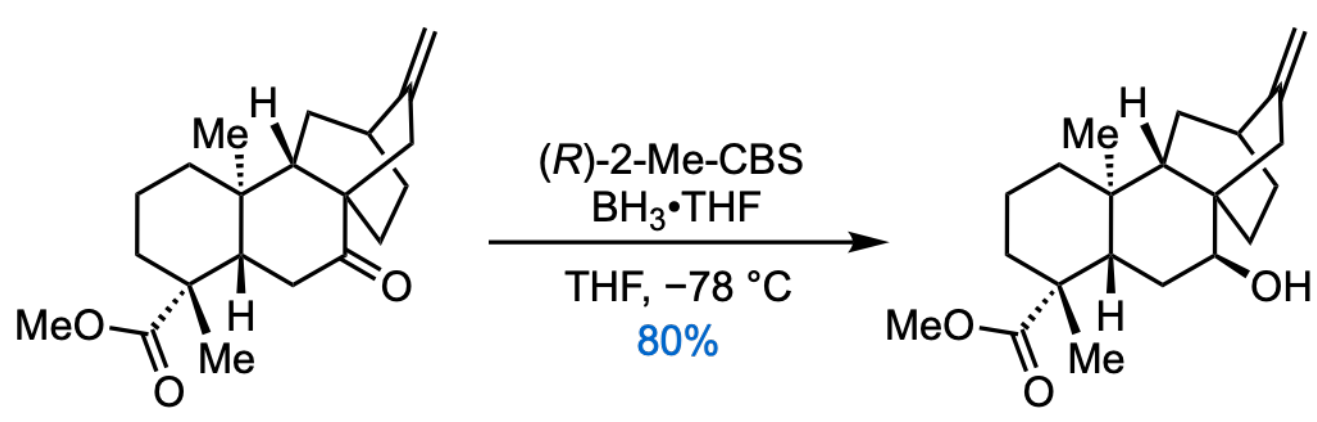

Compound 29. To a solution of (*R*)-2-Me-CBS (14 mg, 0.05 mmol, 3.0 equiv.) in THF (0.5 mL) was added a solution of $BH_3 \cdot THF$ (1.0 M in THF, 50 μL, 0.05 mmol, 3.0 equiv.) at room temperature. The resulting mixture was stirred for 10 min before a solution of **28** (5.6 mg, 0.017 mmol, 1.0 equiv.) in THF (0.5 mL) was added dropwise at −78 °C. The reaction mixture was stirred for 10 min at −78 °C. Upon full consumption of the starting material as indicated by TLC, the resultant mixture was quenched with saturated aqueous $NaHCO_3$ (5 mL) and extracted with DCM (3 × 10 mL). The combined organic phases were dried

over anhydrous Na_2SO_4, filtered and concentrated under reduced pressure. The crude residue was purified by flash column chromatography on silica gel (10 ~ 30% ethyl acetate in hexane) to afford **29** (4.5 mg, 80%) as a white foam.

Compound 29:

R_f = 0.35 (hexane/ethyl acetate = 5/1).

$[\alpha]_D^{22}$ = −14.9 (c = 0.1 in EtOH).

IR (film) λ_{max}: 2923, 2853, 1732, 1667, 1456, 1377, 1258, 833 cm^{-1}.

^{1}H NMR (600 MHz, $CDCl_3$) δ 4.76 (q, J = 2.3 Hz, 1H), 4.63 (q, J = 2.1 Hz, 1H), 3.65 (s, 3H), 3.51 (q, J = 3.1 Hz, 1H), 2.41 (dq, J = 16.6, 2.9 Hz, 1H), 2.27 – 2.22 (m, 1H), 2.22 – 2.16 (m, 1H), 2.12 (ddd, J = 14.8, 13.2, 2.5 Hz, 1H), 1.98 – 1.88 (m, 2H), 1.86 – 1.74 (m, 2H), 1.67 – 1.54 (m, 6H), 1.46 – 1.38 (m, 2H), 1.31 (d, J = 3.7 Hz, 1H), 1.18 (s, 3H), 1.10 – 1.05 (m, 2H), 0.92 (td, J = 13.4, 4.3 Hz, 1H), 0.79 (s, 3H).

^{13}C NMR (151 MHz, $CDCl_3$) δ 178.2, 152.1, 105.3, 73.8, 51.4, 48.2, 46.4, 43.5, 42.0, 39.6, 38.22, 38.18, 37.7, 36.5, 28.7, 28.4 (overlap), 27.7, 27.2, 18.9, 11.6.

HRMS m/z (APCI): calc. for $C_{21}H_{33}O_3^+$ $[M+H]^+$: 333.2424, found: 333.2425.

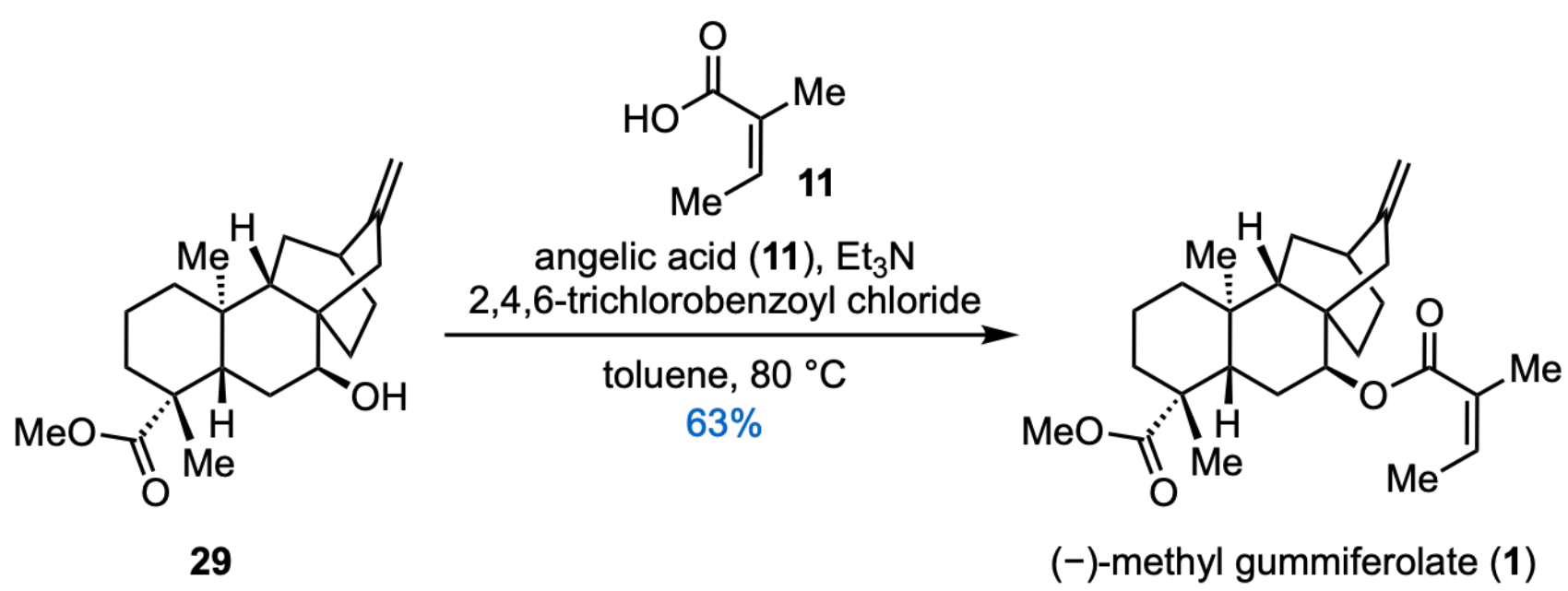

(-)-Methyl gummiferolate (1). To a solution of angelic acid (20 mg, 0.20 mmol, 40.0 equiv.) in toluene (0.5 mL) was added Et_3N (28 μL, 0.20 mmol, 40.0 equiv.) and trichlorobenzoyl chloride (31 μL, 0.20 mmol, 40.0 equiv.) at room temperature. The resulting mixture was stirred at room temperature for 2 h, then transferred to the reaction flask containing **29** (1.7 mg, 0.005 mmol, 1.0 equiv.). The reaction mixture was warmed up to 80 °C and stirred for 24 min. Upon full consumption of the starting material as indicated by TLC, the resultant mixture was purified directly by flash column chromatography on silica gel (8 ~ 20% ethyl acetate in hexane) to afford **1** (1.3 mg, 63%) as a colorless oil [3].

(-)-Methyl gummiferolate:

R_f = 0.55 (hexane/ethyl acetate = 5/1).

$[\alpha]_D^{24}$ = −26.0 (c = 0.2 in $CHCl_3$).

IR (film) λ_{max}: 2925, 2869, 1727, 1716, 1648, 1454, 1378, 1232, 1157, 1037, 874 cm^{-1}.

^{1}H NMR (600 MHz, $CDCl_3$) δ 6.05 (qq, *J* = 7.2, 1.5 Hz, 1H), 4.84 (t, *J* = 3.0 Hz, 1H), 4.74 (q, *J* = 2.3 Hz, 1H), 4.59 (q, *J* = 2.1 Hz, 1H), 3.65 (s, 3H), 2.28 – 2.05 (m, 4H), 2.02 (dq, *J* = 7.3, 1.6 Hz, 3H), 1.95– 1.92 (m, 3H), 1.92 – 1.72 (m, 3H), 1.67 – 1.56 (m, 4H), 1.46 – 1.39 (m, 2H), 1.29 – 1.13 (m, 2H), 1.09 (s, 3H), 1.09 – 0.82 (m, 4H), 0.82 (s, 3H).

^{13}C NMR (151 MHz, $CDCl_3$) δ 177.9, 167.4, 151.7, 137.3, 128.6, 105.4, 75.9, 51.4, 49.6, 47.7, 43.4, 41.8, 39.8, 38.4, 38.2, 37.0, 36.4, 28.6, 28.0, 27.8, 27.0, 25.7, 21.1, 18.9, 16.0, 11.7.

HRMS m/z (ESI): calc. for $C_{26}H_{38}O_4Na^+$ $[M+Na]^+$: 437.2662, found: 437.2662

Part 2. Natural Product NMR Data Comparison

Table S2. ^{1}H NMR (CD_3Cl) Comparison of Natural & Our Synthetic (-)-Methyl Gummiferolate.

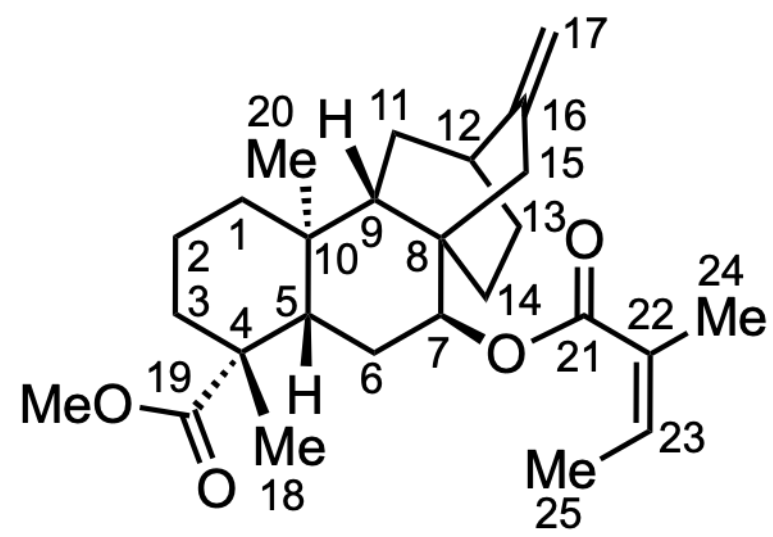

(−)-methyl gummiferolate (**1**)

No.	Natural Sample 100 MHz[4] δ (ppm, *J* in Hz)	Toyota and Ihara's Synthetic Sample 300 MHz[3] δ (ppm, *J* in Hz)	Our Synthetic Sample 600 MHz δ (ppm, *J* in Hz)	Error (Syn. – Nat.)
23	6.03, qq (7.0, 1.0)	6.06, qq (7.0, 1.5)	6.05 qq (7.2, 1.5)	0.02
7	4.83, t (3.0)	4.85, t (3.0)	4.84, t (3.0)	0.01
17	4.73, q (2.0)	4.75, q (2.0)	4.74, q (2.3)	0.01
17	4.57, q, (2.0)	4.59, q, (2.0)	4.59, q (2.1)	0.02
OMe	3.64, s	3.65, s	3.65, s	0.01
–	–	2.28 – 2.05, m	2.28 – 2.05, m	–
25	2.0, dq (7.0, 1.0)	2.02, dq (7.0, 1.0)	2.02, dq (7.3, 1.6)	0.02
24	1.93, q (1.0)	1.95– 1.92, m	1.95– 1.92, m	–
–	–	1.92 – 1.72, m	1.92 – 1.72, m	–
–	–	1.67 – 1.13, m	1.67 – 1.56, m<br>1.46 – 1.39, m<br>1.29 – 1.13, m	
18	1.08, s	1.09, s	1.09, s	0.01
–	–	1.09 – 0.82, m	1.09 – 0.82, m	–
20	0.82, s	0.82, s	0.82, s	0

Table S3. ^{13}C NMR (CD_3Cl) Comparison of Natural & Our Synthetic (-)-Methyl Gummiferolate.

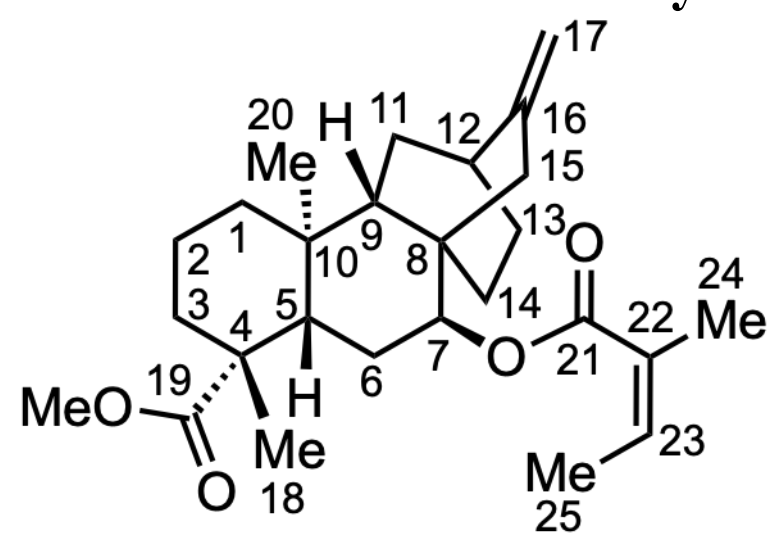

(−)-methyl gummiferolate (**1**)

No.	Natural Sample 25.2 MHz[4] δ (ppm)	Our Synthetic Sample, 151 MHz δ (ppm)	Error (Syn. – Nat.)
1	39.7, CH_2	39.8	0.1
2	18.7, CH_2	18.9	0.2
3	38.2, CH_2	38.4	0.2
4	43.3, C	43.4	0.1
5	49.4, CH	49.6	0.2
6	27.9, CH_2	28.0	0.1
7	75.7, CH	75.9	0.2
8	36.8, C	37.0	0.2
9	47.6, CH	47.7	0.1
10	38.0, C	38.2	0.2
11	27.7, CH_2	27.8	0.1
12	36.3, CH	36.4	0.1
13	25.5, CH_2	25.7	0.2
14	26.9. CH_2	27.0	0.1
15	41.7, CH_2	41.8	0.1
16	151.1, C	151.7	0.6
17	105.1, CH_2	105.4	0.3
18	28.5, CH_3	28.6	0.1
19	177.4, C	177.9	0.5

20	11.6, CH_3	11.7	0.1
OMe	51.1, CH_3	51.4	0.3
21	166.9, C	167.4	0.5
22	128.3, C	128.6	0.3
23	136.8, CH	137.3	0.5
24	20.9, CH_3	21.1	0.2
25	15.8, CH_3	16.0	0.2

Part 3. References

[1] (a) Kozmin, S. A.; Janey, J. M.; Rawal, V. H. 1-Amino-3-siloxy-1,3-butadienes: Highly Reactive Dienes for the Diels-Alder Reaction. *J. Org. Chem*. **1999**, *64*, 3039−3052. (b) Jin, S.; Zhao, X.; Ma, D. Divergent Total Syntheses of Napelline-type C20-Diterpenoid Alkaloids: (−)-Napelline, (+)-Dehydronapelline, (−)-Songorine, (−)-Songoramine, (−)-Acoapetaldine D, and (−)-Liangshanone. *J. Am. Chem. Soc*. **2022**, *144*, 15355−15362.

[2] Syeda, S. S.; Carlson, E. J.; Miller, M. R.; Francis, R.; Clapham, D. E.; Lishko, P. V.; Hawkinson, J. E.; Hook, D.; Georg, G. I. The fungal sexual pheromone sirenin activates the human CatSper channel complex, *ACS Chem. Biol*. **2016**, *11*, 452–459.

[3] Toyota, M.; Yokota, M.; Ihara, M. Remarkable control of radical cyclization processes of cyclic enyne: Total syntheses of (±)-methyl gummiferolate, (±)-methyl 7β-hydroxykaurenoate, and (±)-methyl 7-oxokaurenoate and formal synthesis of (±)-gibberellin A12 from a common synthetic precursor. *J. Am. Chem. Soc*. **2001**, *123*, 1856−1861.

[4] Pinar, M.; Rodriguez, B.; Alemany, A. Gummiferolic acid, a new *ent*-atis-16-ene diterpenoid from Margotia gummifera. *Phytochemistry* **1978**, *17*, 1637−1640.

Part 4. ^{1}H and ^{13}C NMR Spectra

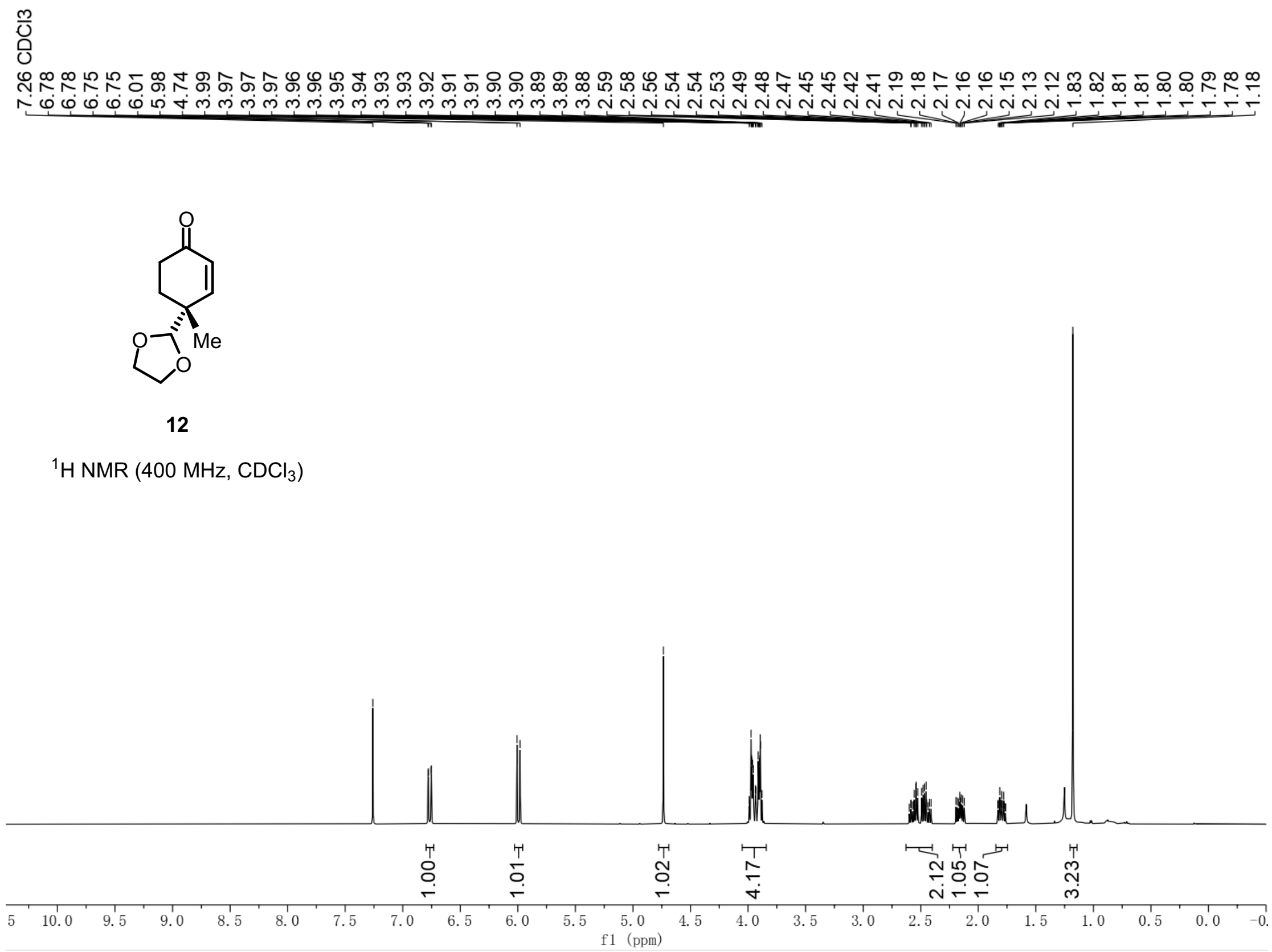

12

^{1}H NMR (400 MHz, $CDCl_3$)

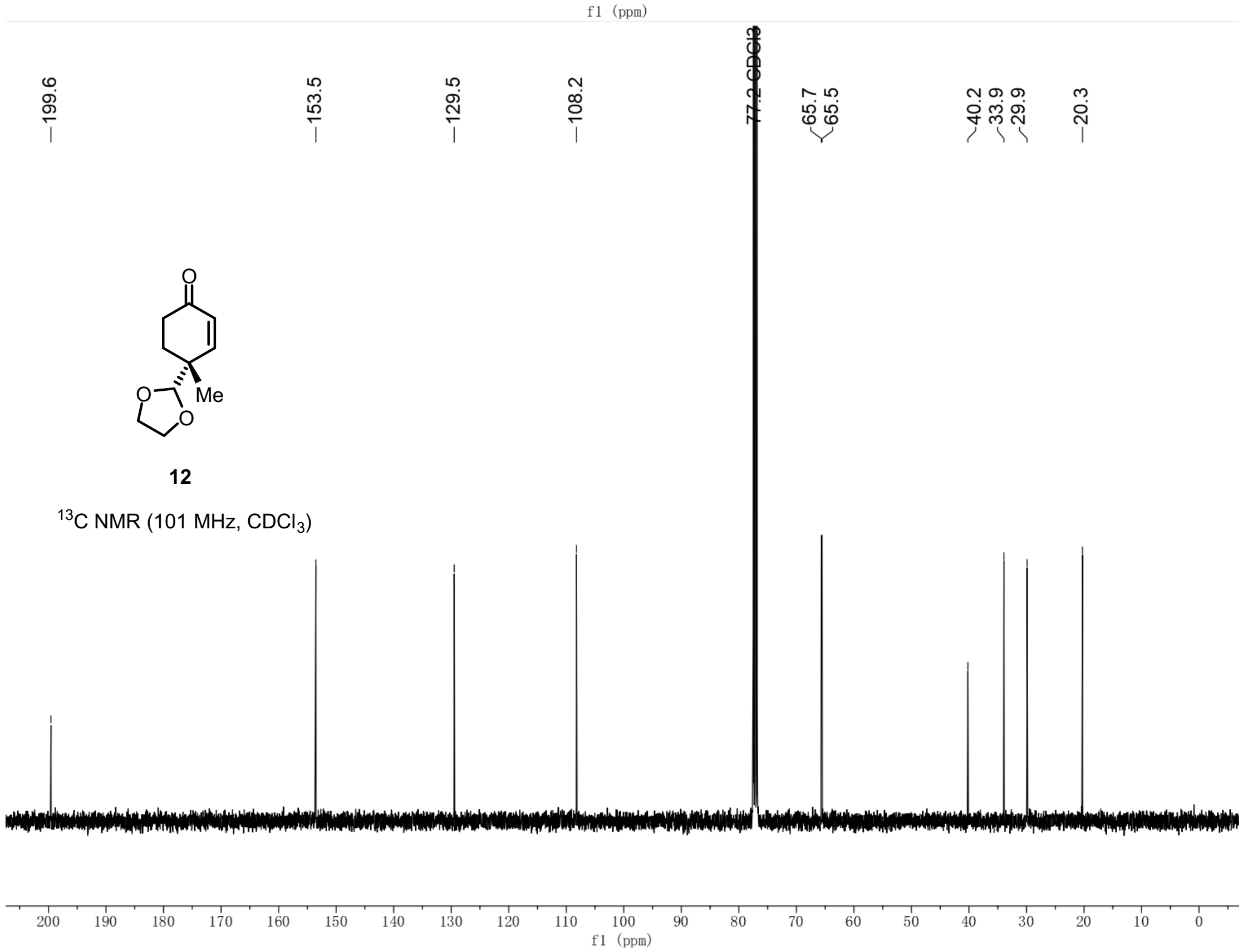

12

^{13}C NMR (101 MHz, $CDCl_3$)

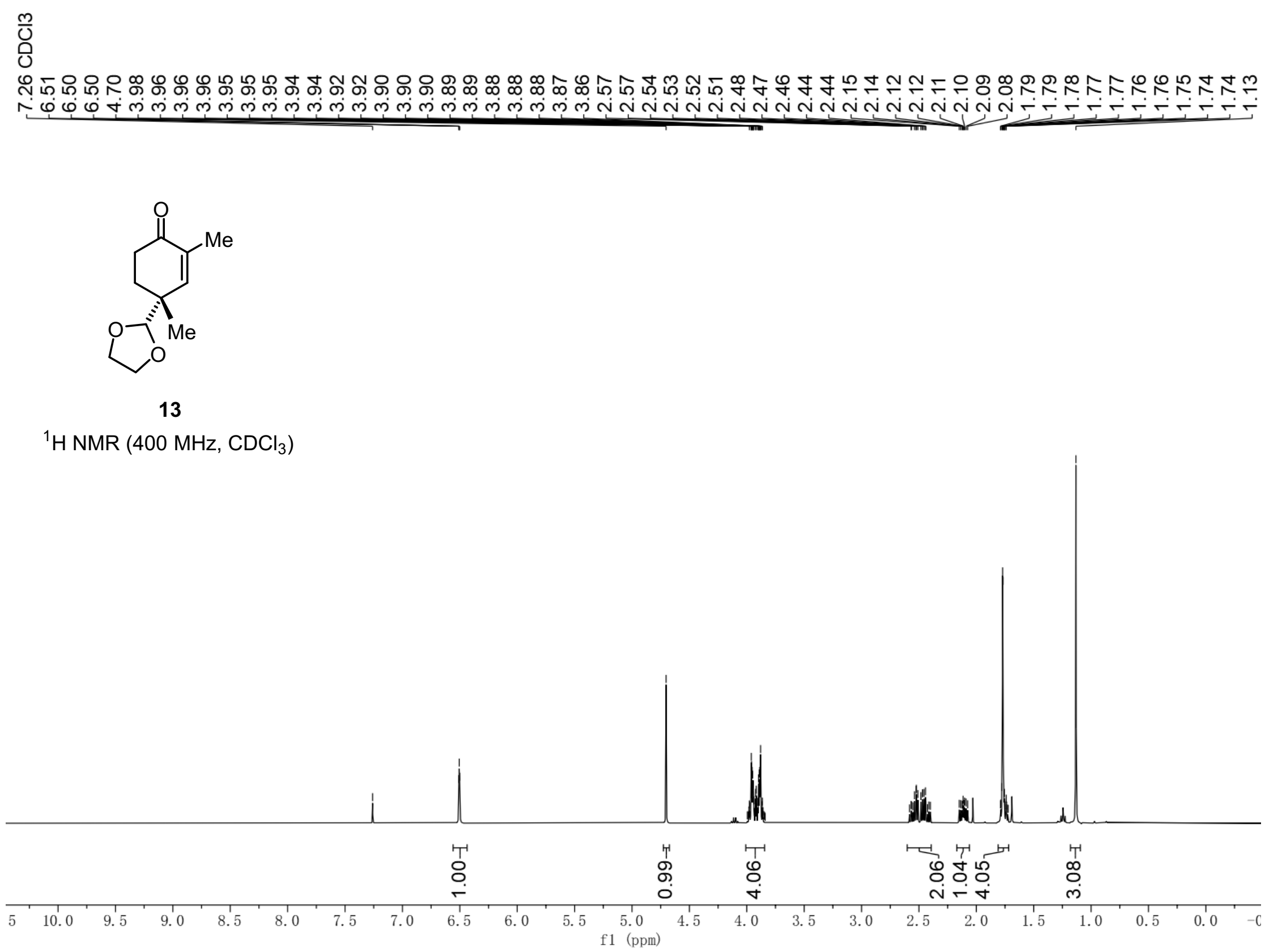

13

^{1}H NMR (400 MHz, $CDCl_3$)

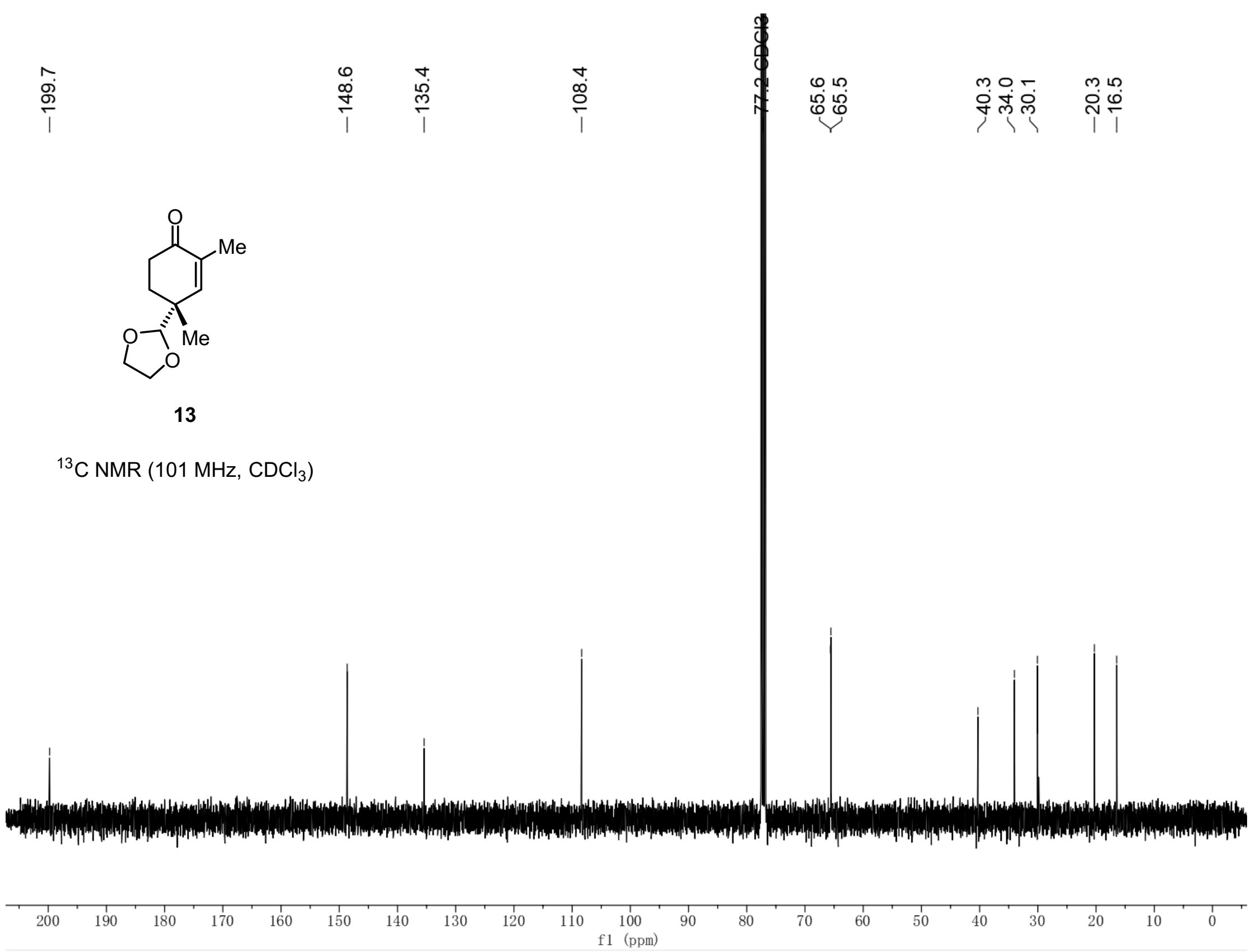

13

^{13}C NMR (101 MHz, $CDCl_3$)

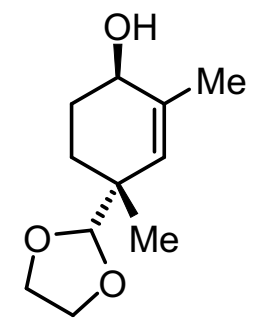

14

^{1}H NMR (600 MHz, $CDCl_3$)

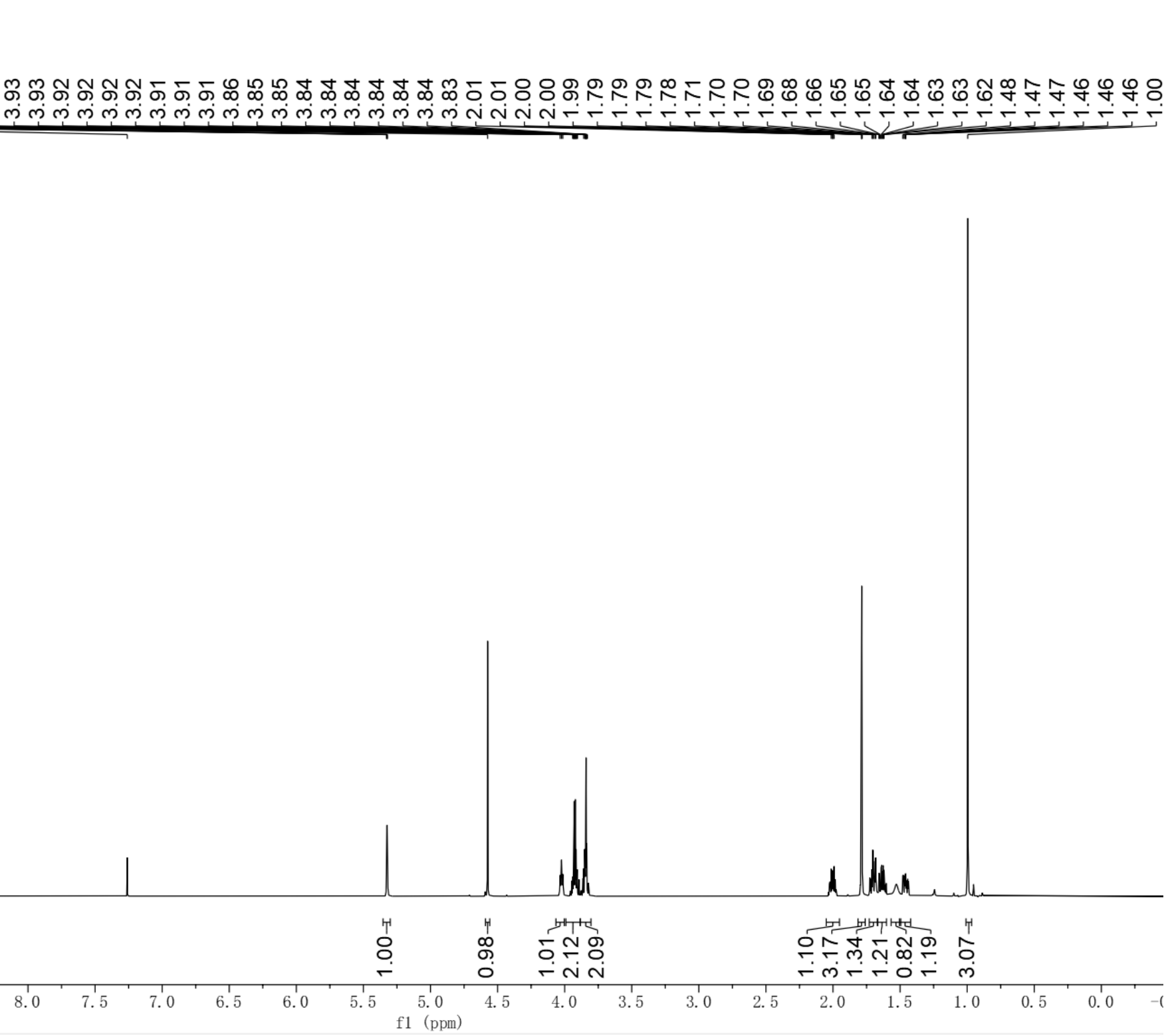

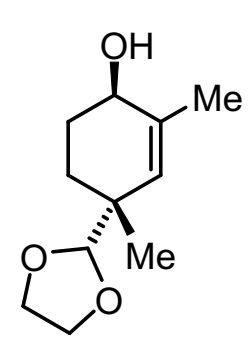

14

^{13}C NMR (151 MHz, $CDCl_3$)

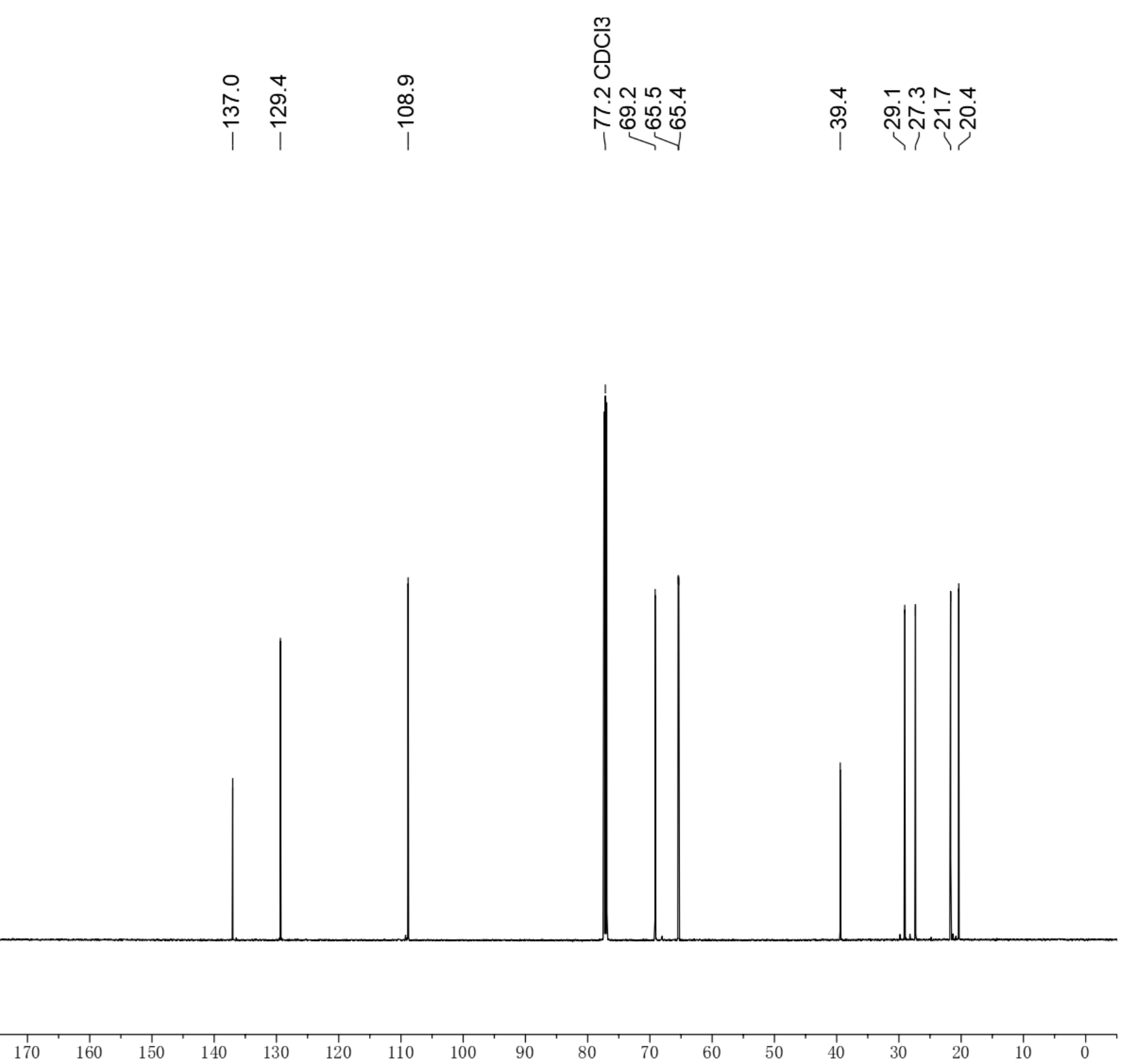

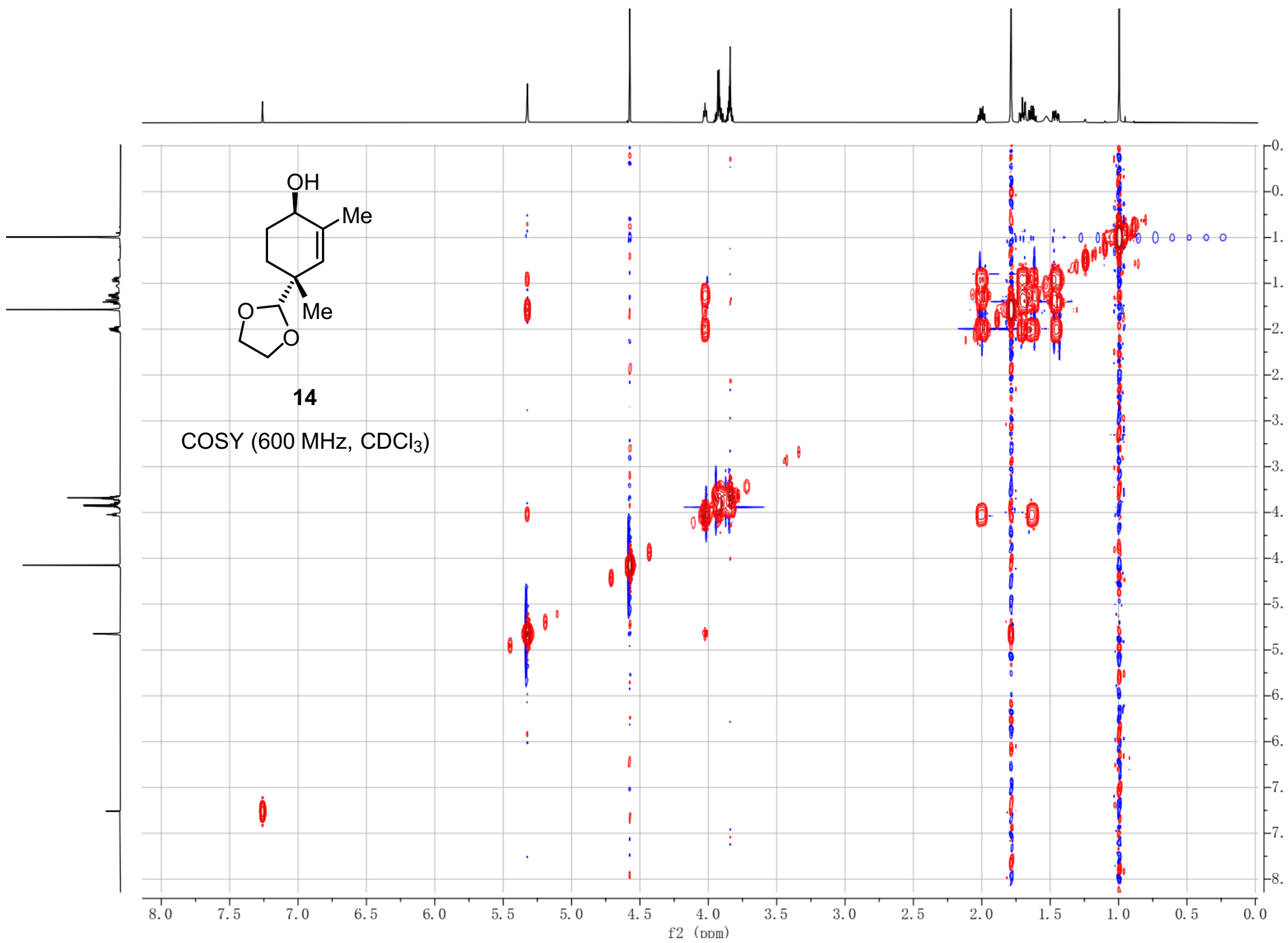
OH
Me
O
O
Me
14
COSY (600 MHz, CDCl3)
f2 (ppm)

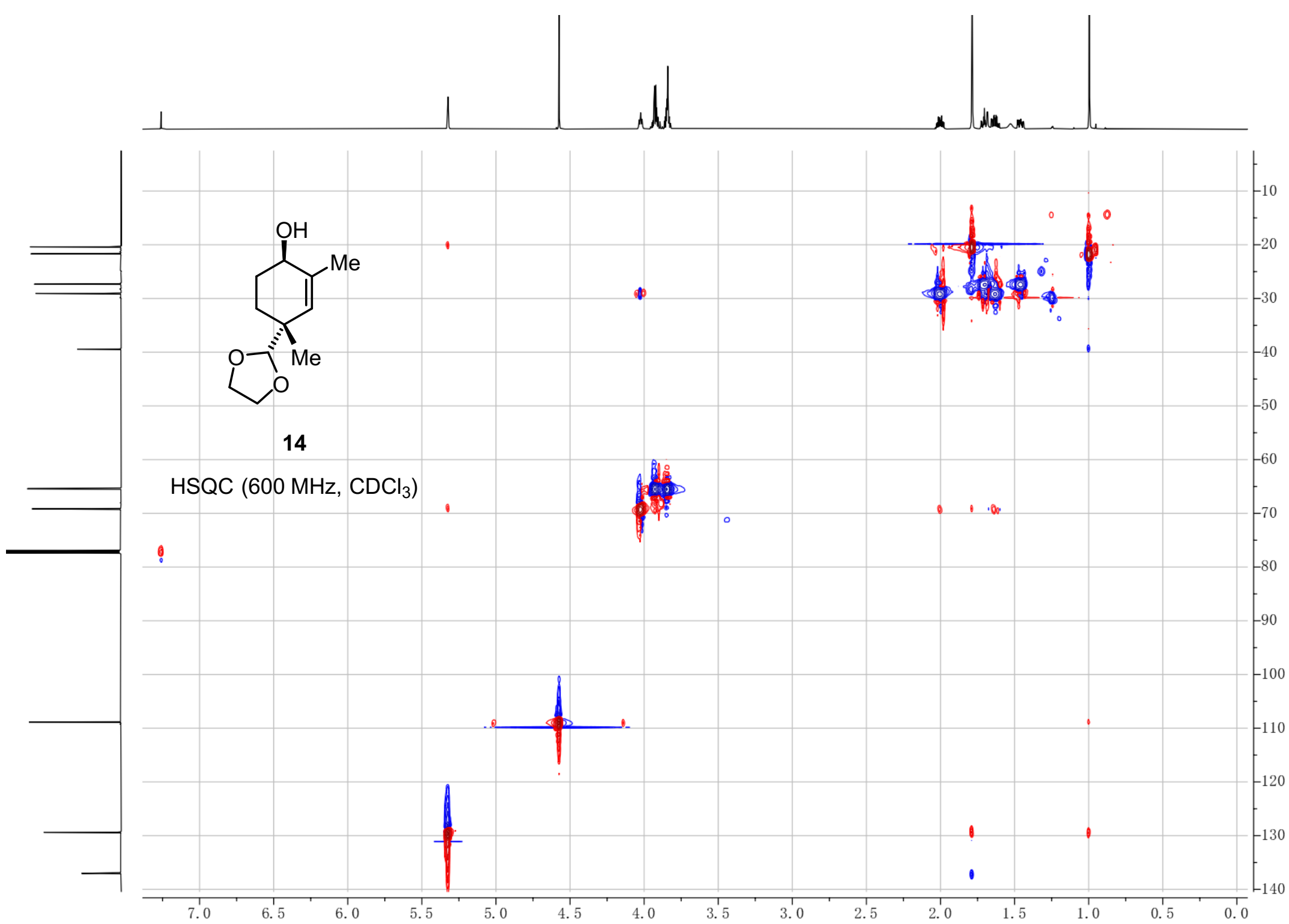
OH
Me
O
O
Me
14
HSQC (600 MHz, CDCl3)
f2 (ppm)

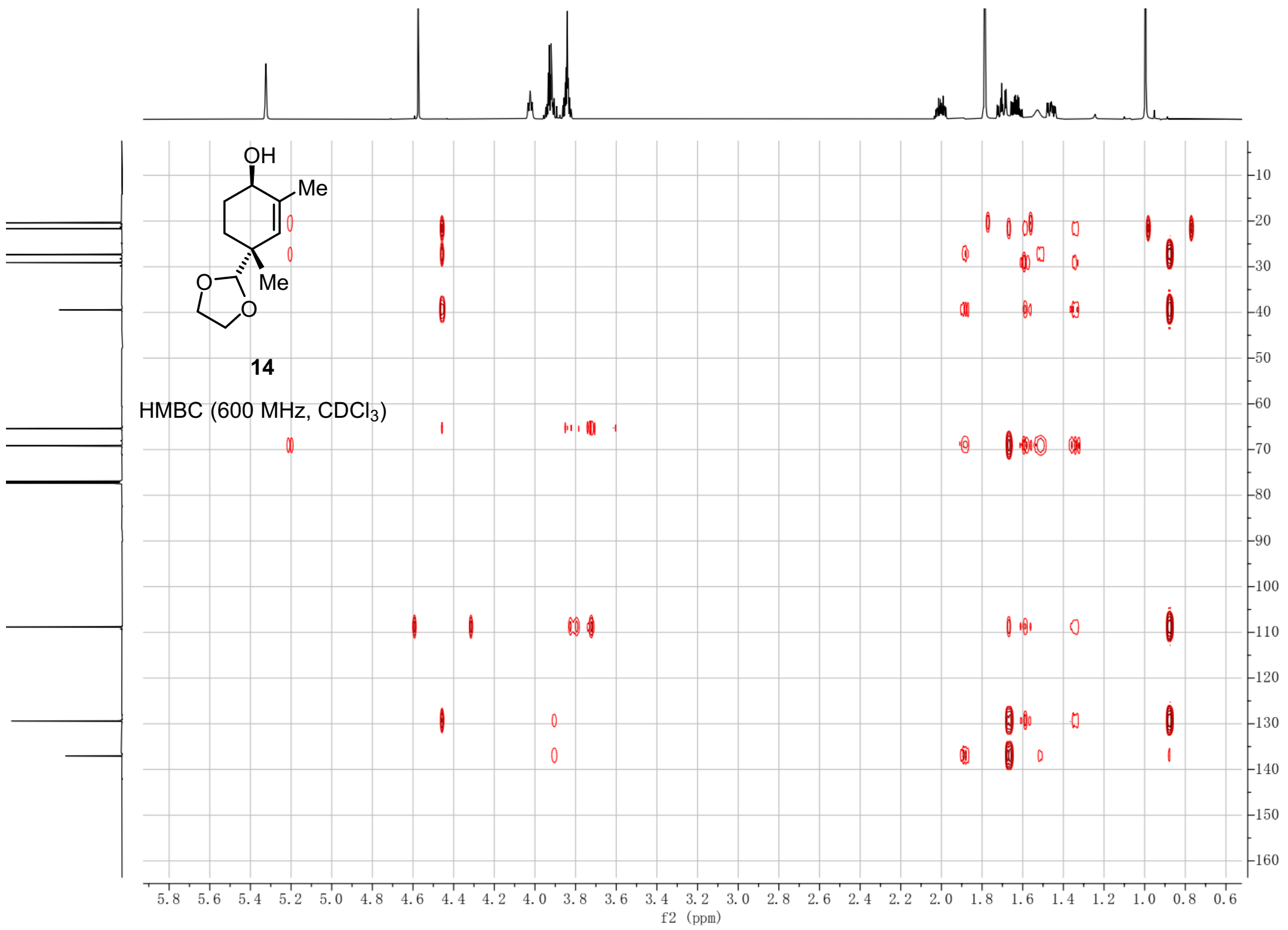
OH
Me
O
O
Me
14
HMBC (600 MHz, CDCl3)
f2 (ppm)

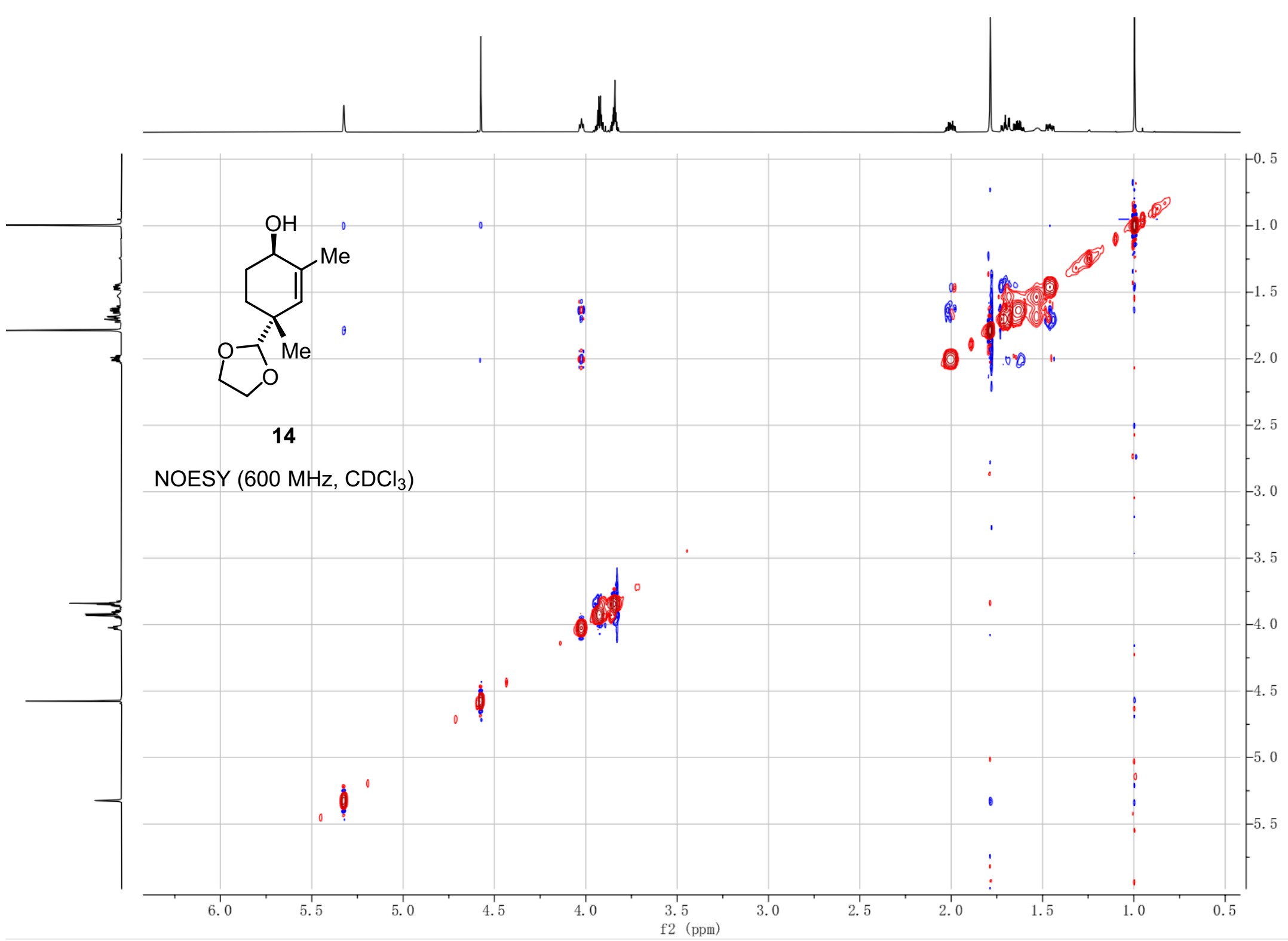
OH
Me
O
O
Me
14
NOESY (600 MHz, CDCl3)
f2 (ppm)

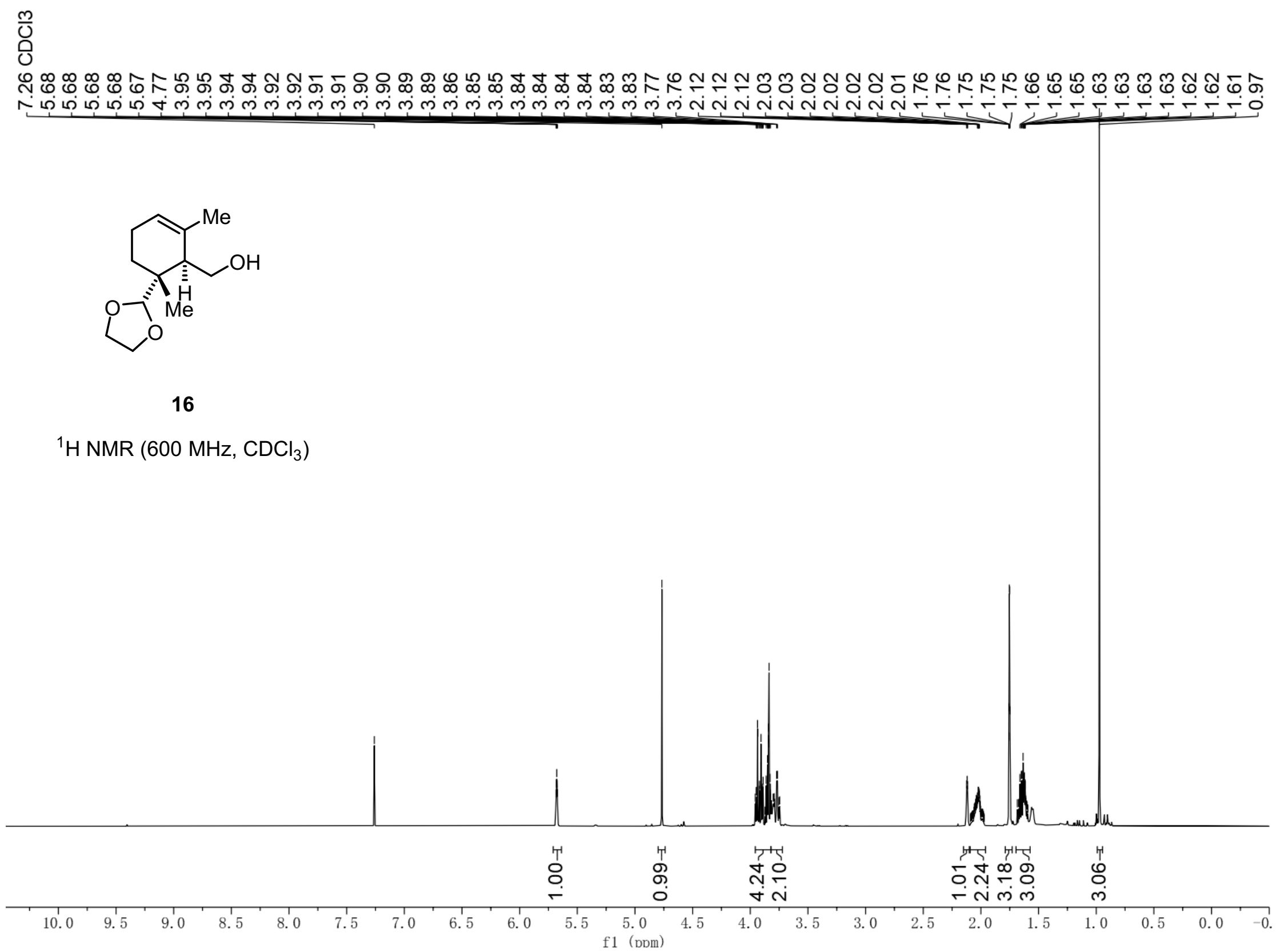

16

^{1}H NMR (600 MHz, $CDCl_3$)

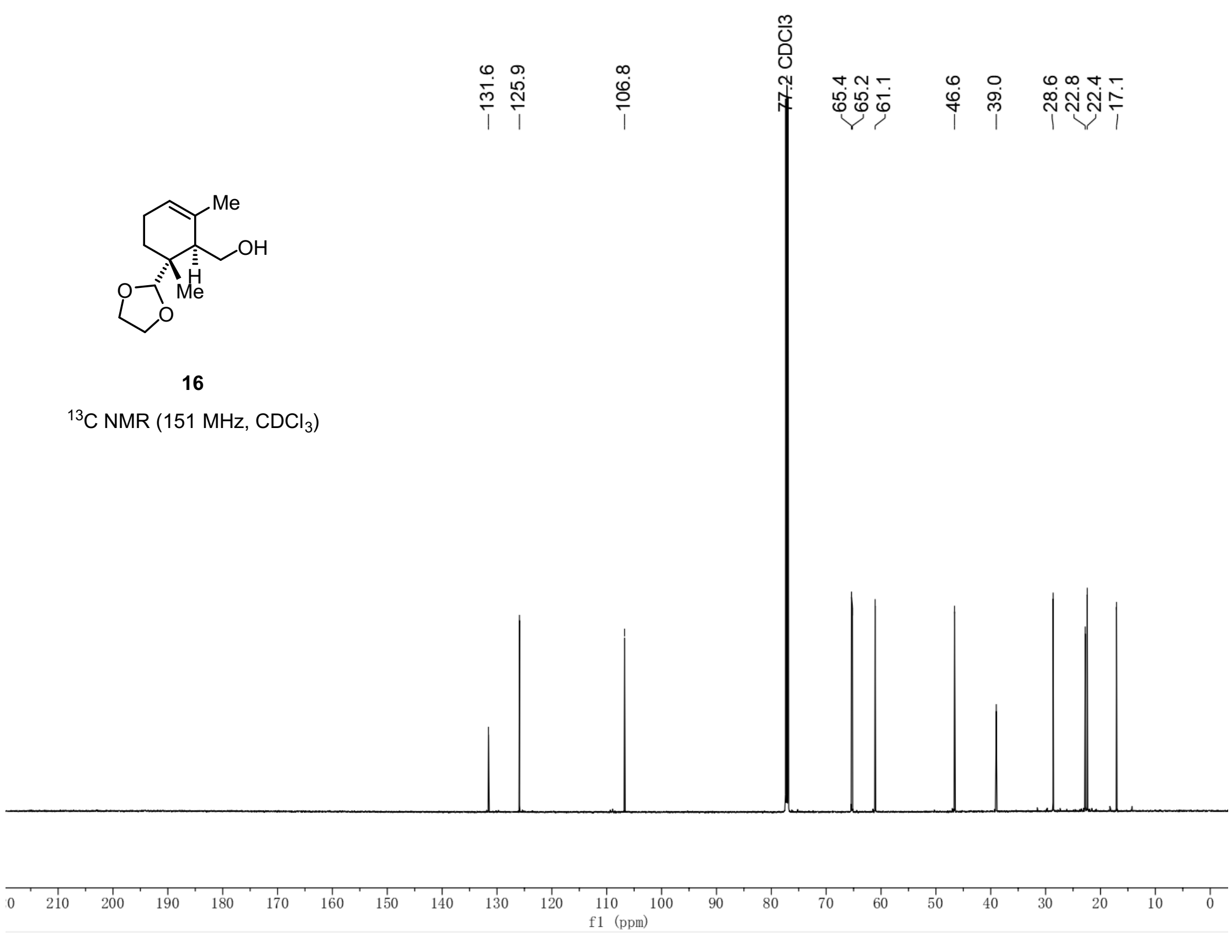

16

^{13}C NMR (151 MHz, $CDCl_3$)

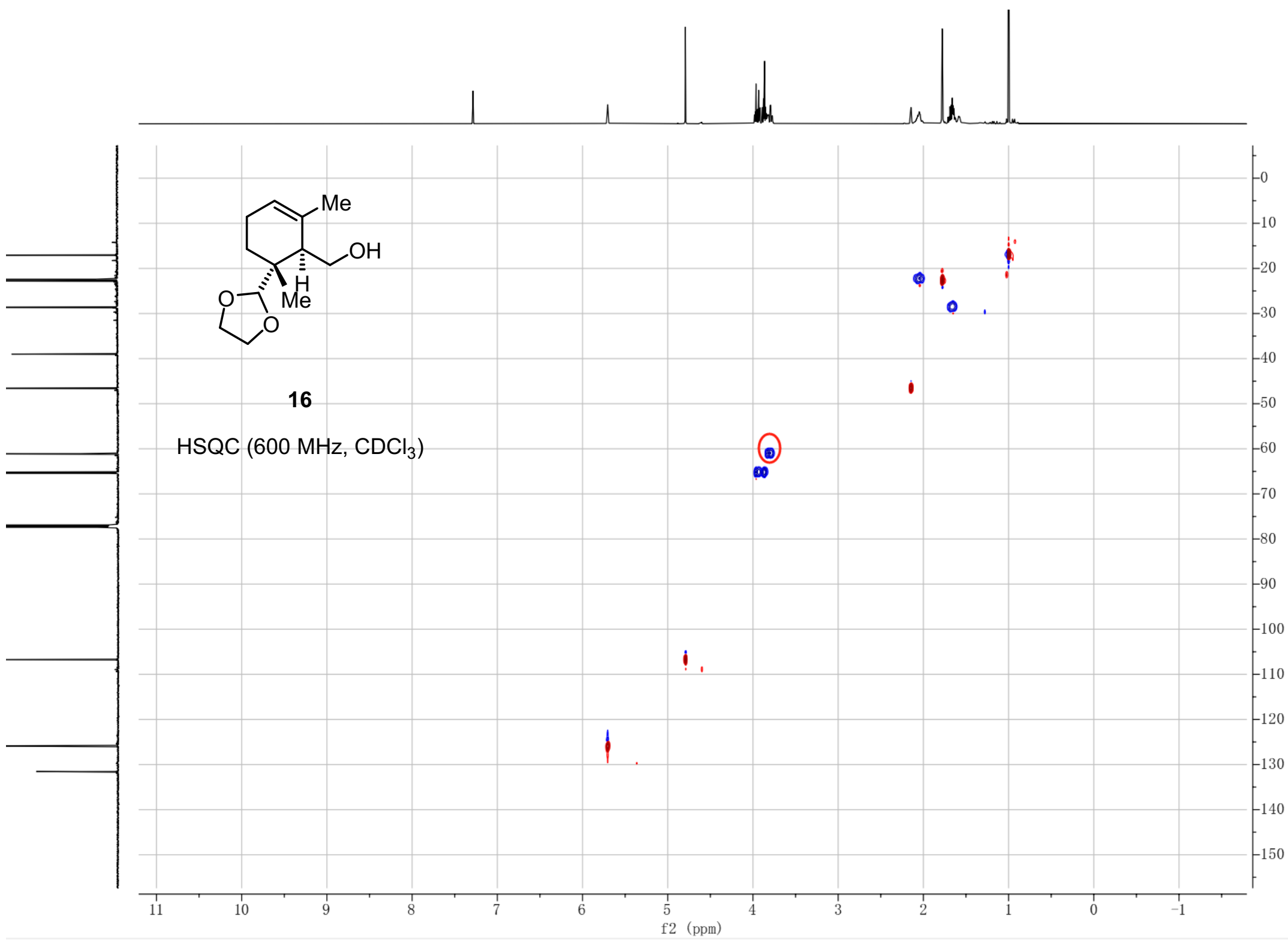
Me
OH
H
Me
O
O
16
HSQC (600 MHz, CDCl$_3$)
f2 (ppm)

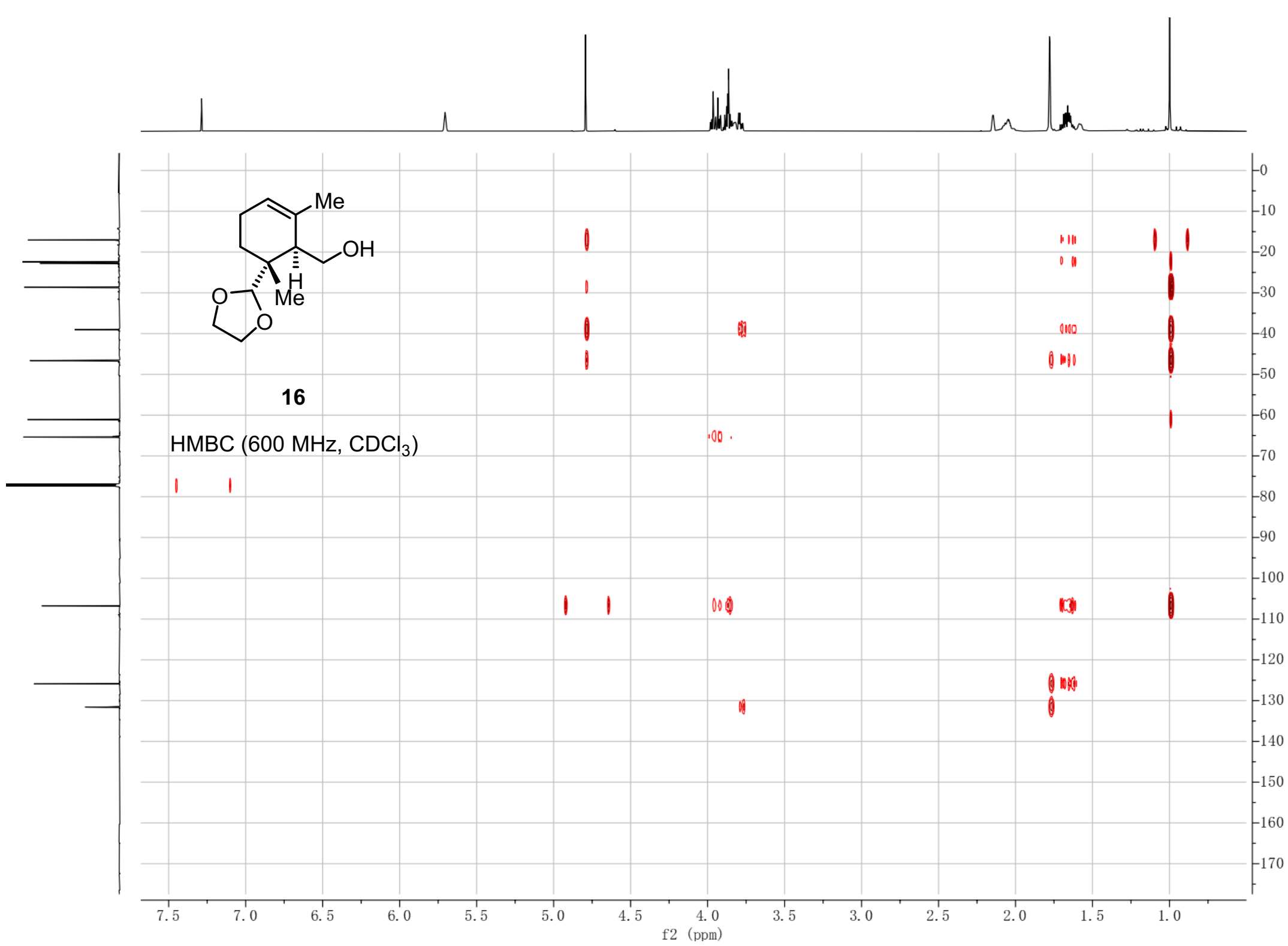
Me
OH
H
Me
O
O
16
HMBC (600 MHz, CDCl$_3$)
f2 (ppm)

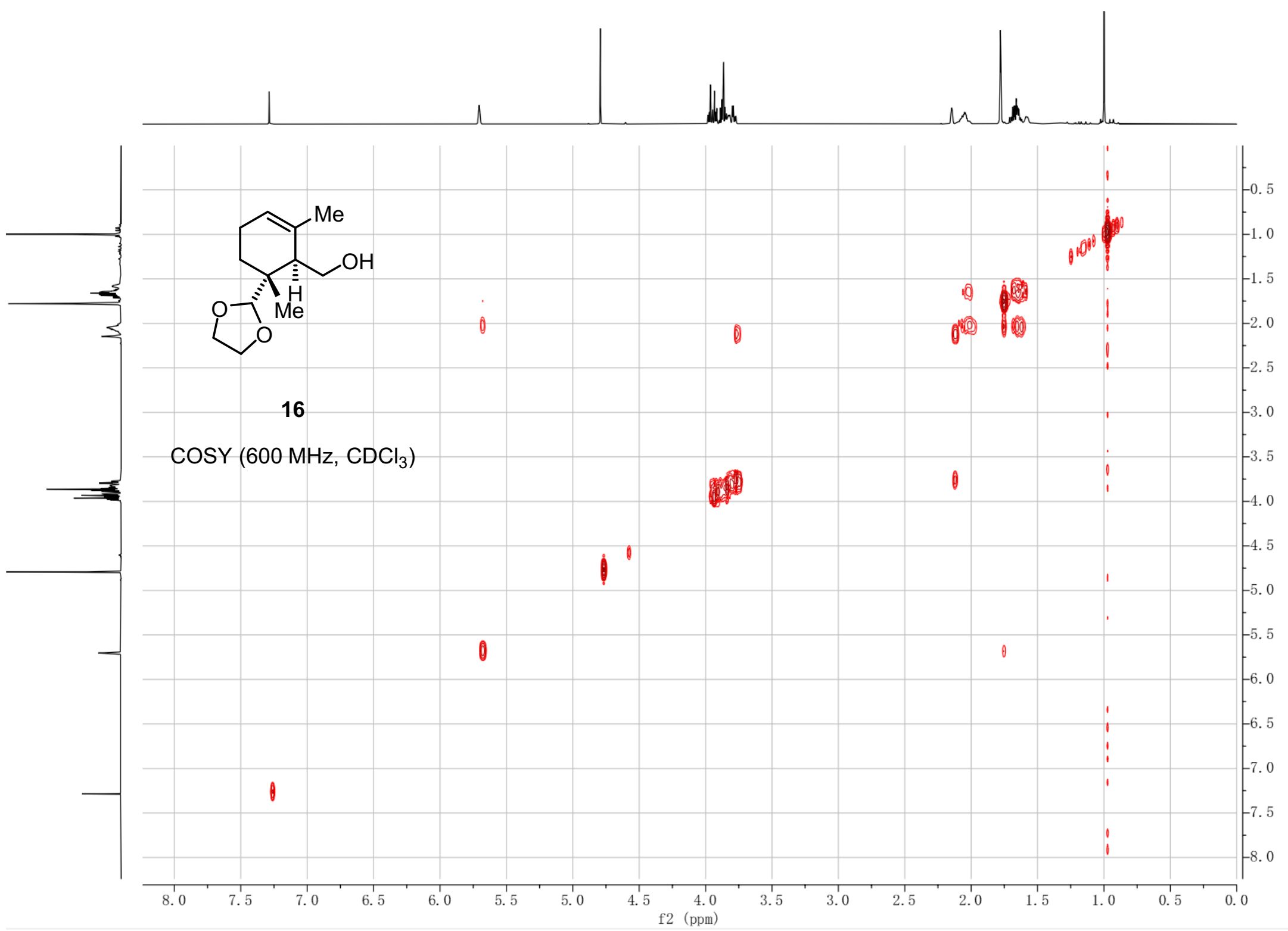
Me
OH
H
Me
O
O
16
COSY (600 MHz, CDCl3)
f2 (ppm)

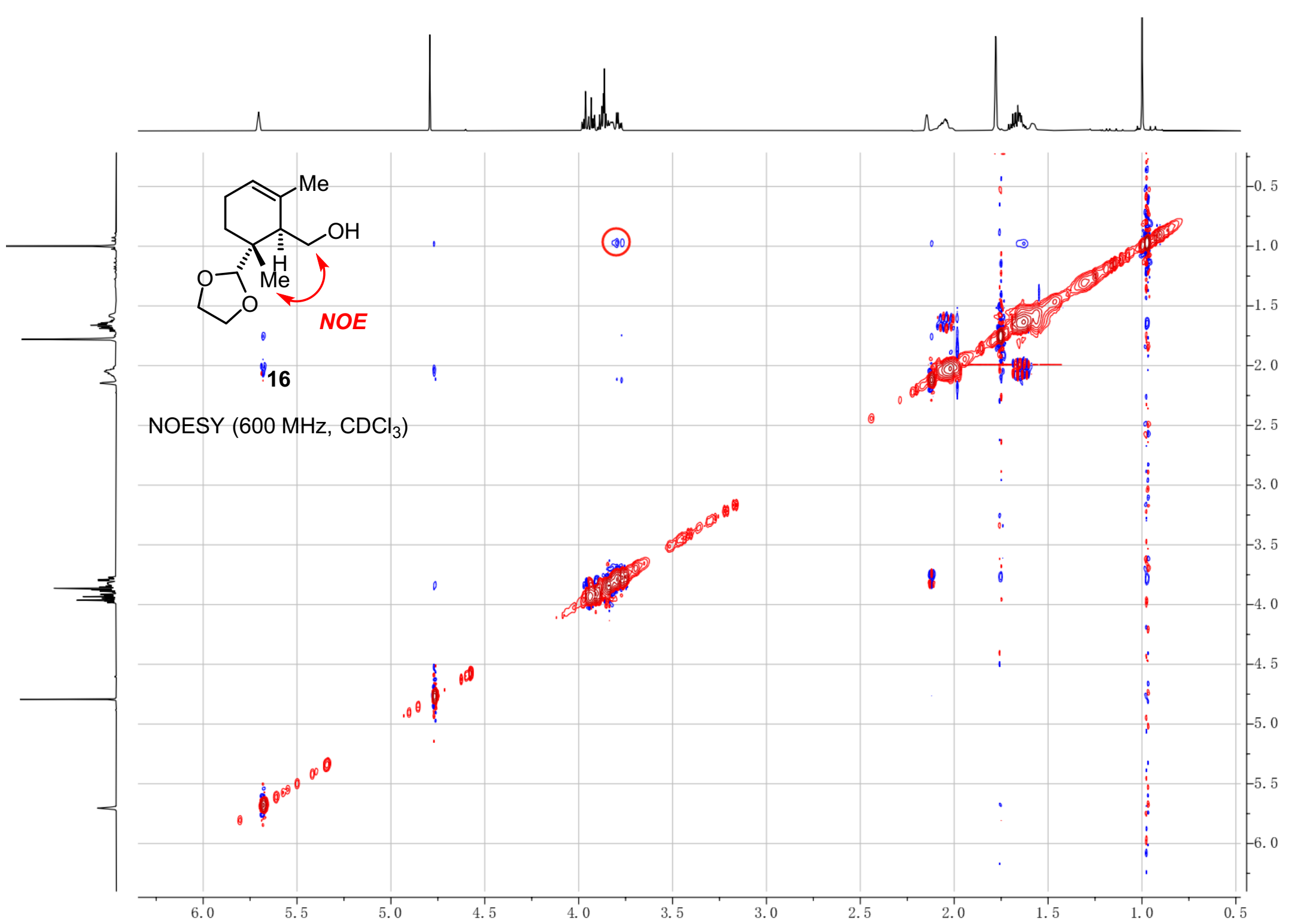
Me
OH
H
Me
O
O
NOE
16
NOESY (600 MHz, CDCl3)
f2 (ppm)

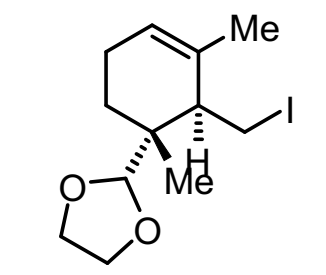

17

^{1}H NMR (400 MHz, $CDCl_3$)

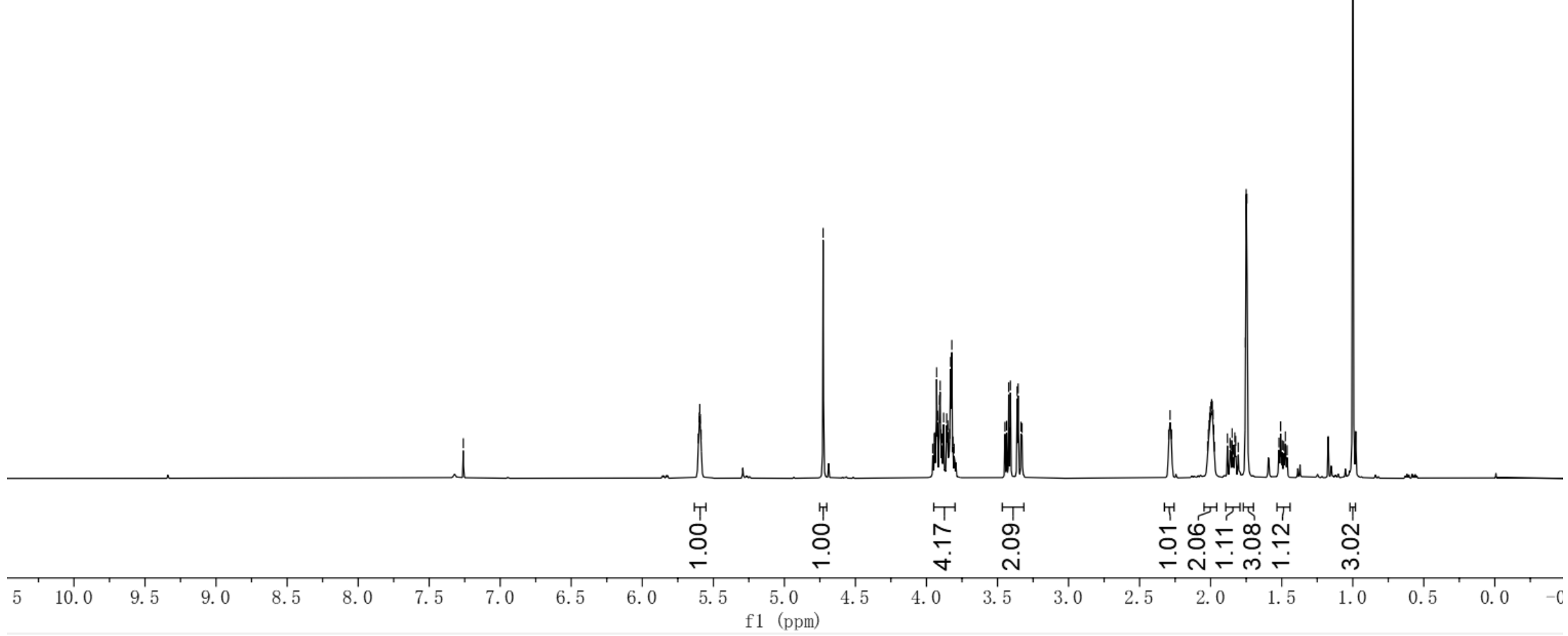

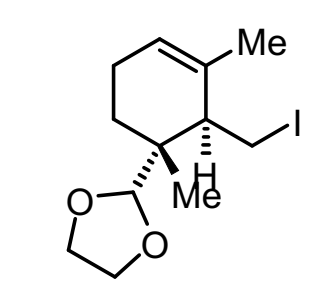

17

^{13}C NMR (101 MHz, $CDCl_3$)

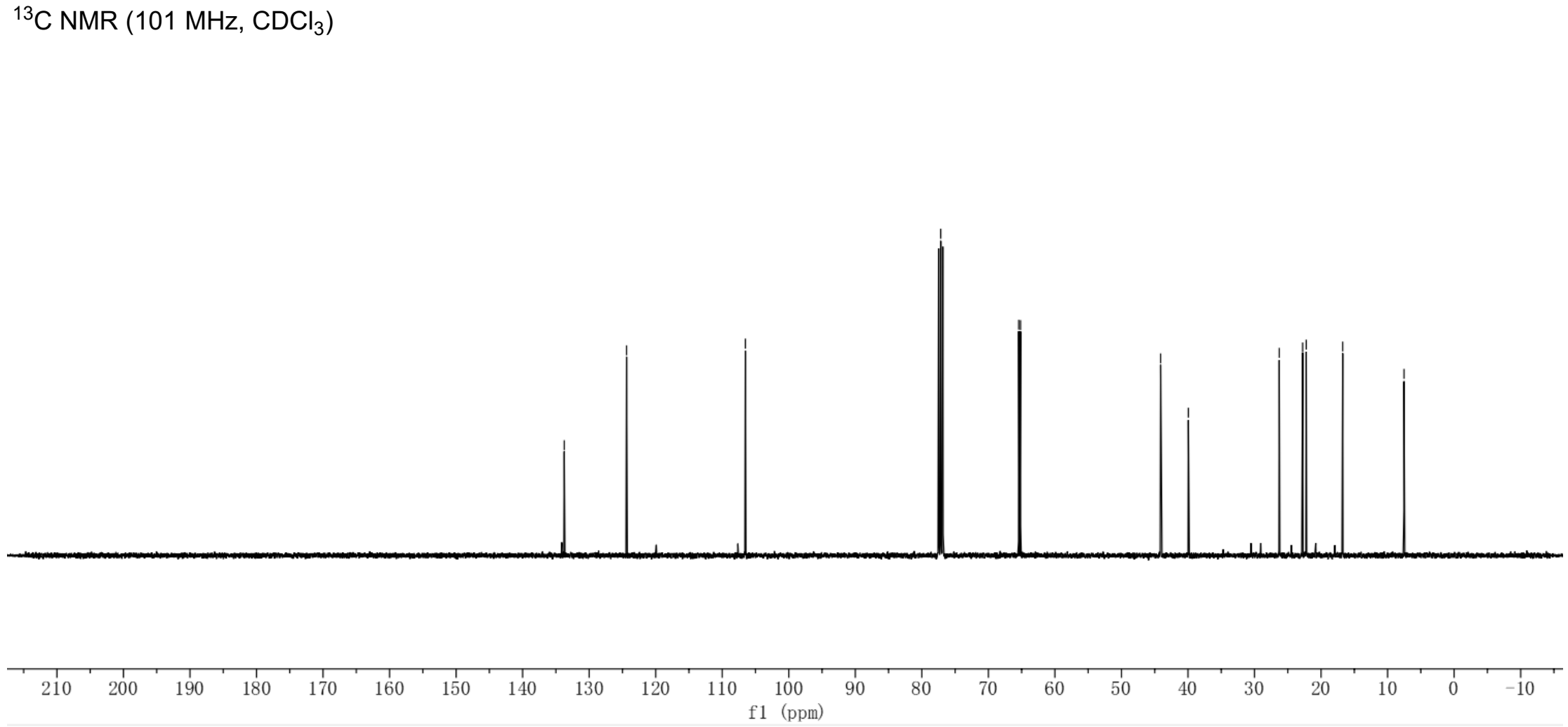

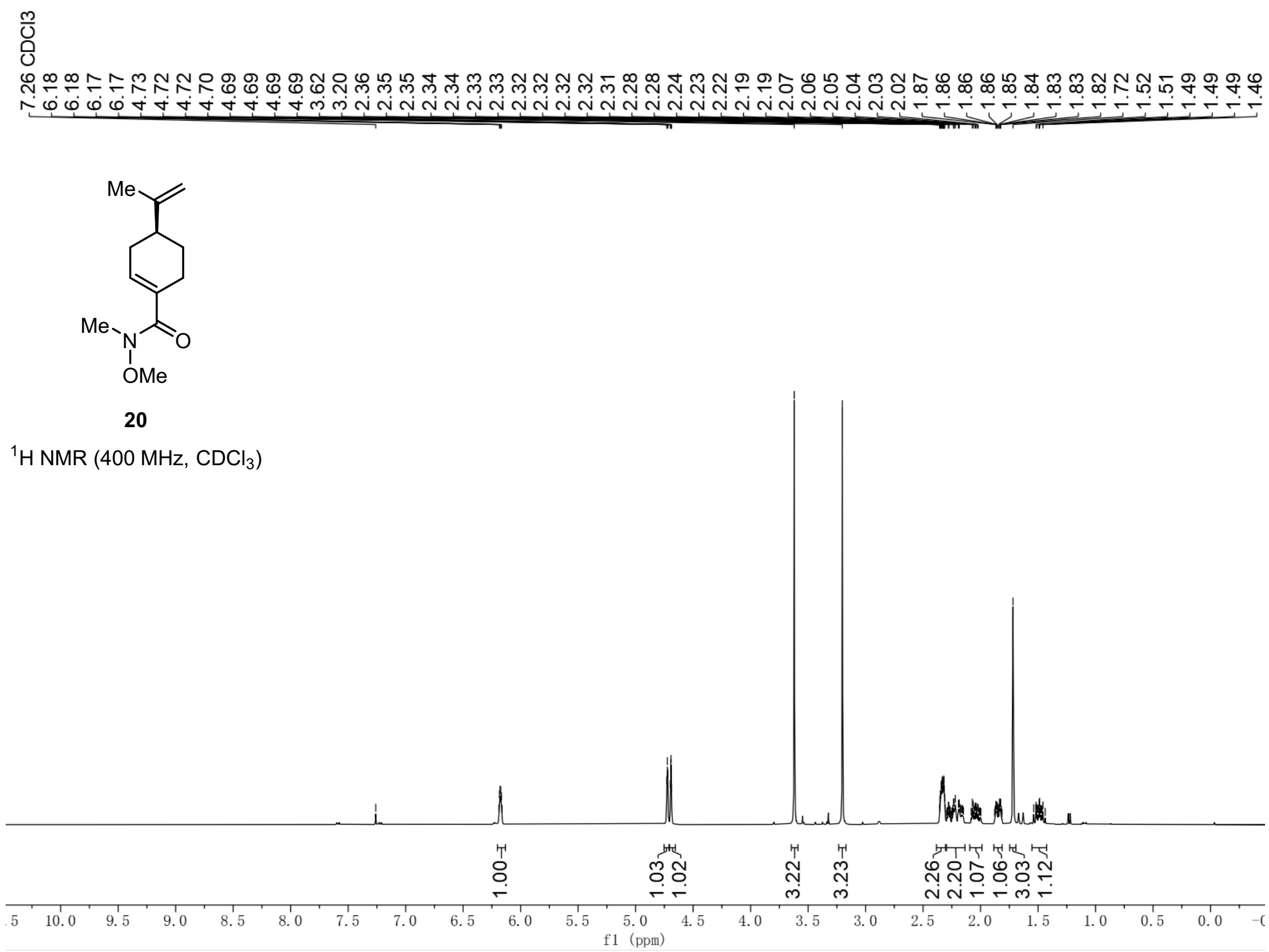

20

^{1}H NMR (400 MHz, $CDCl_3$)

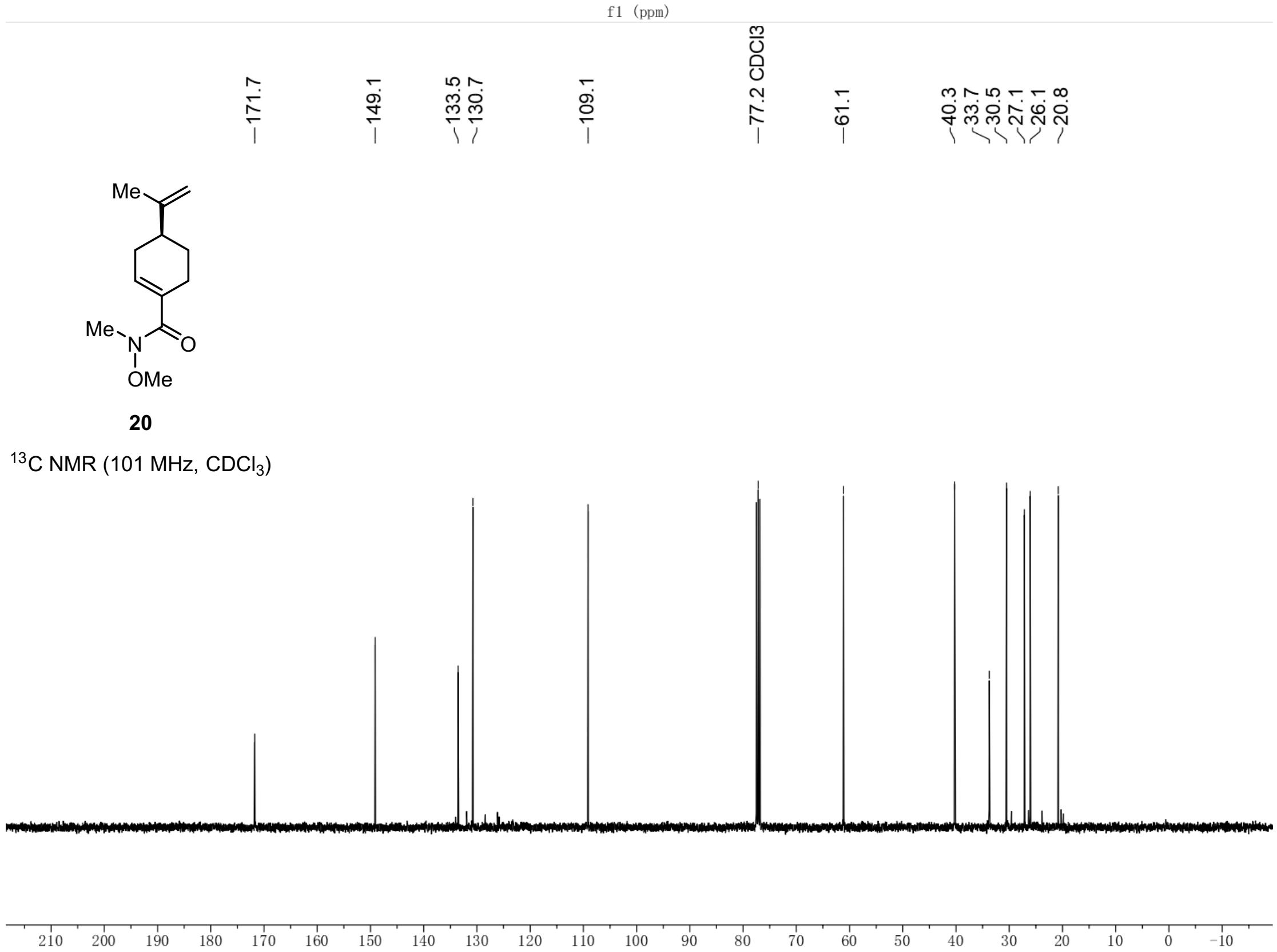

20

^{13}C NMR (101 MHz, $CDCl_3$)

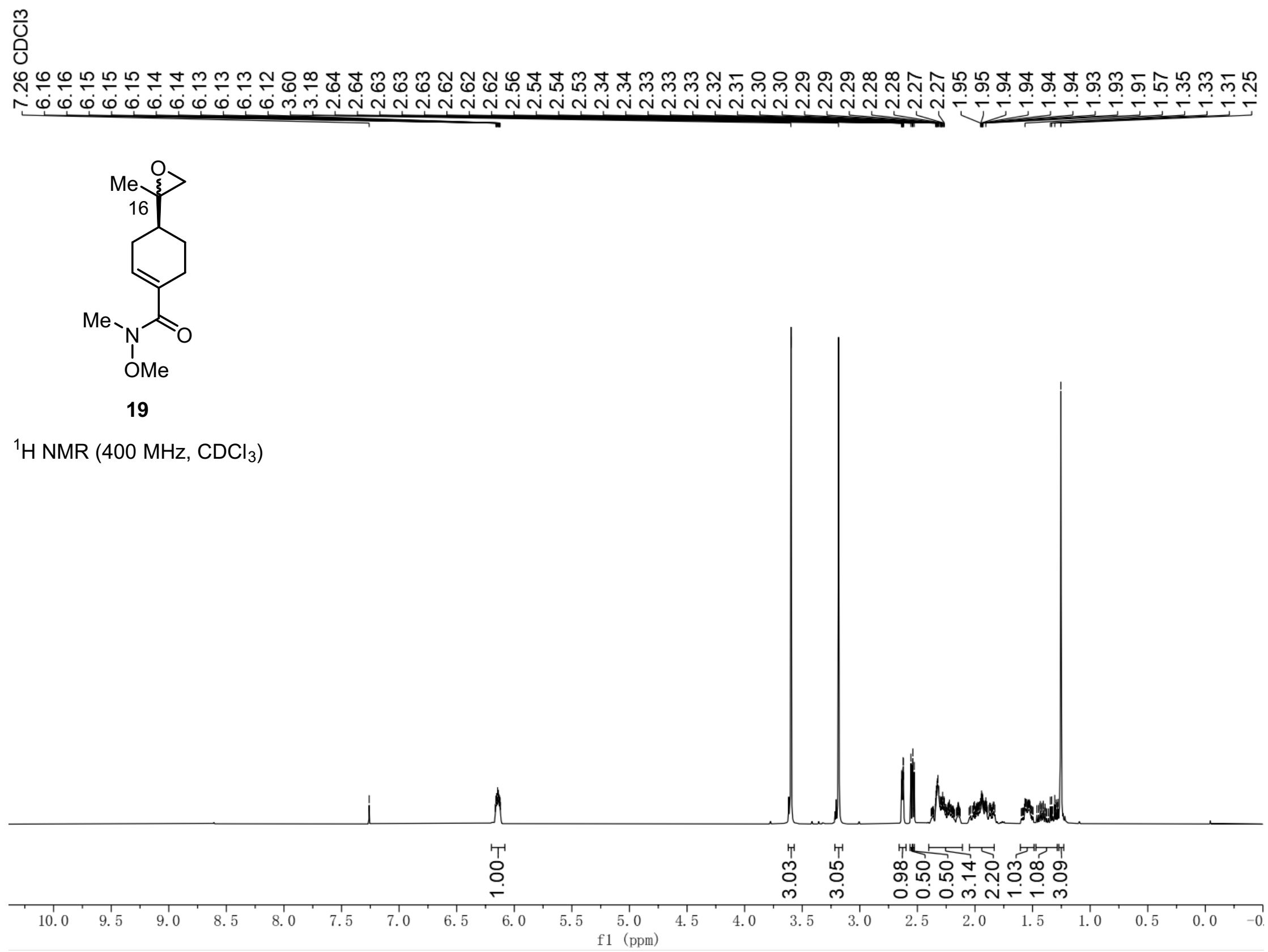

19

^{1}H NMR (400 MHz, $CDCl_3$)

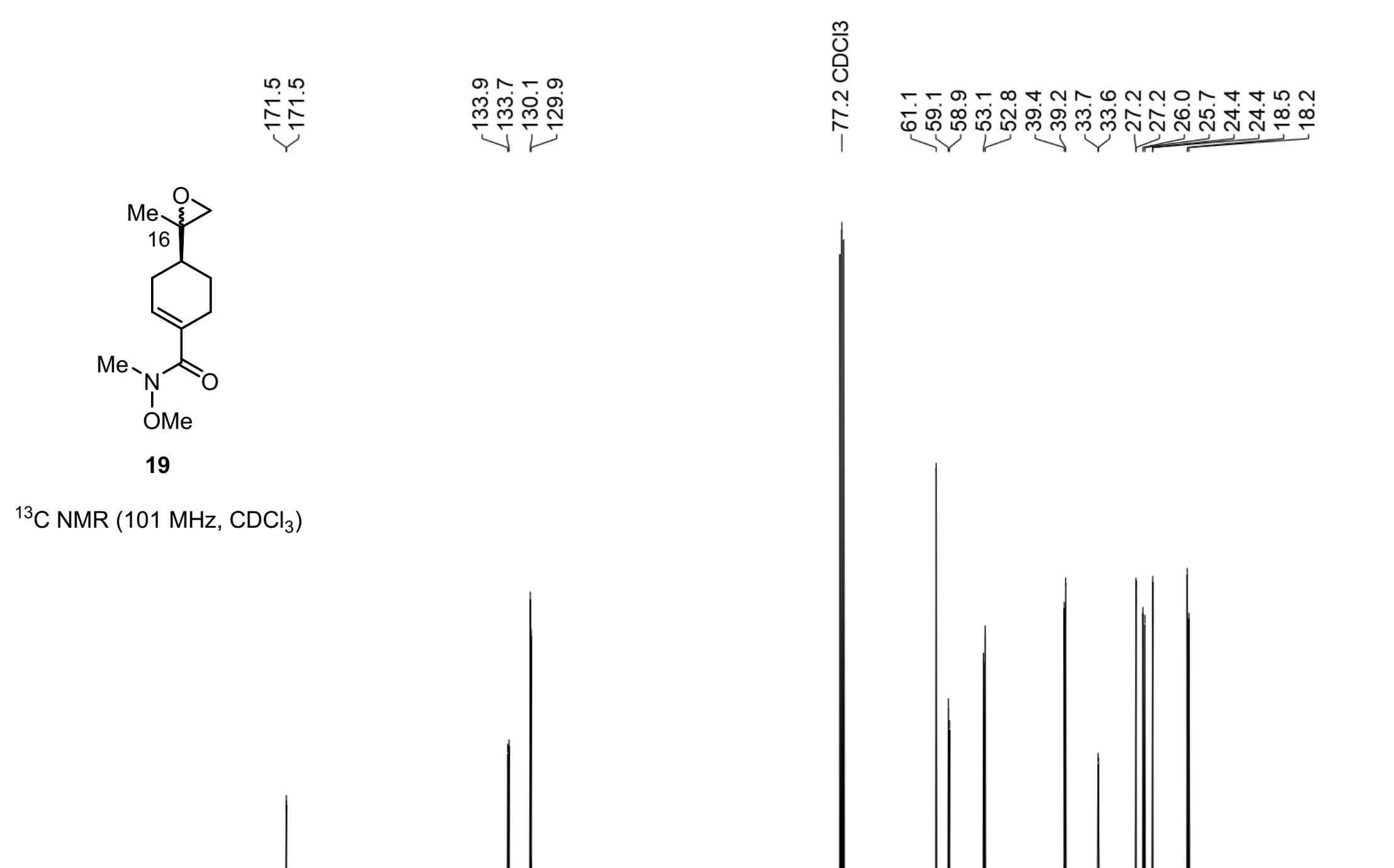

19

^{13}C NMR (101 MHz, $CDCl_3$)

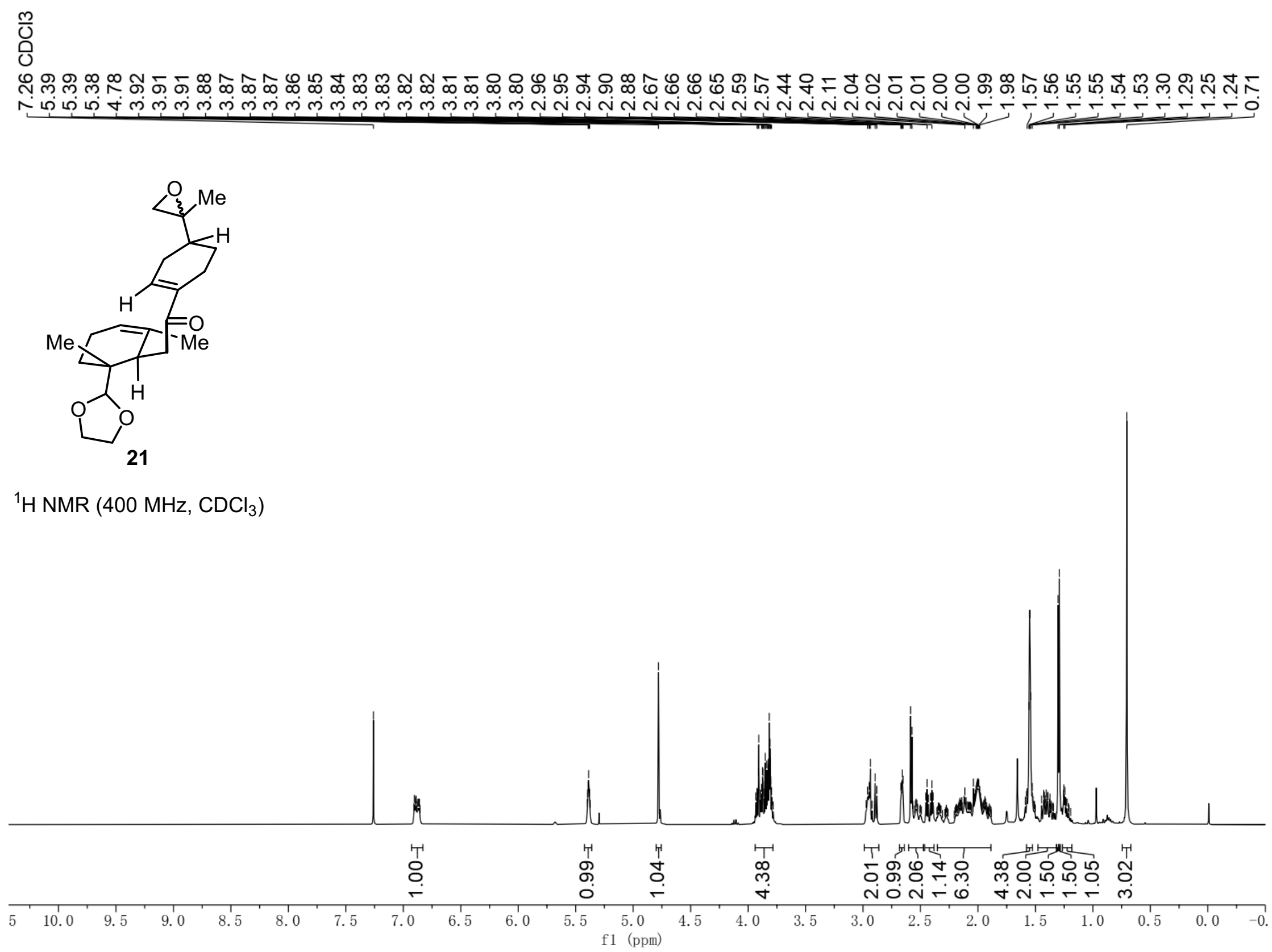
7.26 CDCl3
5.39
5.39
5.38
4.78
3.92
3.91
3.91
3.88
3.87
3.87
3.87
3.86
3.85
3.84
3.83
3.83
3.82
3.82
3.81
3.81
3.80
3.80
2.96
2.95
2.94
2.90
2.88
2.67
2.66
2.66
2.65
2.59
2.57
2.44
2.40
2.11
2.04
2.02
2.01
2.01
2.00
2.00
1.99
1.98
1.57
1.56
1.55
1.55
1.54
1.53
1.30
1.29
1.25
1.24
0.71
Me
H
H
O
Me
Me
H
O
O
O
21
^{1}H NMR (400 MHz, CDCl$_3$)
1.00
0.99
1.04
4.38
2.01
0.99
2.06
1.14
6.30
4.38
2.00
1.50
1.50
1.05
3.02
f1 (ppm)

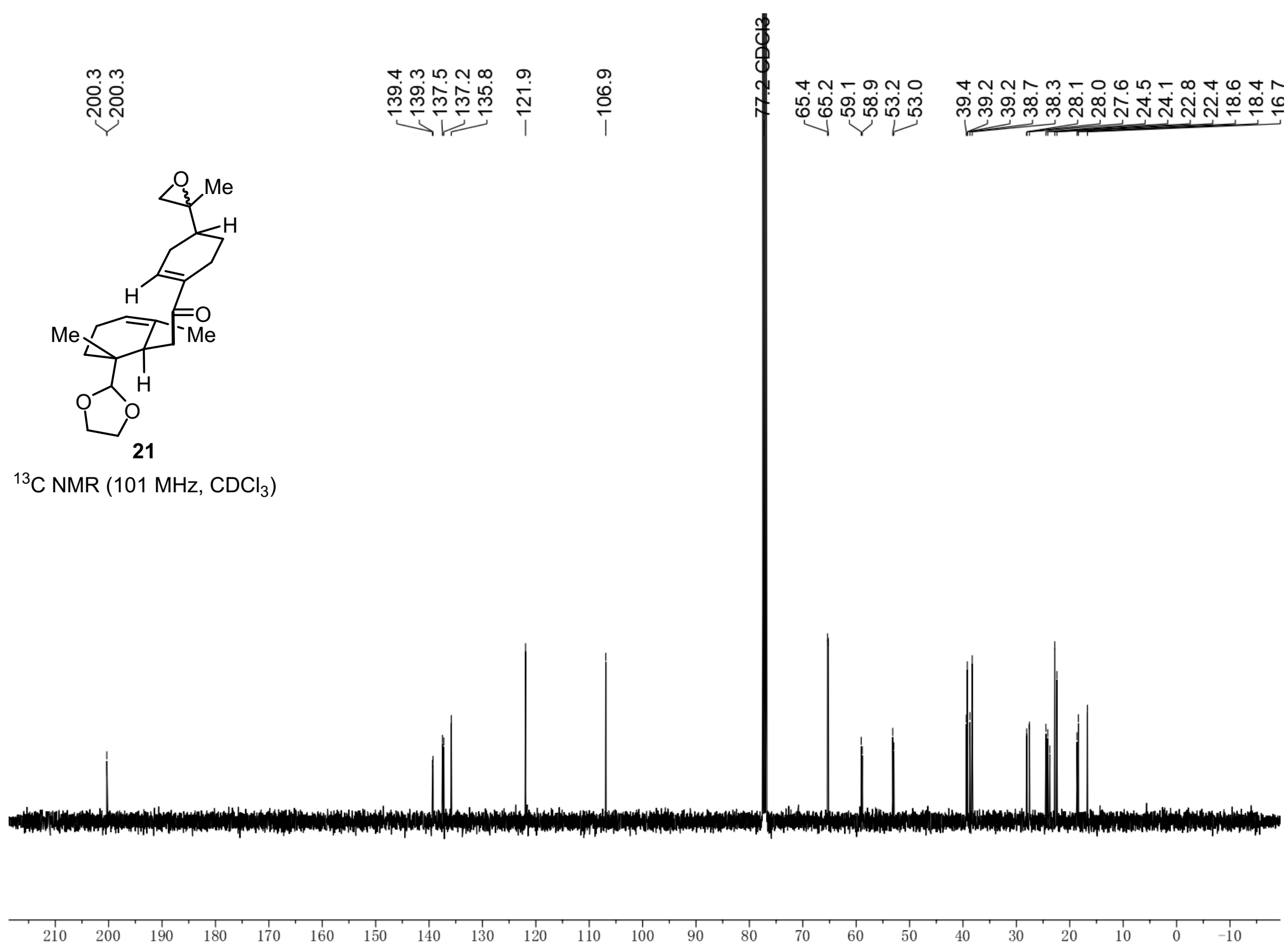
200.3
200.3
139.4
139.3
137.5
137.2
135.8
121.9
106.9
77.2 CDCl3
65.4
65.2
59.1
58.9
53.2
53.0
39.4
39.2
38.7
38.3
28.1
28.0
27.6
24.5
24.1
22.8
22.4
18.6
18.4
16.7
Me
H
H
O
Me
Me
H
O
O
O
21
^{13}C NMR (101 MHz, CDCl$_3$)

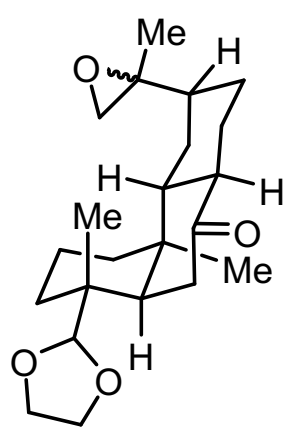

22

^{1}H NMR (400 MHz, $CDCl_3$)

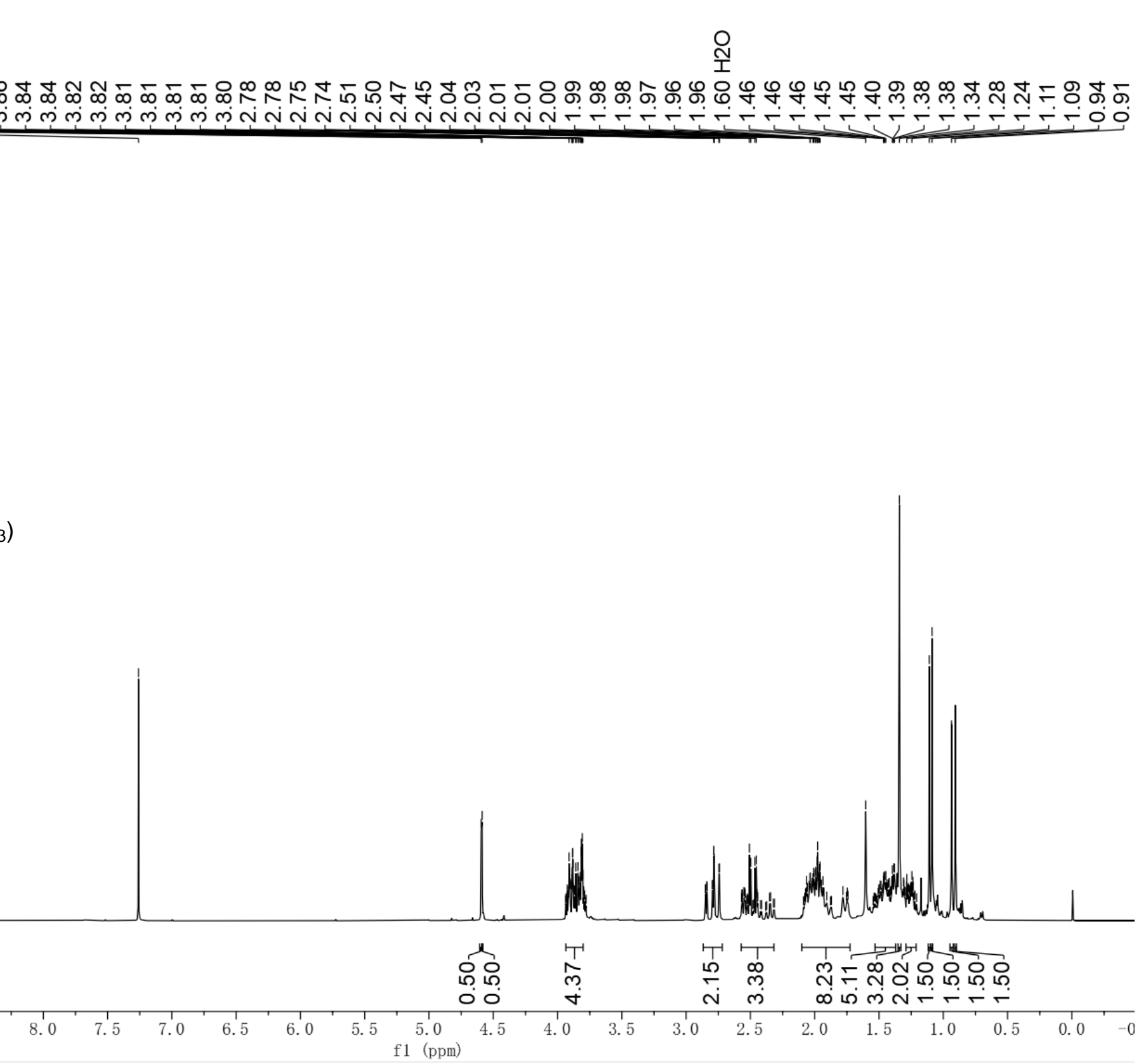

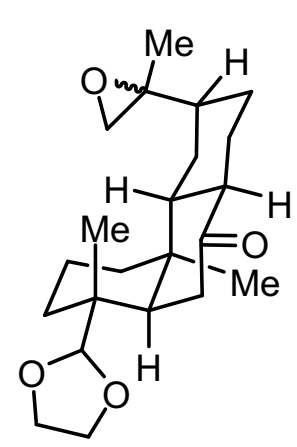

22

^{13}C NMR (101 MHz, $CDCl_3$)

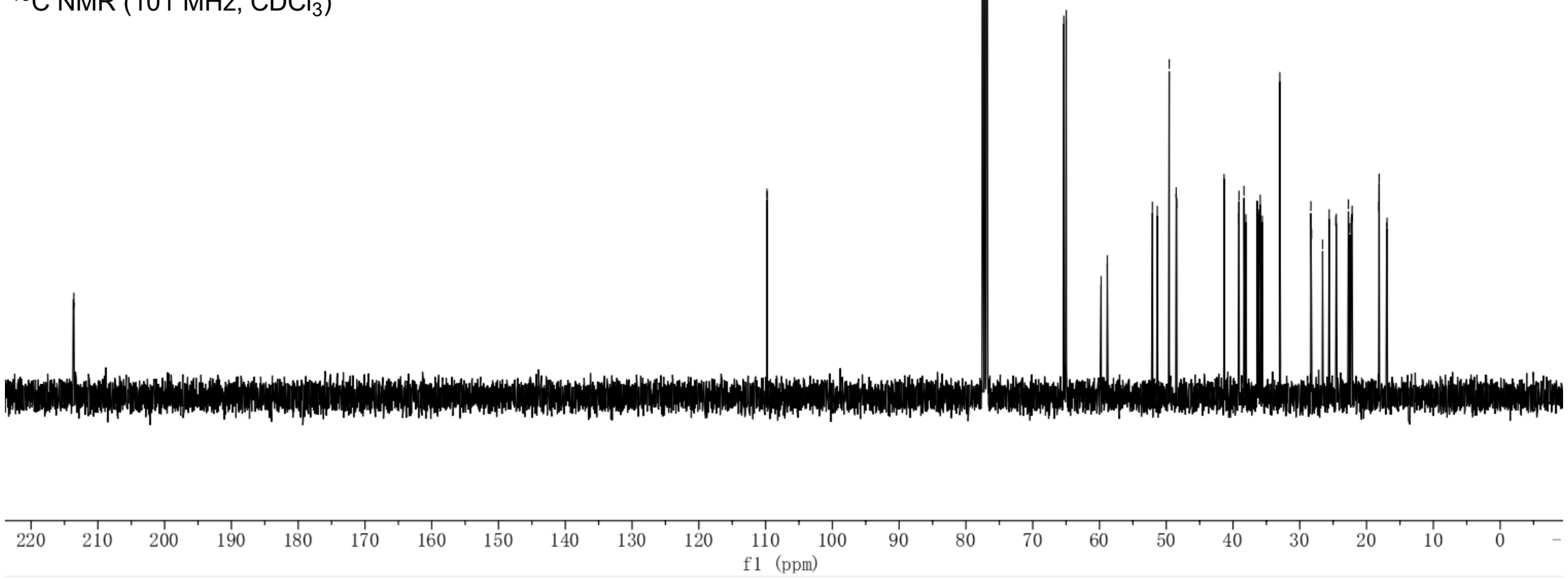

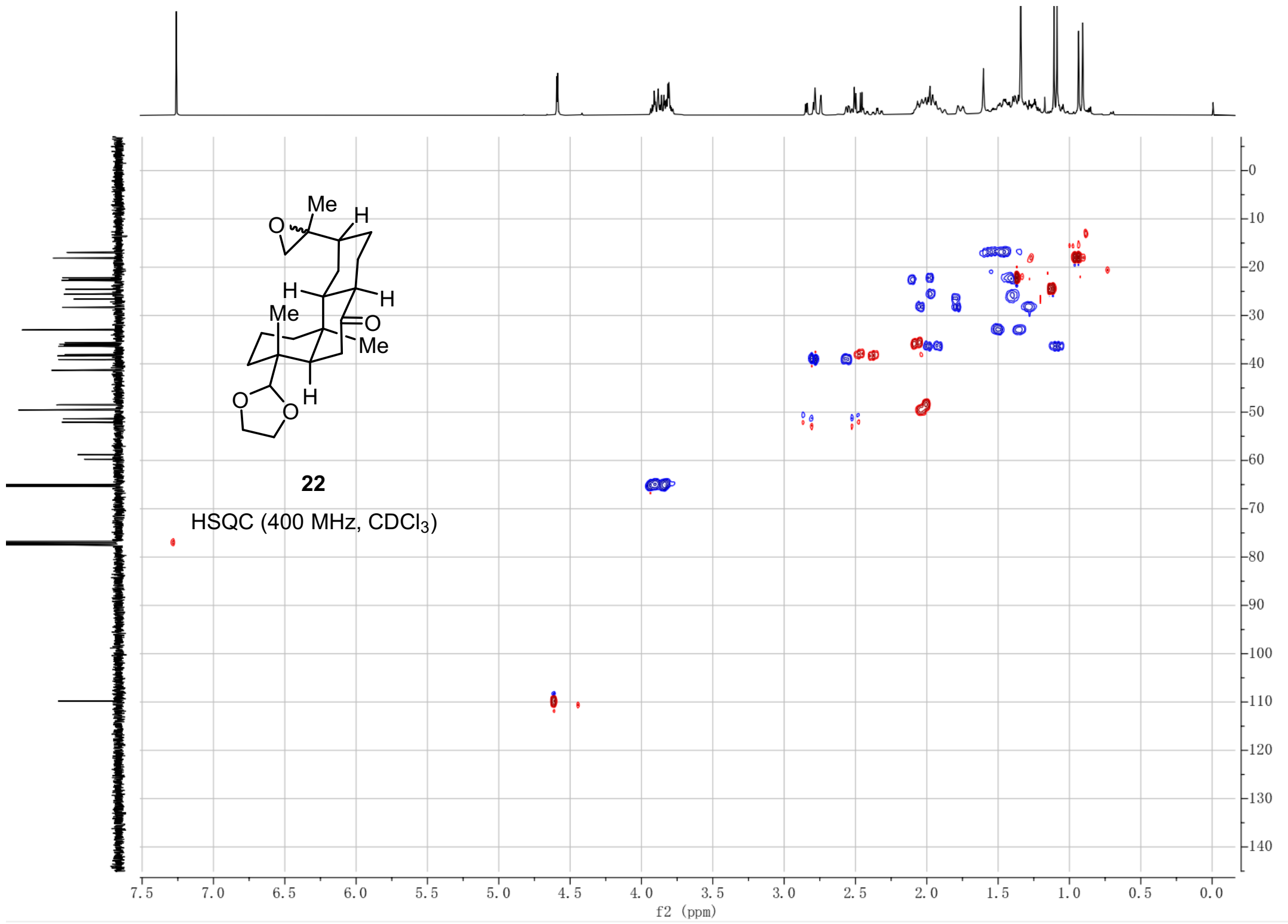
22
HSQC (400 MHz, CDCl$_3$)
f2 (ppm)

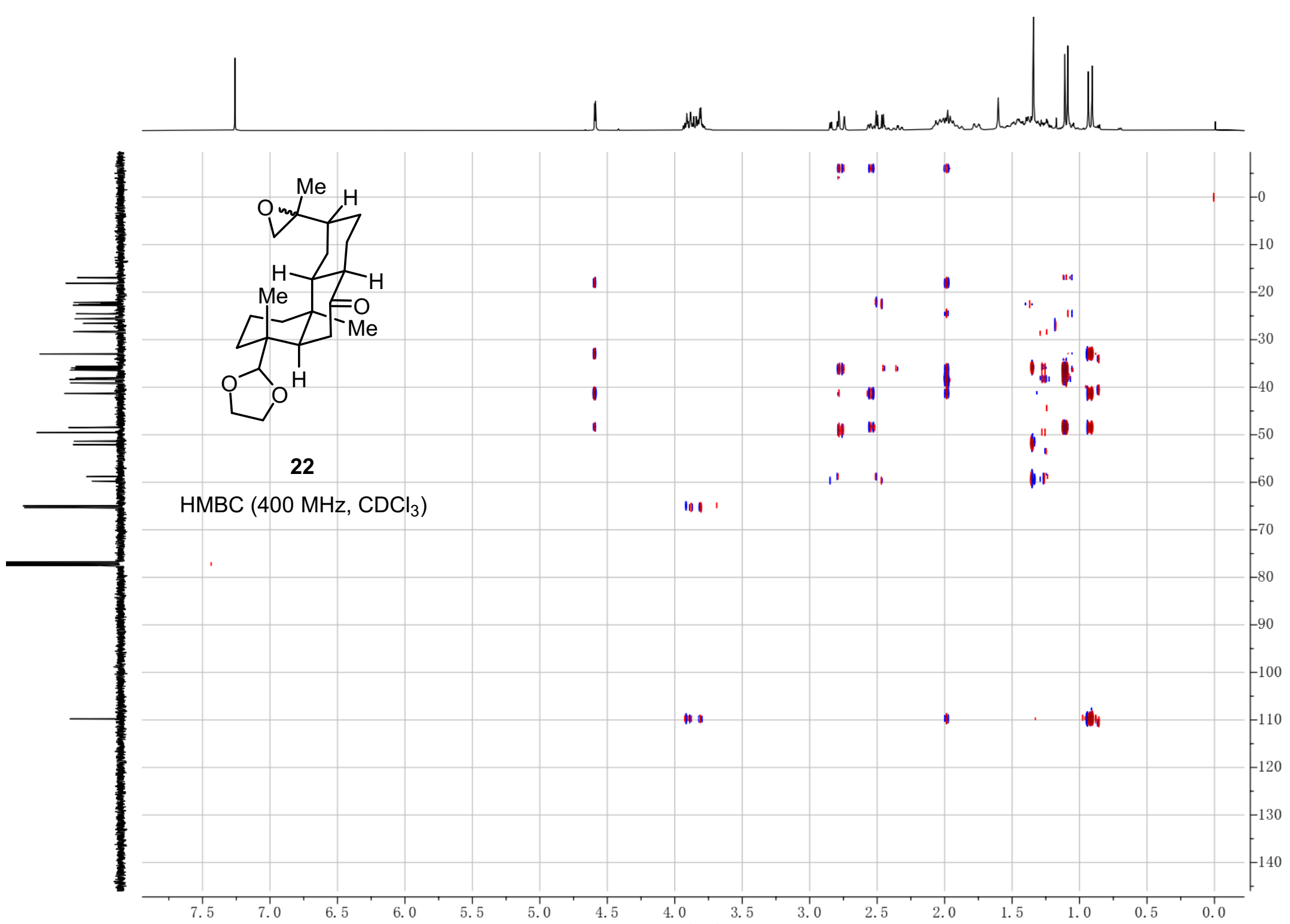
22
HMBC (400 MHz, CDCl$_3$)
f2 (ppm)

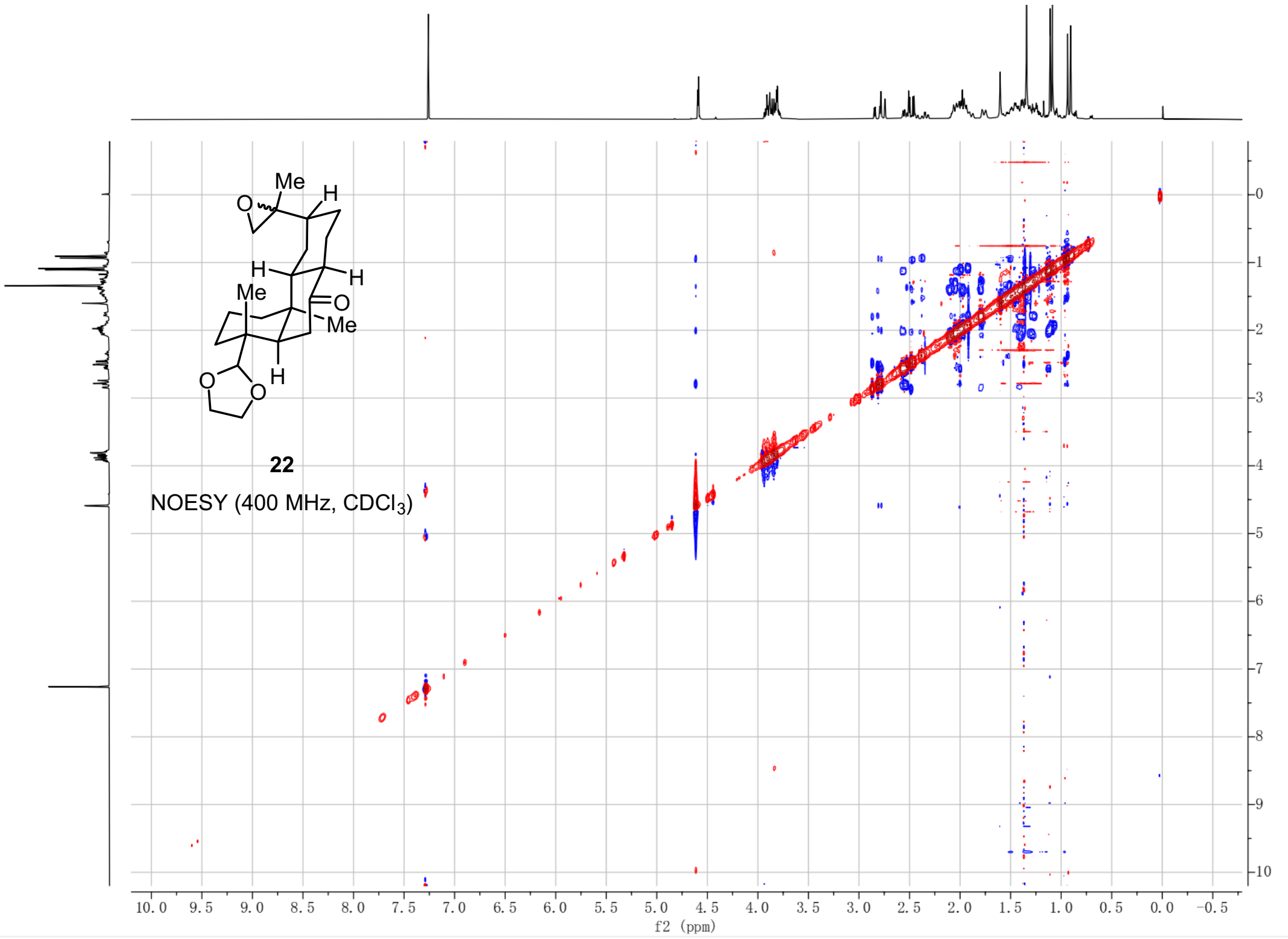
Me
H
O
H
H
Me
O
Me
O
O
H
22
NOESY (400 MHz, CDCl3)
f2 (ppm)

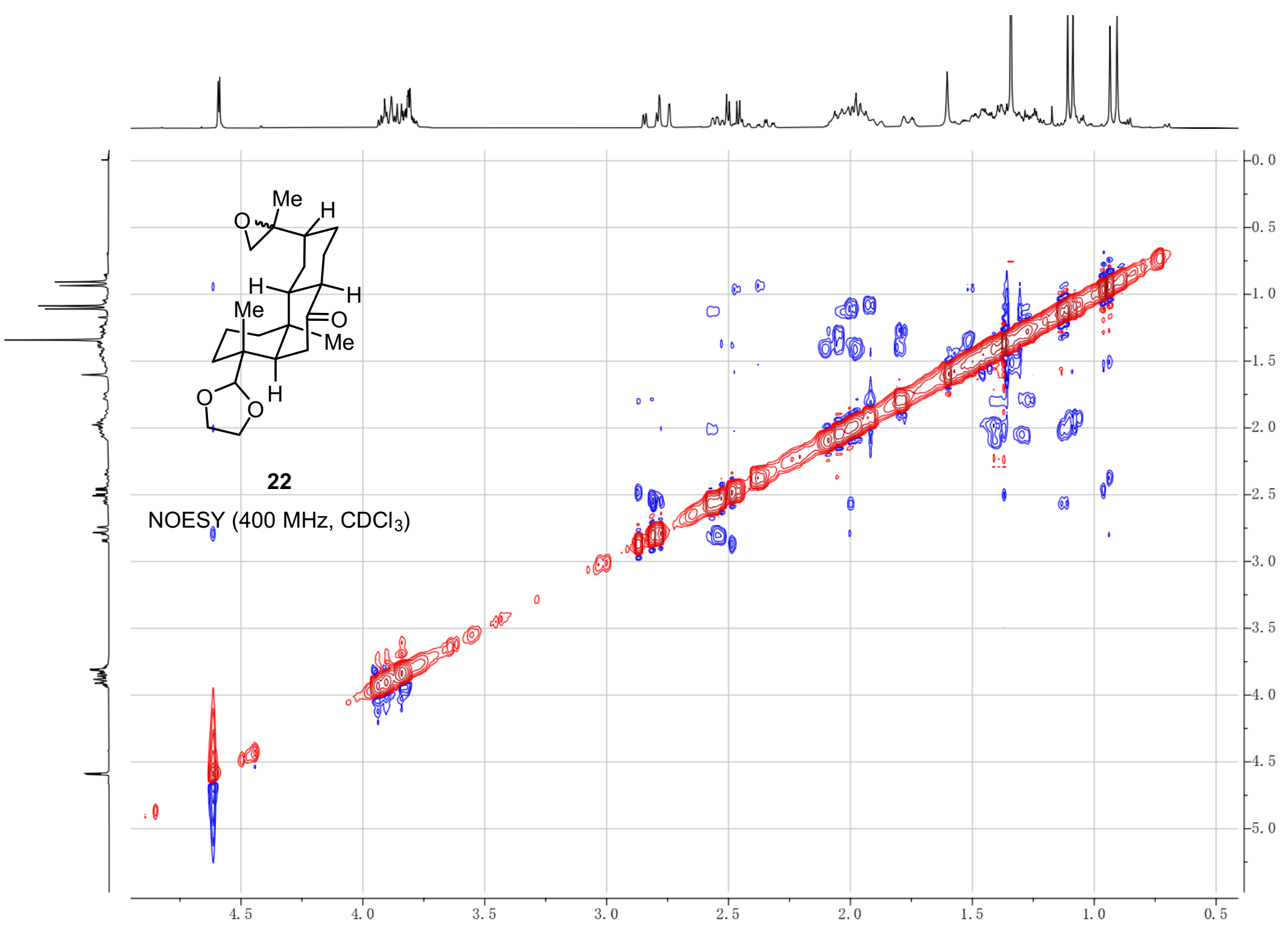
Me
H
O
H
H
Me
O
Me
O
O
H
22
NOESY (400 MHz, CDCl3)
f2 (ppm)

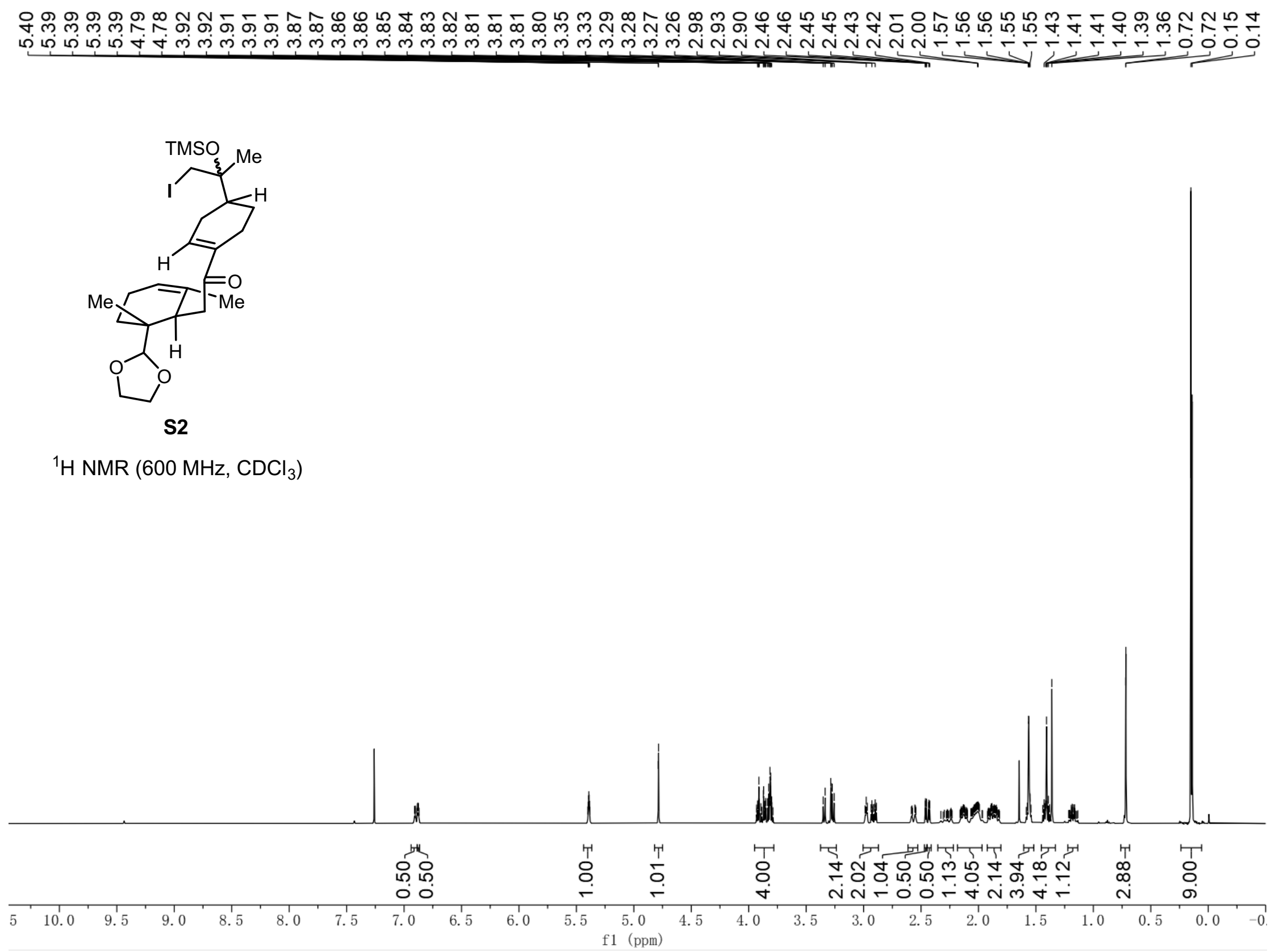
TMSO
Me
I
H
H
O
Me
Me
H
O
O
S2
^{1}H NMR (600 MHz, CDCl$_3$)
f1 (ppm)

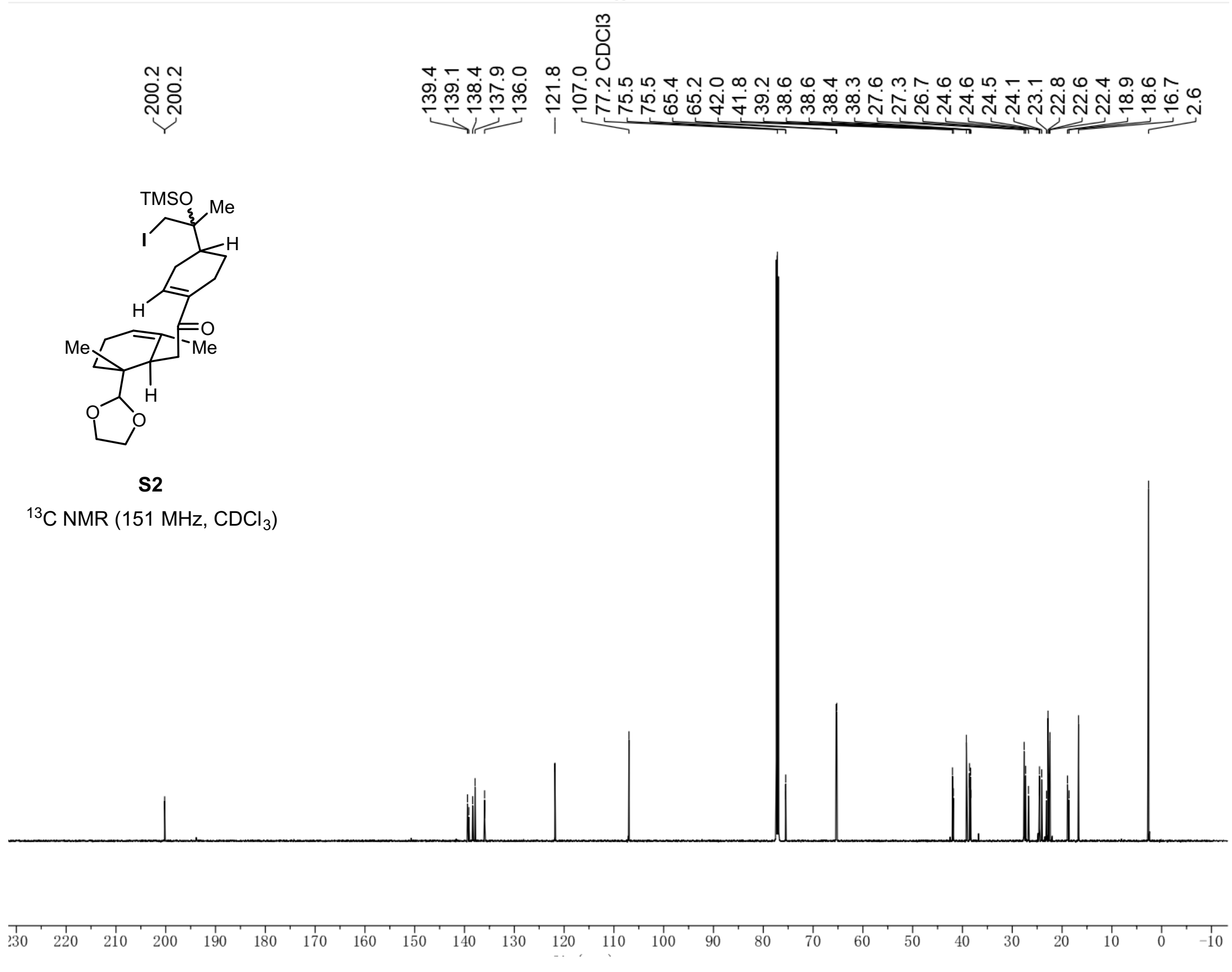
TMSO
Me
I
H
H
O
Me
Me
H
O
O
S2
^{13}C NMR (151 MHz, CDCl$_3$)
77.2 CDCl3

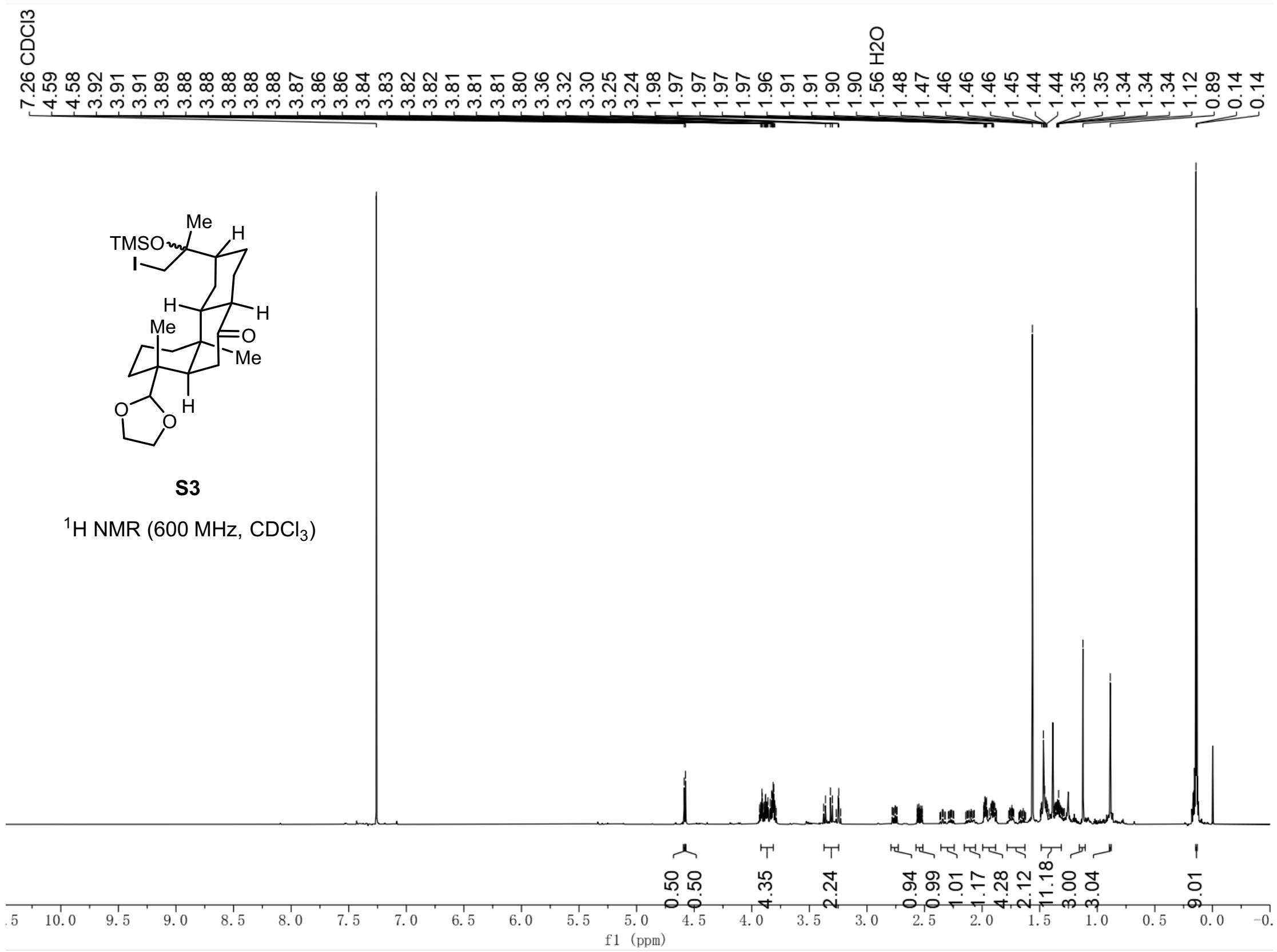

S3

^{1}H NMR (600 MHz, $CDCl_3$)

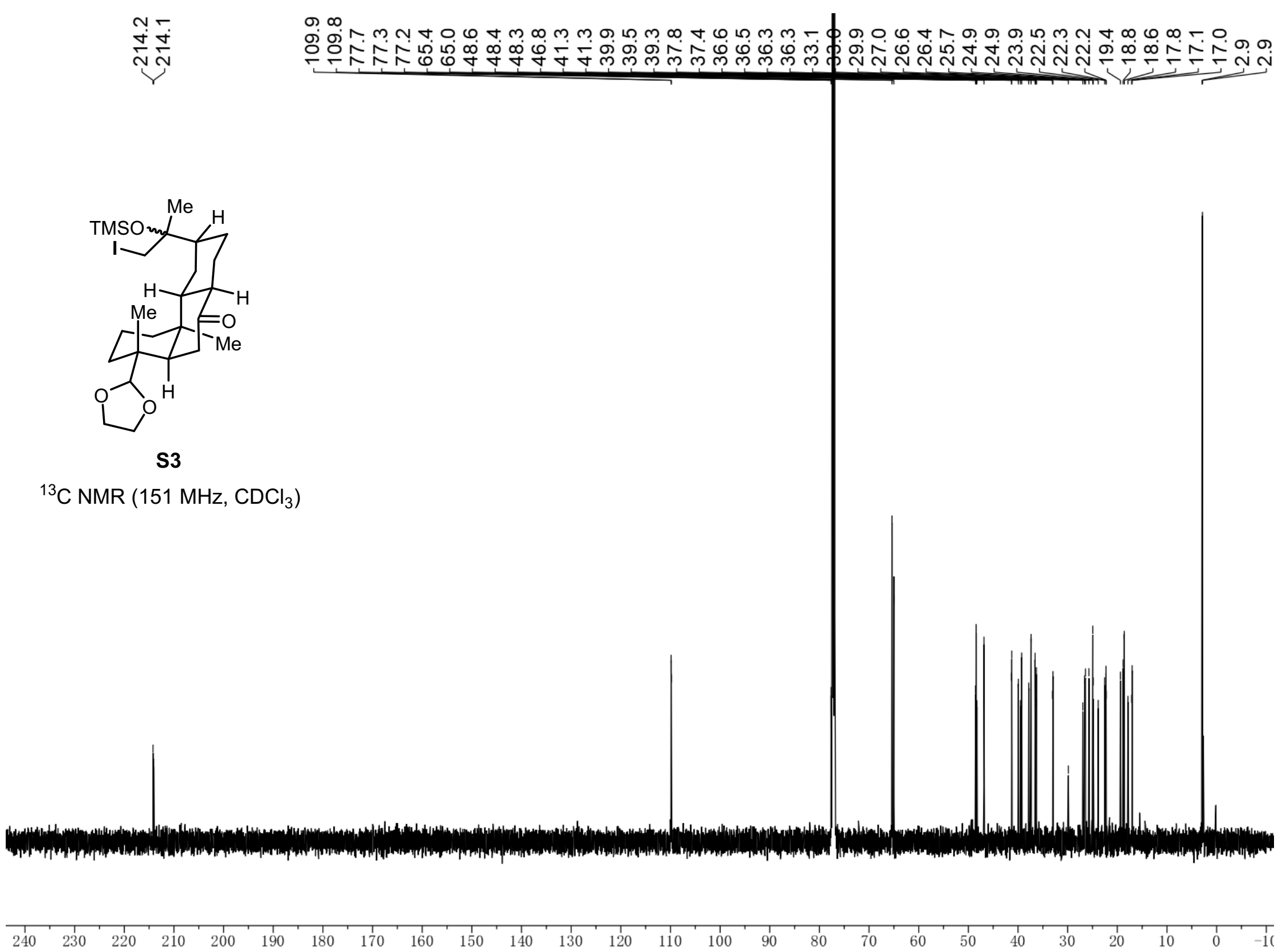

S3

^{13}C NMR (151 MHz, $CDCl_3$)

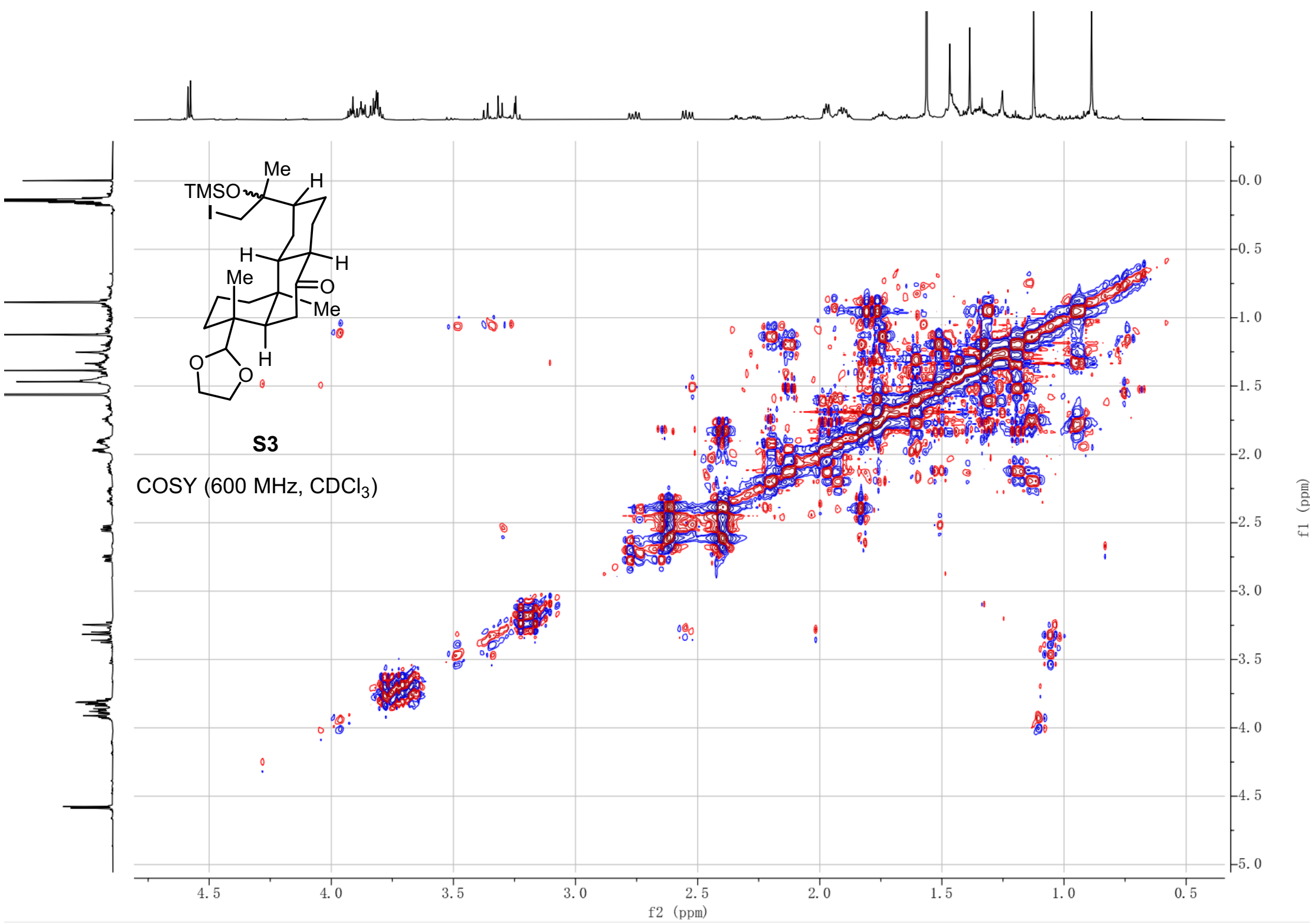

S3

COSY (600 MHz, $CDCl_3$)

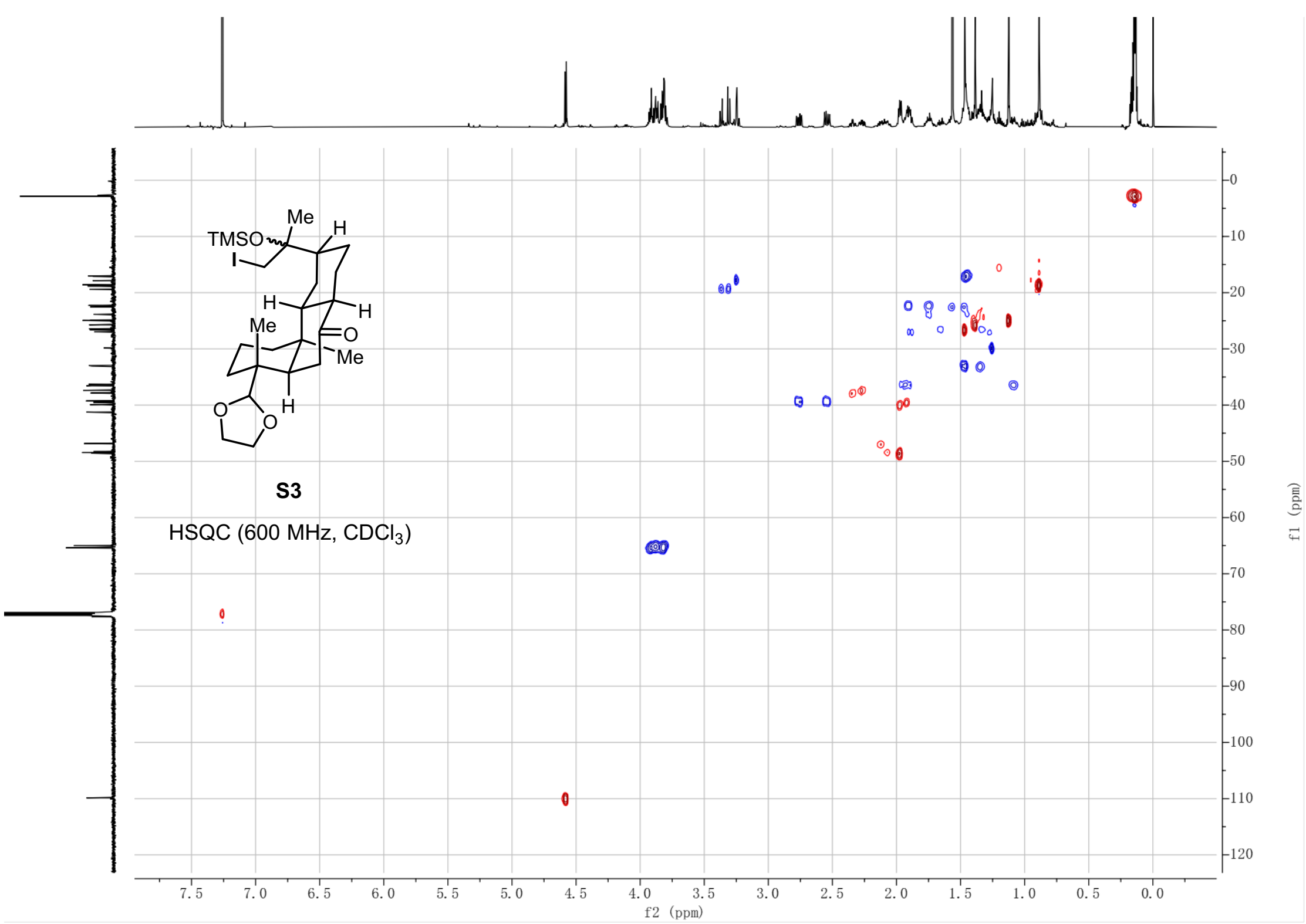

S3

HSQC (600 MHz, $CDCl_3$)

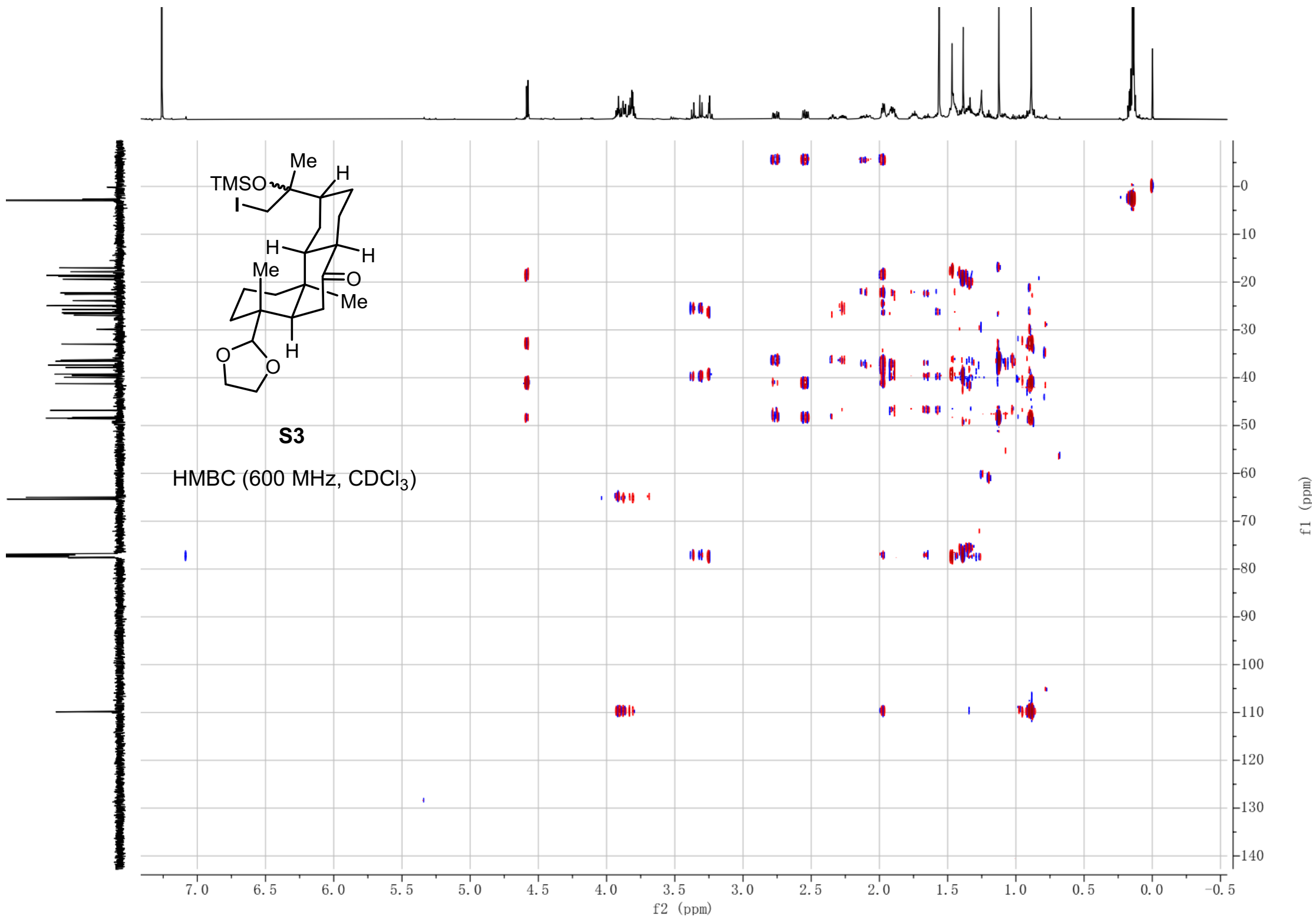
TMSO
Me
H
I
H
H
Me
O
Me
H
O
O
S3
HMBC (600 MHz, CDCl3)
f2 (ppm)
f1 (ppm)

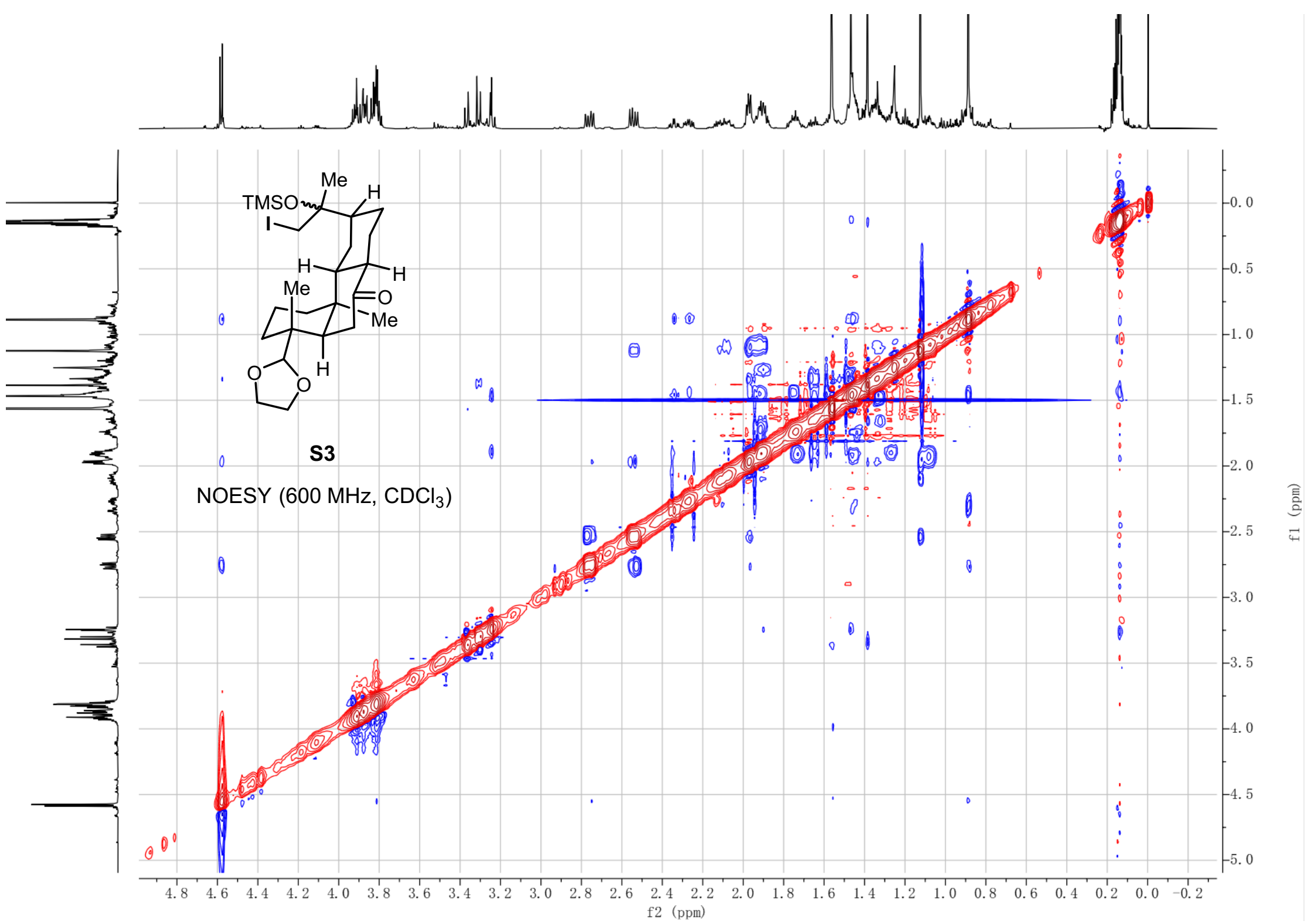
TMSO
Me
H
I
H
H
Me
O
Me
H
O
O
S3
NOESY (600 MHz, CDCl3)
f2 (ppm)
f1 (ppm)

23

^{1}H NMR (400 MHz, $CDCl_3$)

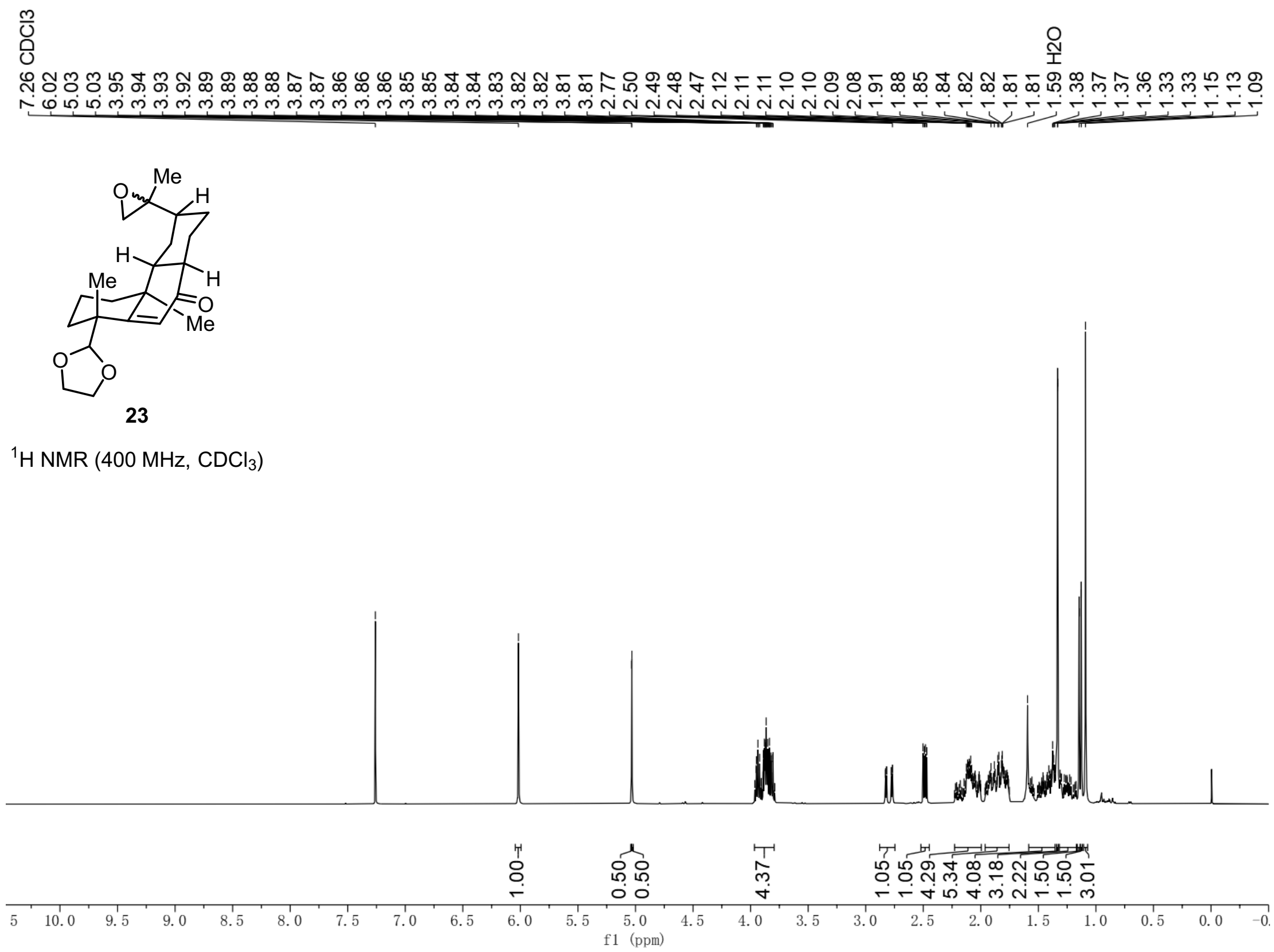

23

^{13}C NMR (101 MHz, $CDCl_3$)

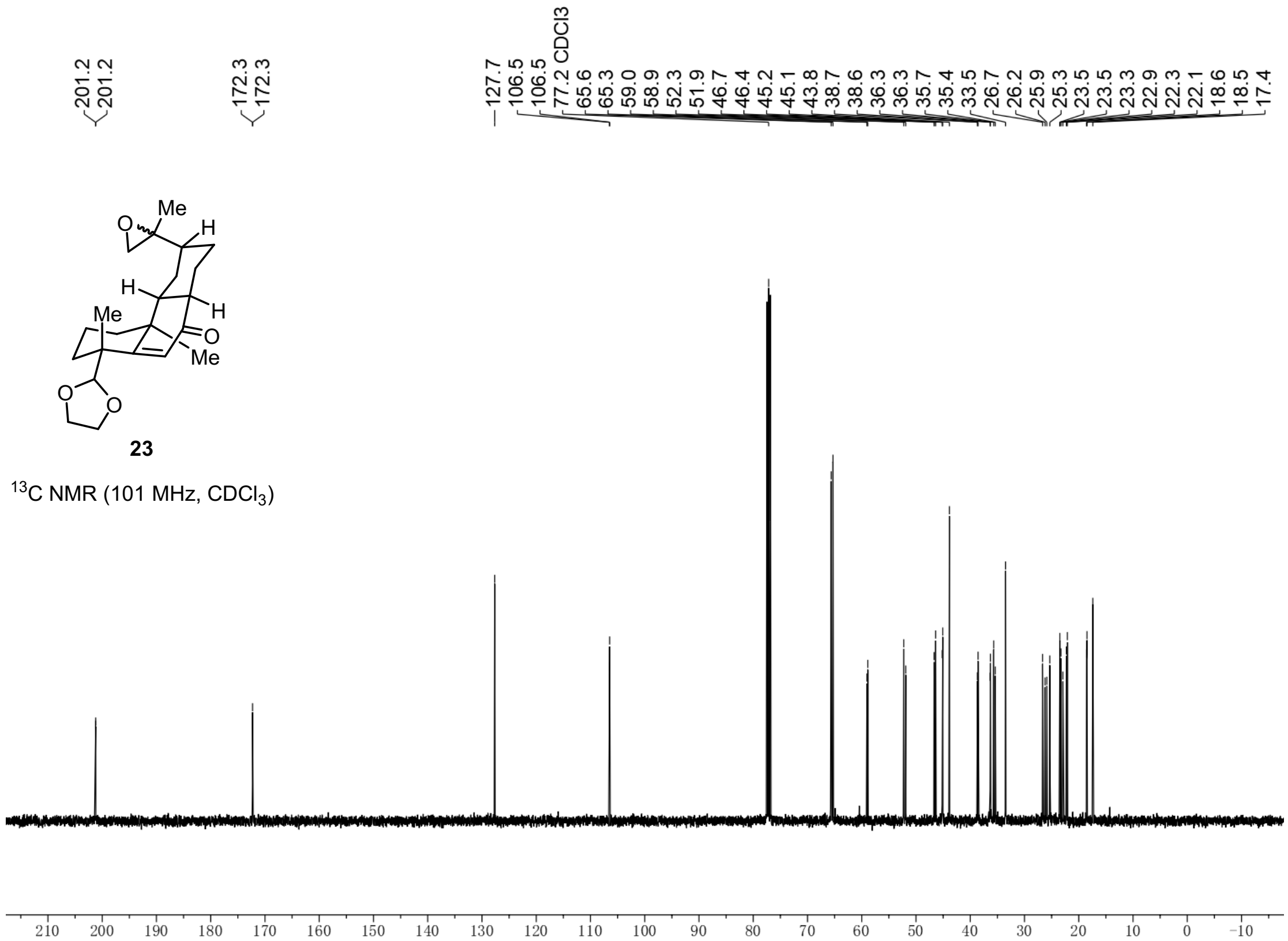

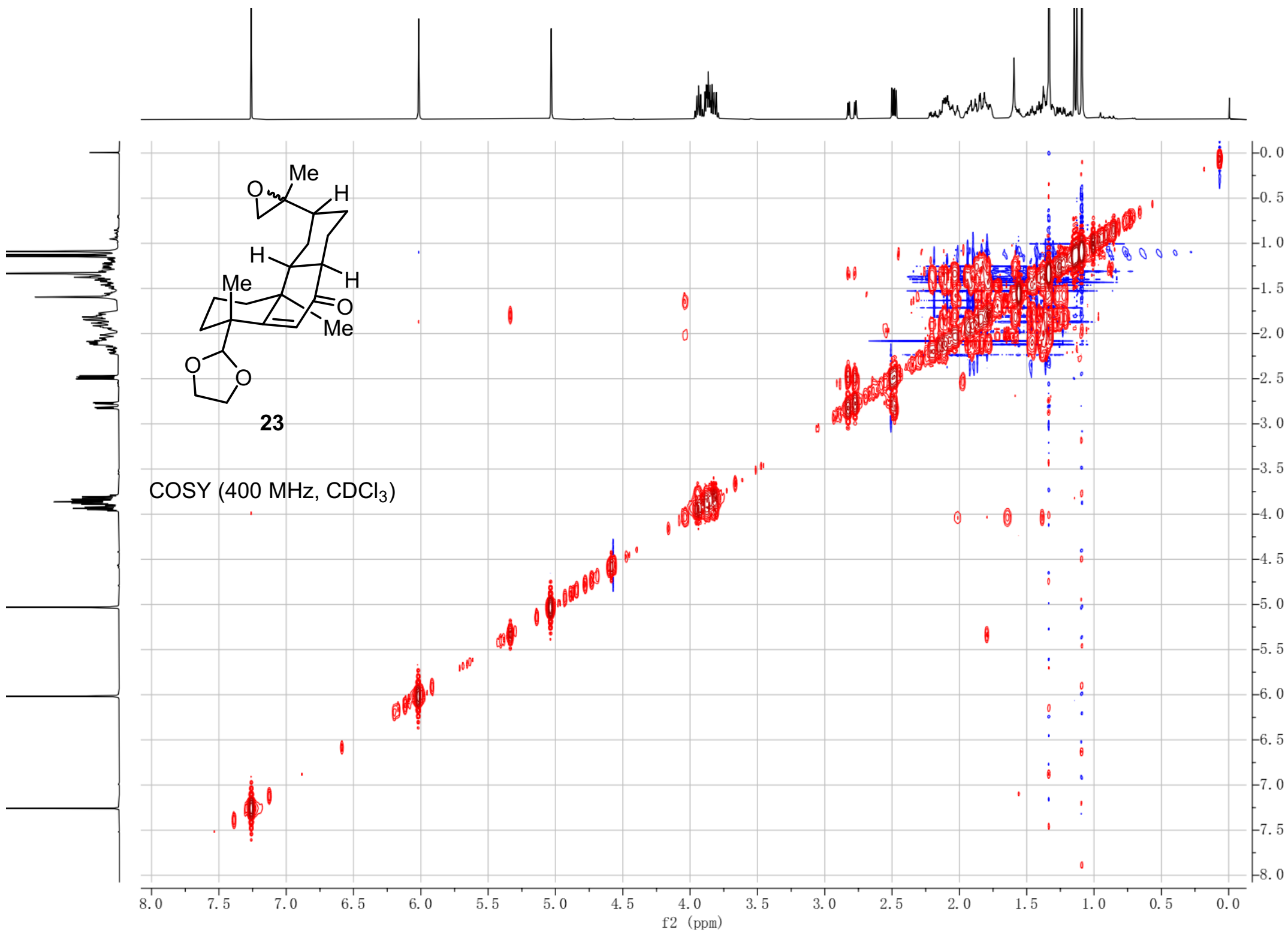
Me
H
H
H
Me
O
Me
O
O
O
23
COSY (400 MHz, CDCl3)
f2 (ppm)

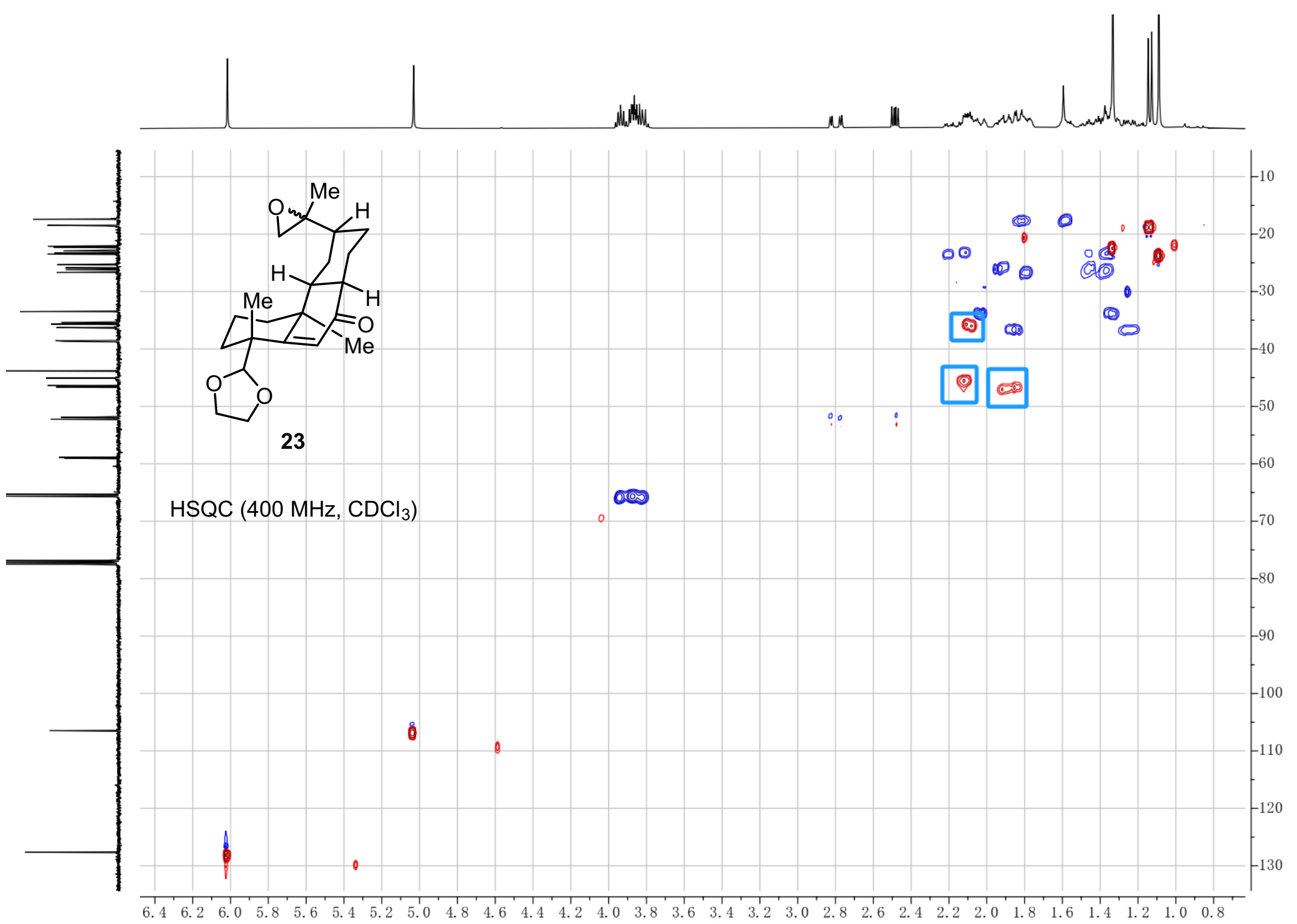
Me
H
H
H
Me
O
Me
O
O
O
23
HSQC (400 MHz, CDCl3)
f2 (ppm)

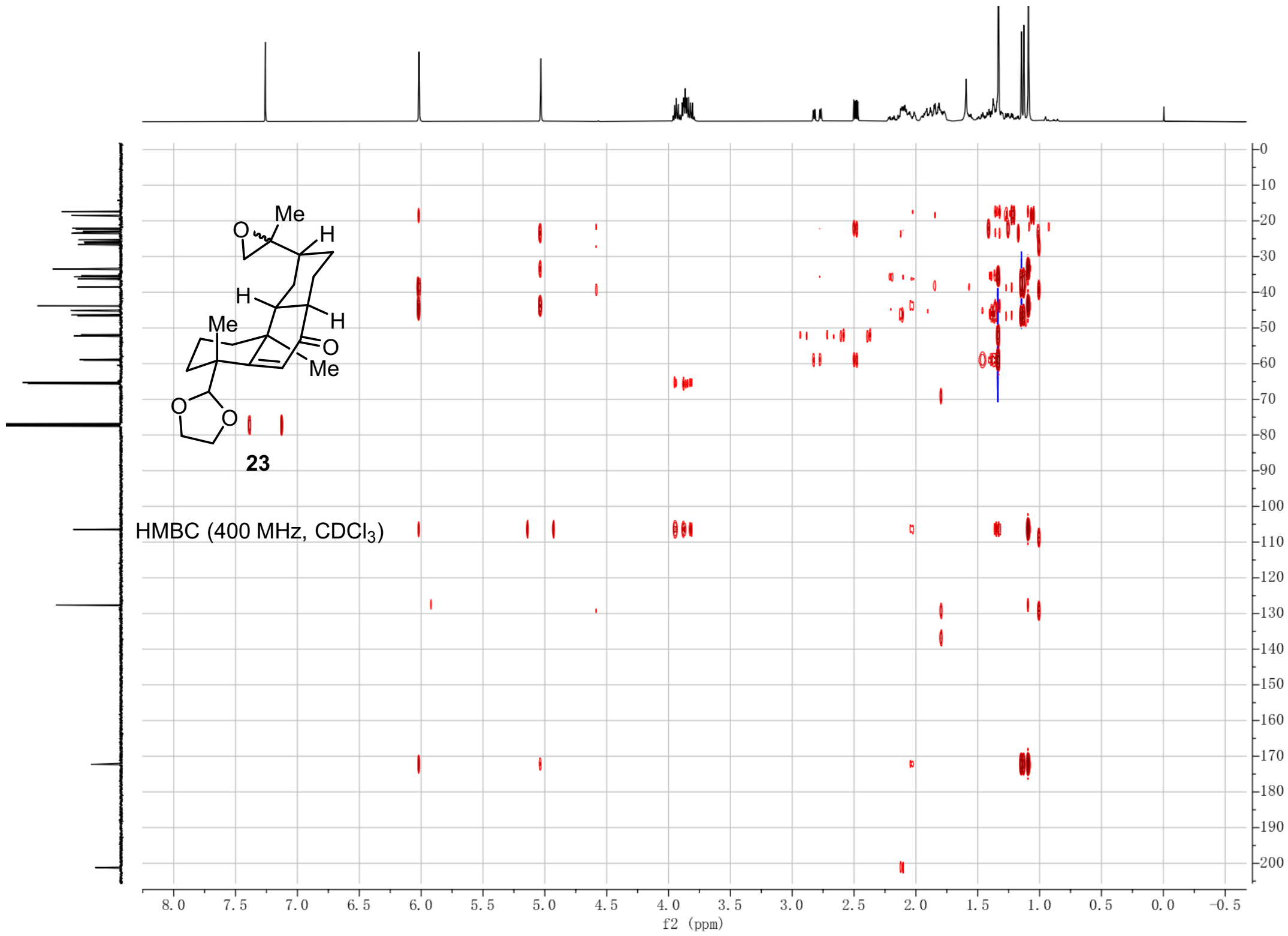
Me
H
H
H
Me
O
Me
O
O
O
23
HMBC (400 MHz, CDCl3)
f2 (ppm)

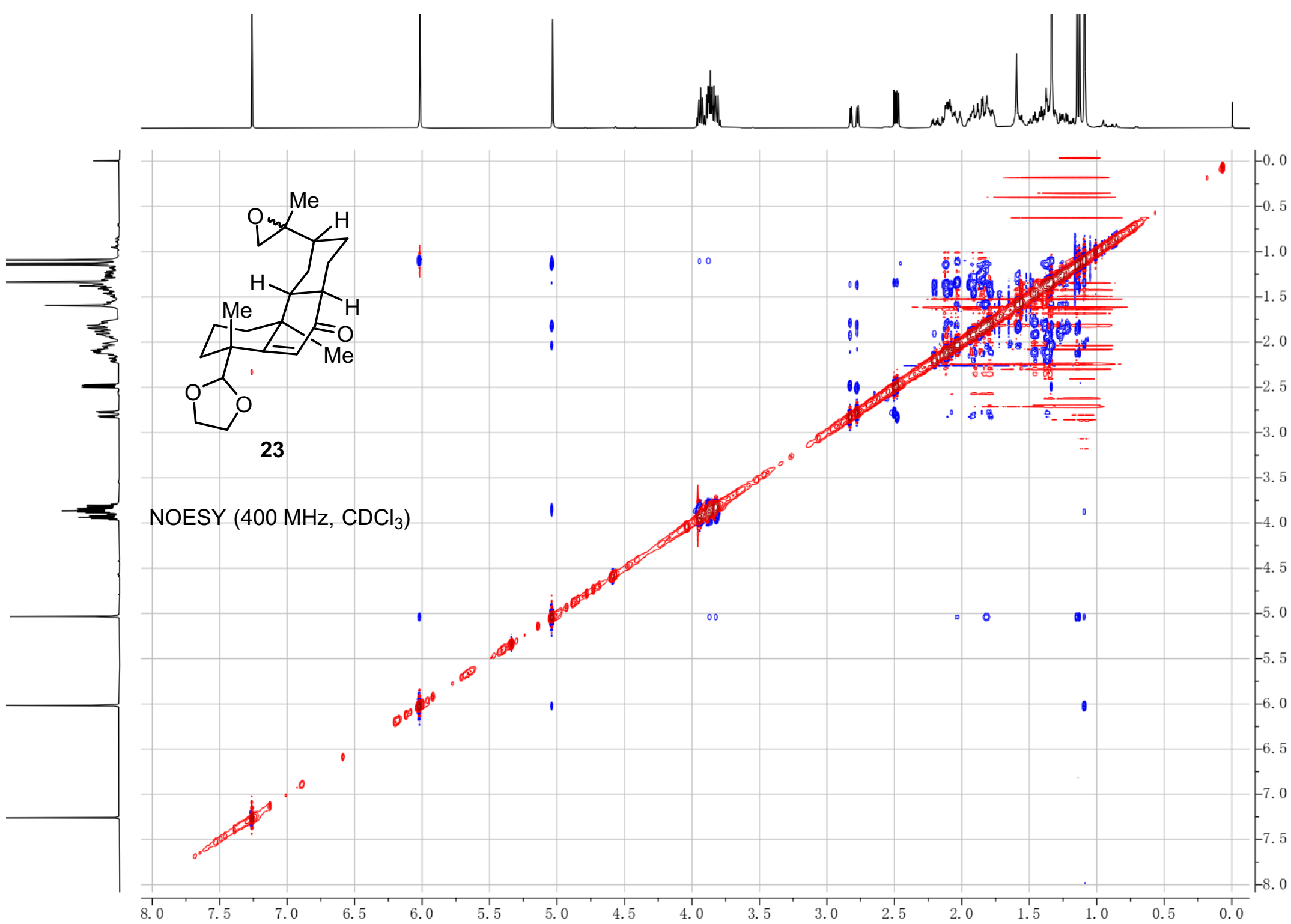
Me
H
H
H
Me
O
Me
O
O
O
23
NOESY (400 MHz, CDCl3)
f2 (ppm)

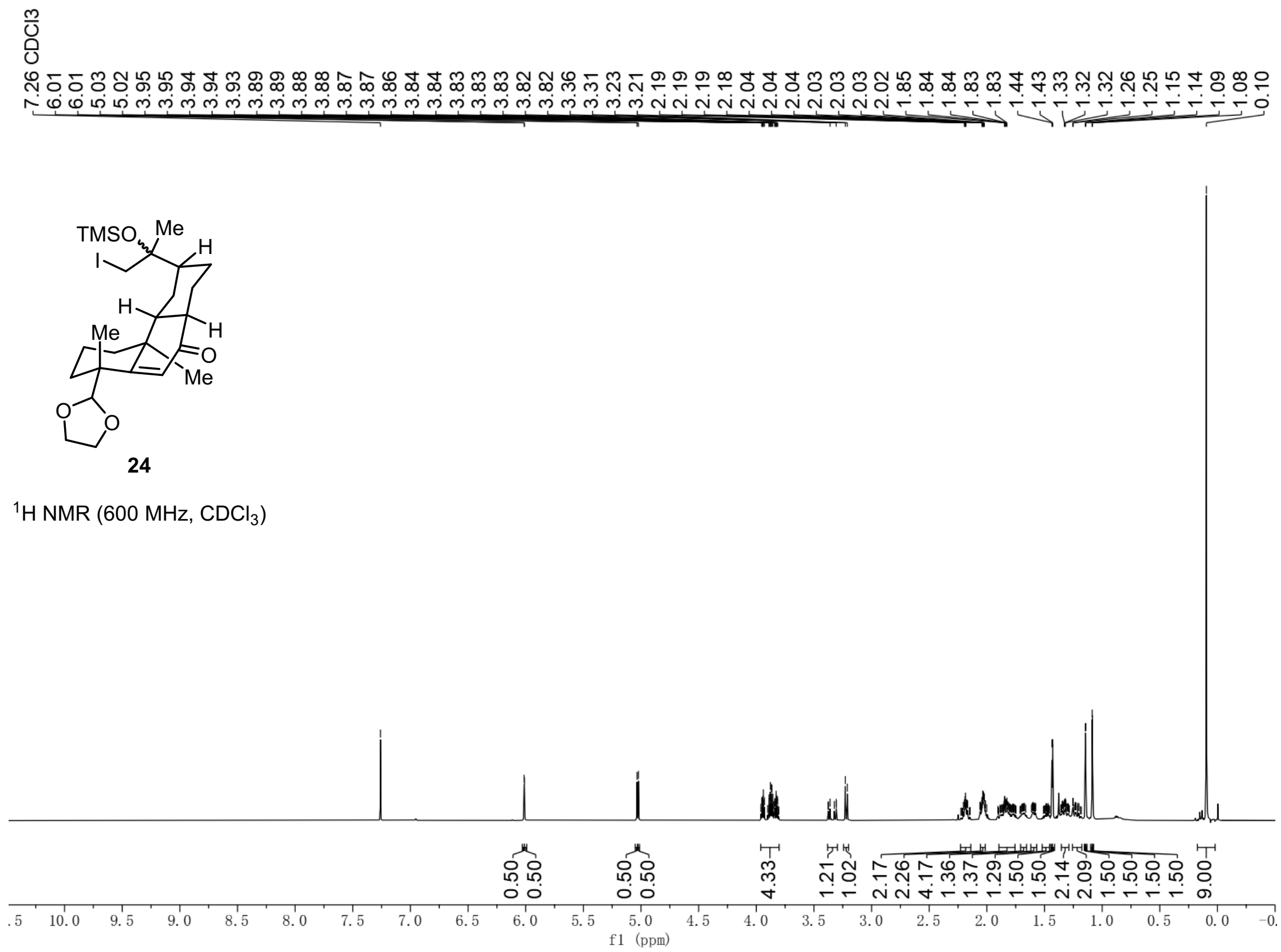
TMSO
Me
I
H
H
H
Me
O
Me
O
O
24
1H NMR (600 MHz, CDCl3)
f1 (ppm)

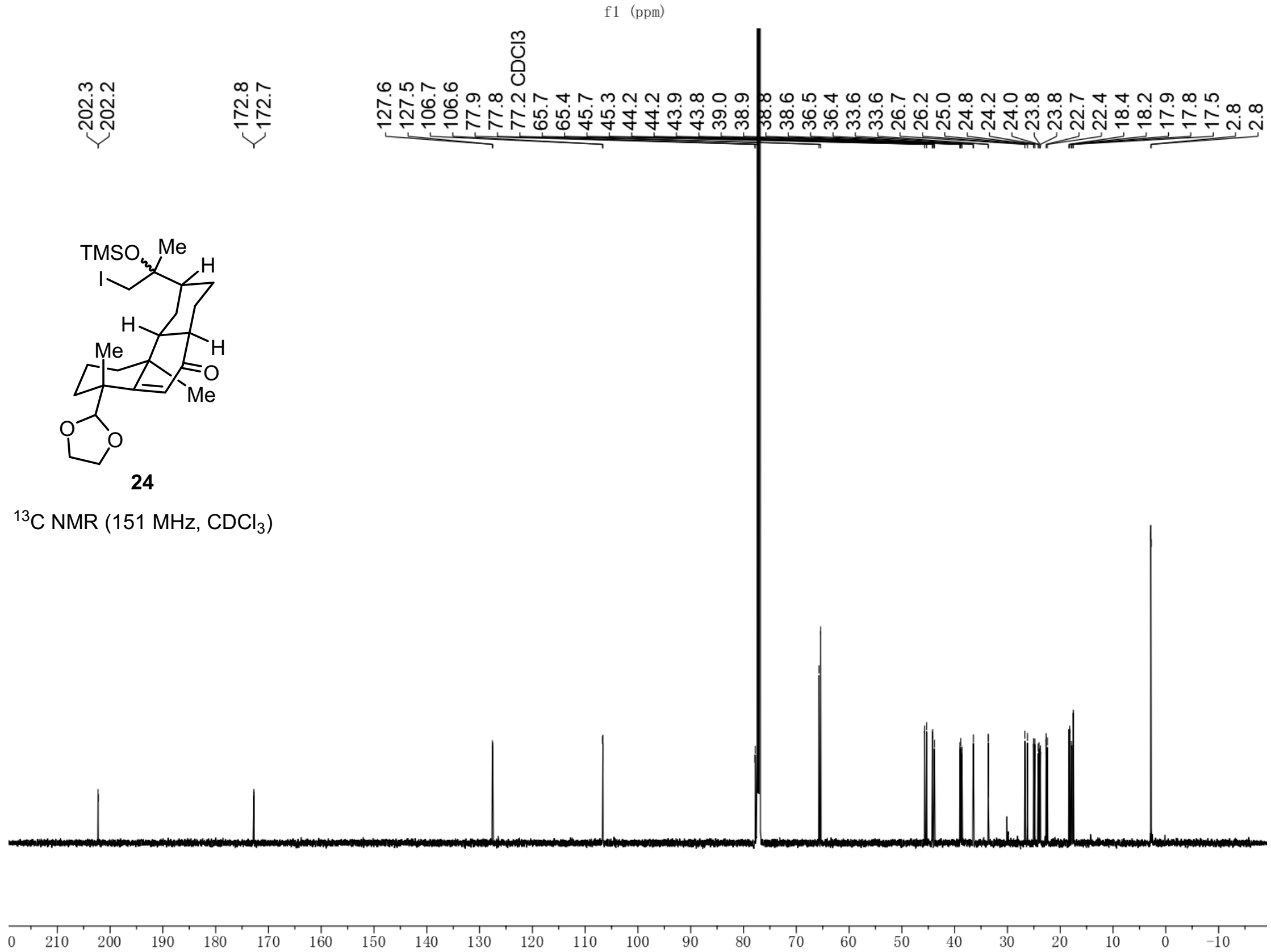
TMSO
Me
I
H
H
H
Me
O
Me
O
O
24
13C NMR (151 MHz, CDCl3)
f1 (ppm)

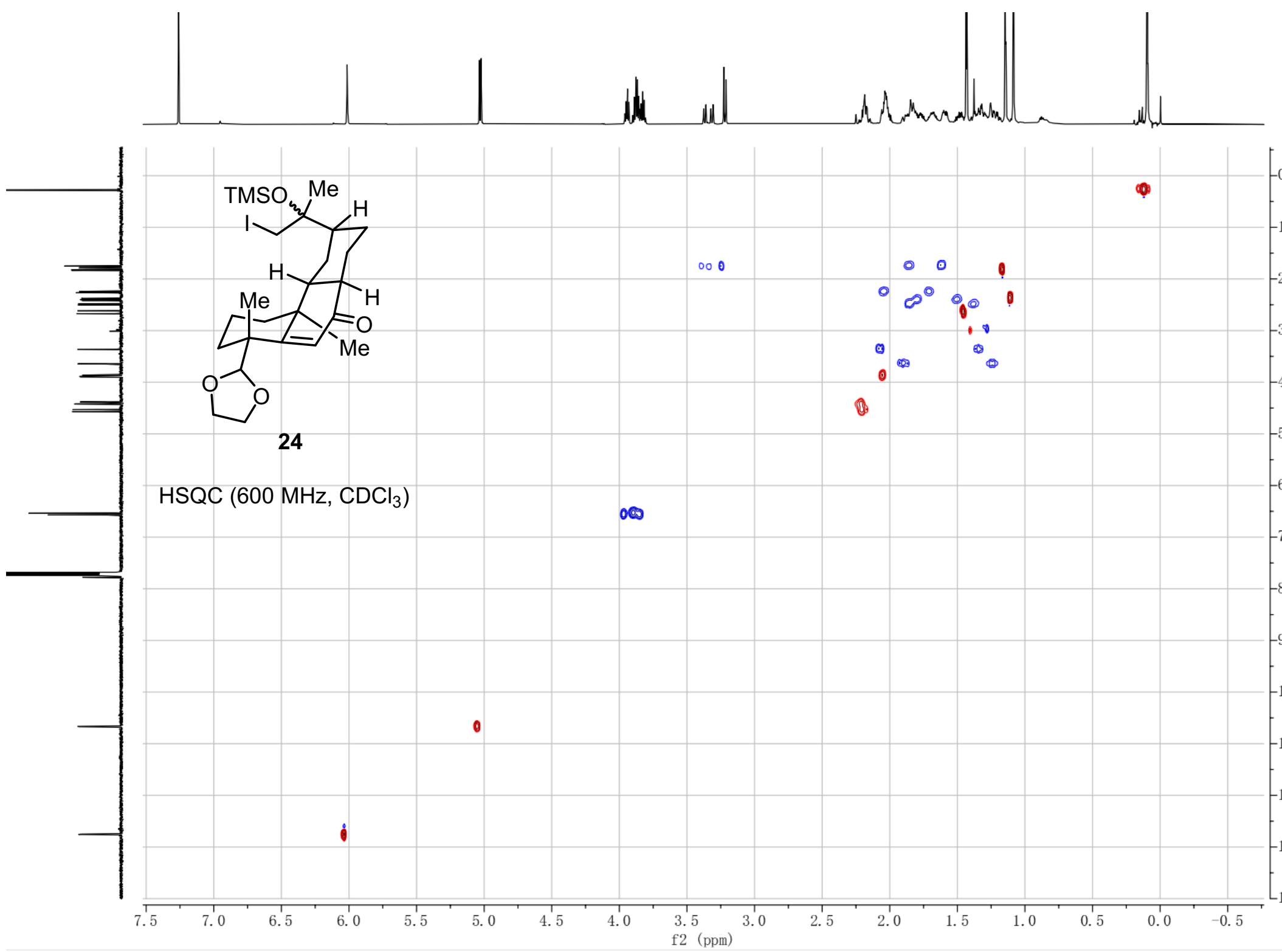
TMSO
Me
I
H
H
H
Me
O
Me
O
O
24
HSQC (600 MHz, CDCl3)
f2 (ppm)

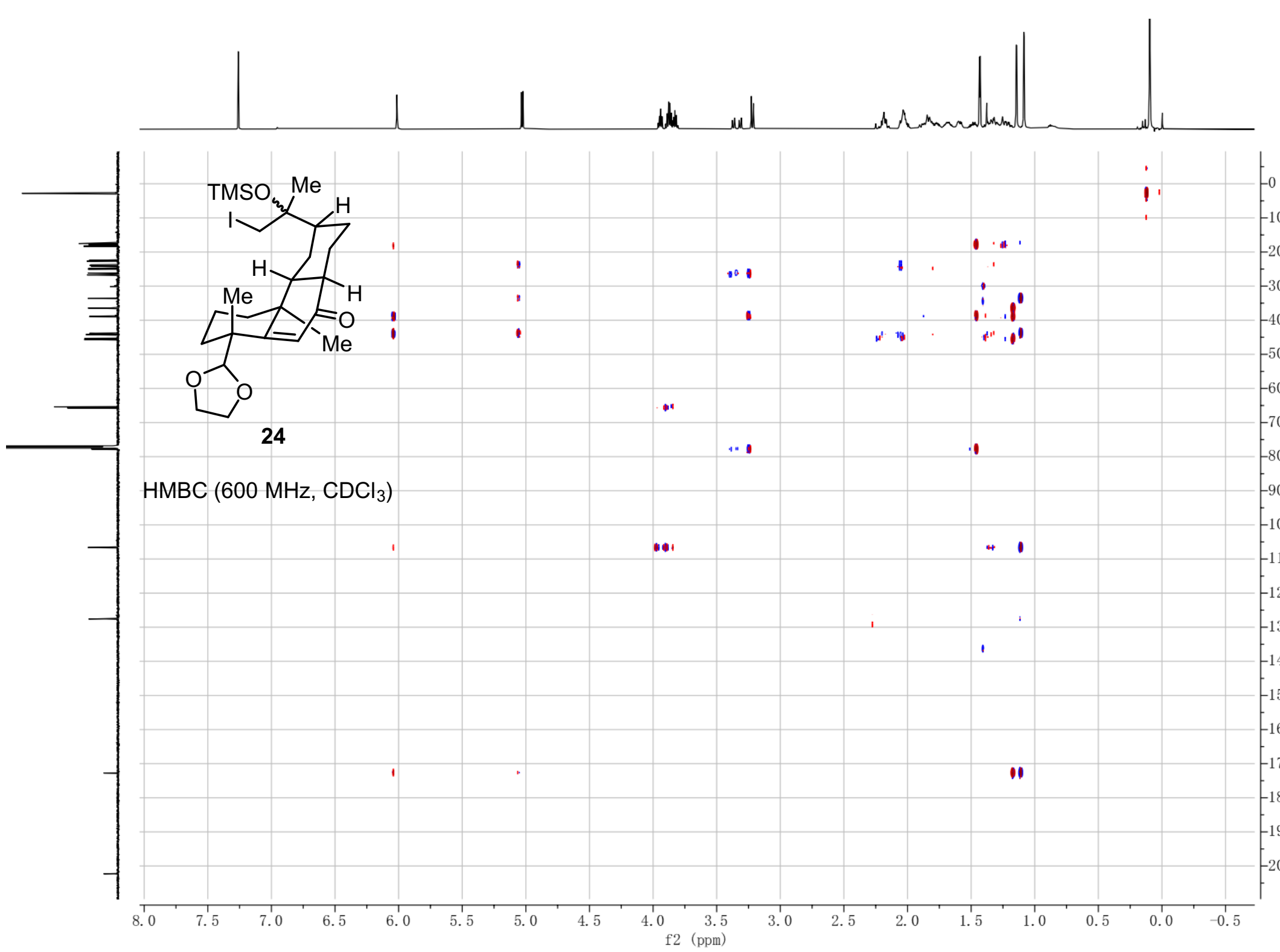
TMSO
Me
I
H
H
H
Me
O
Me
O
O
24
HMBC (600 MHz, CDCl3)
f2 (ppm)

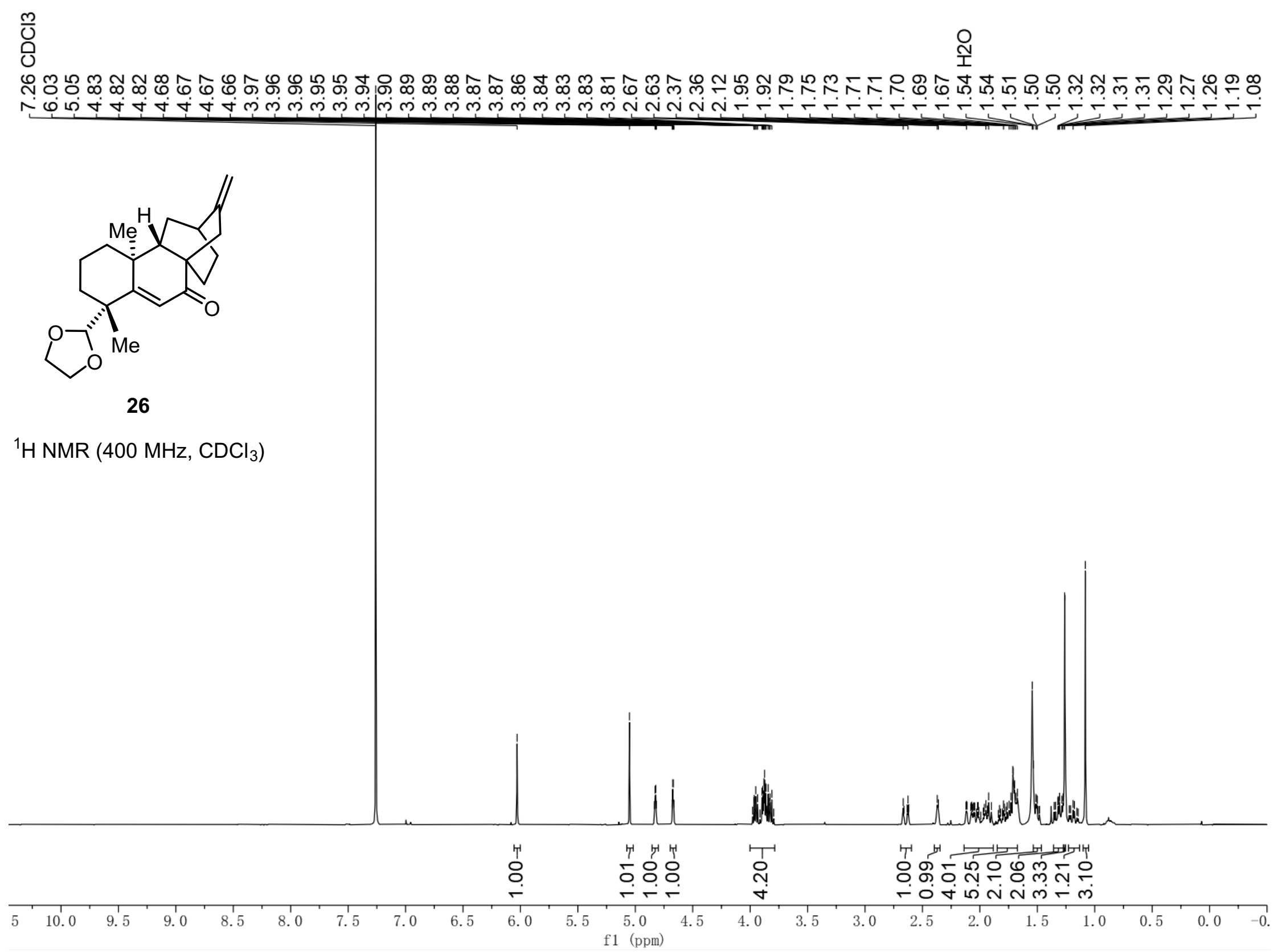

26

^{1}H NMR (400 MHz, $CDCl_3$)

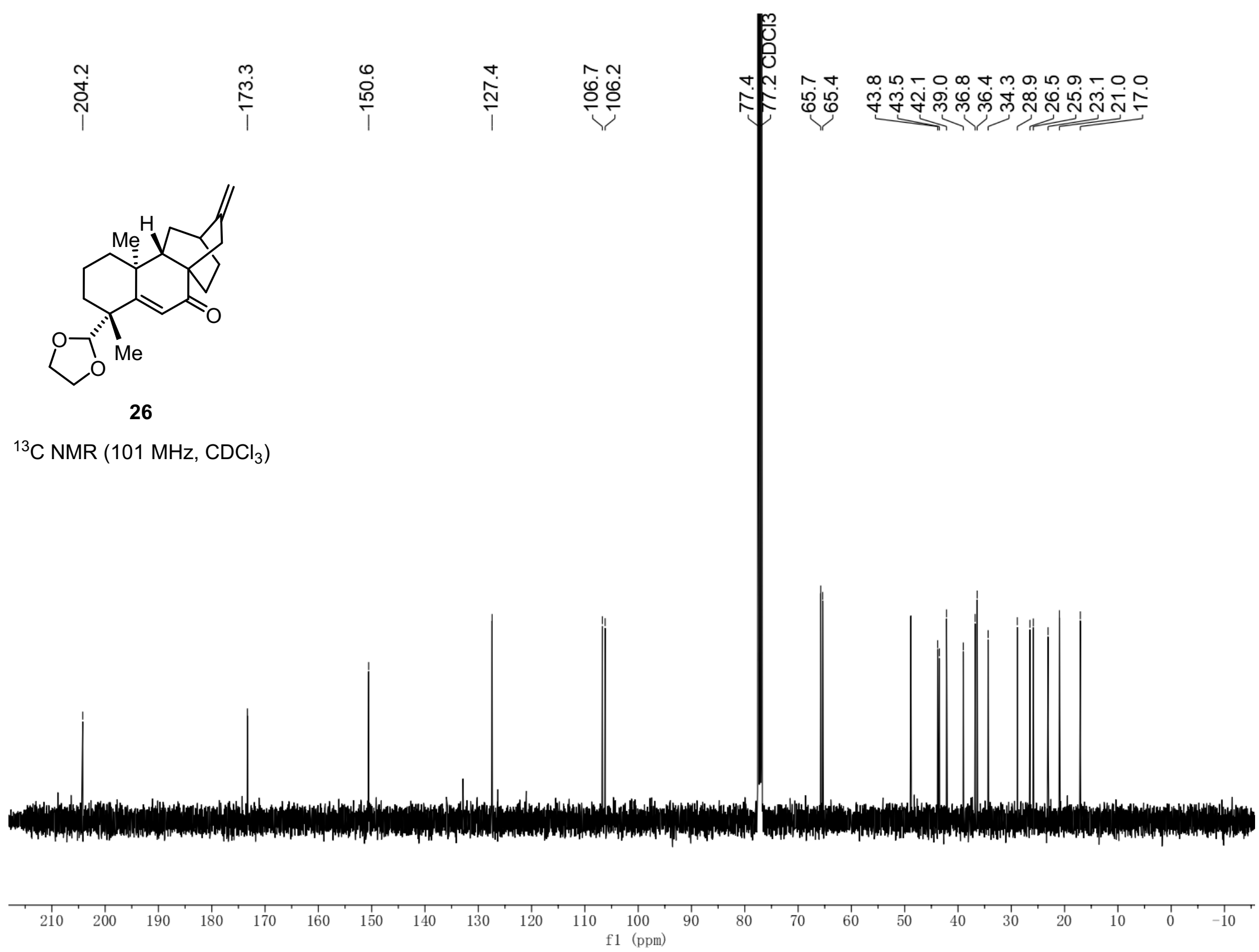

26

^{13}C NMR (101 MHz, $CDCl_3$)

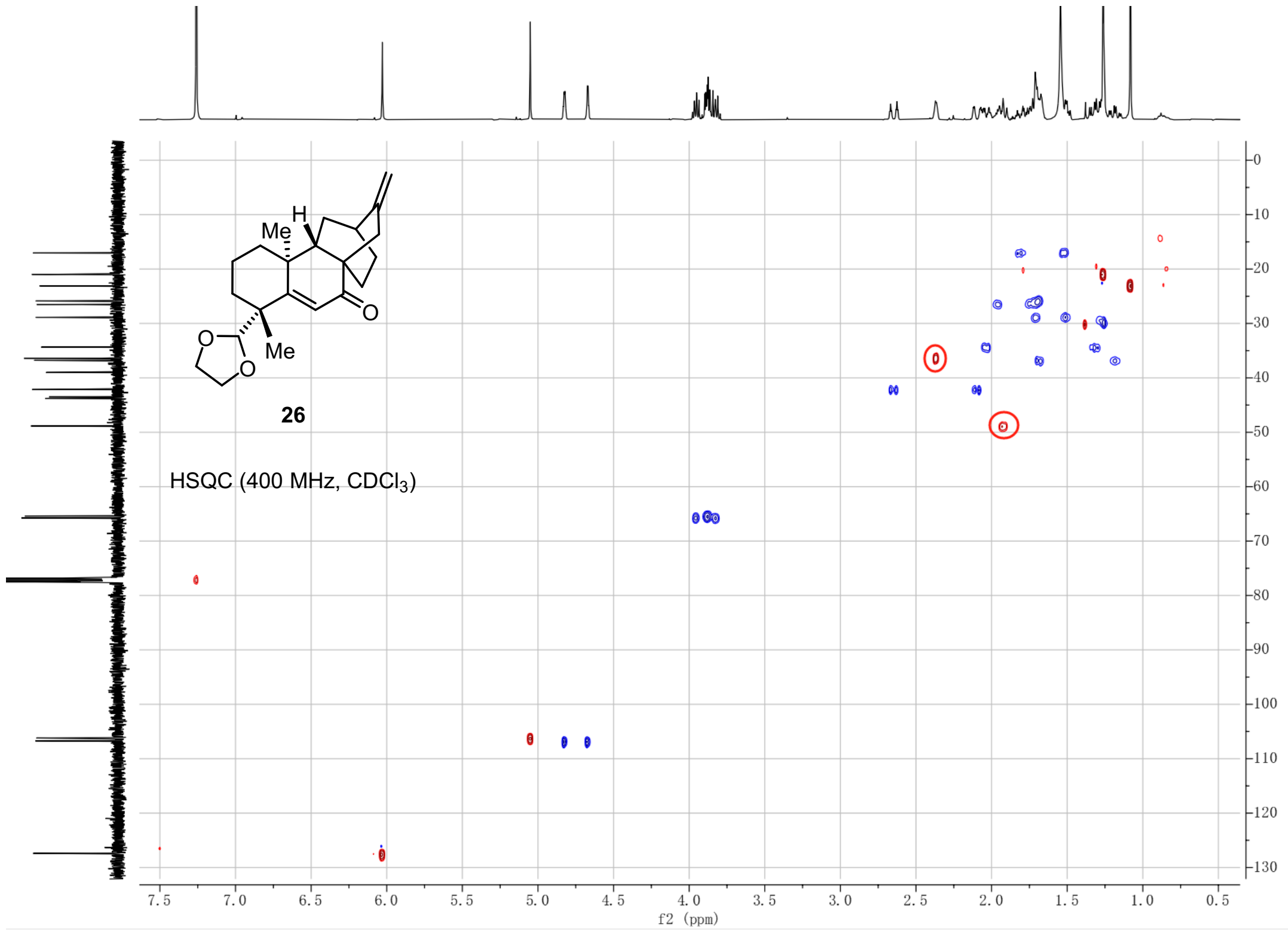
H
Me
O
Me
O
O
26
HSQC (400 MHz, CDCl3)
f2 (ppm)

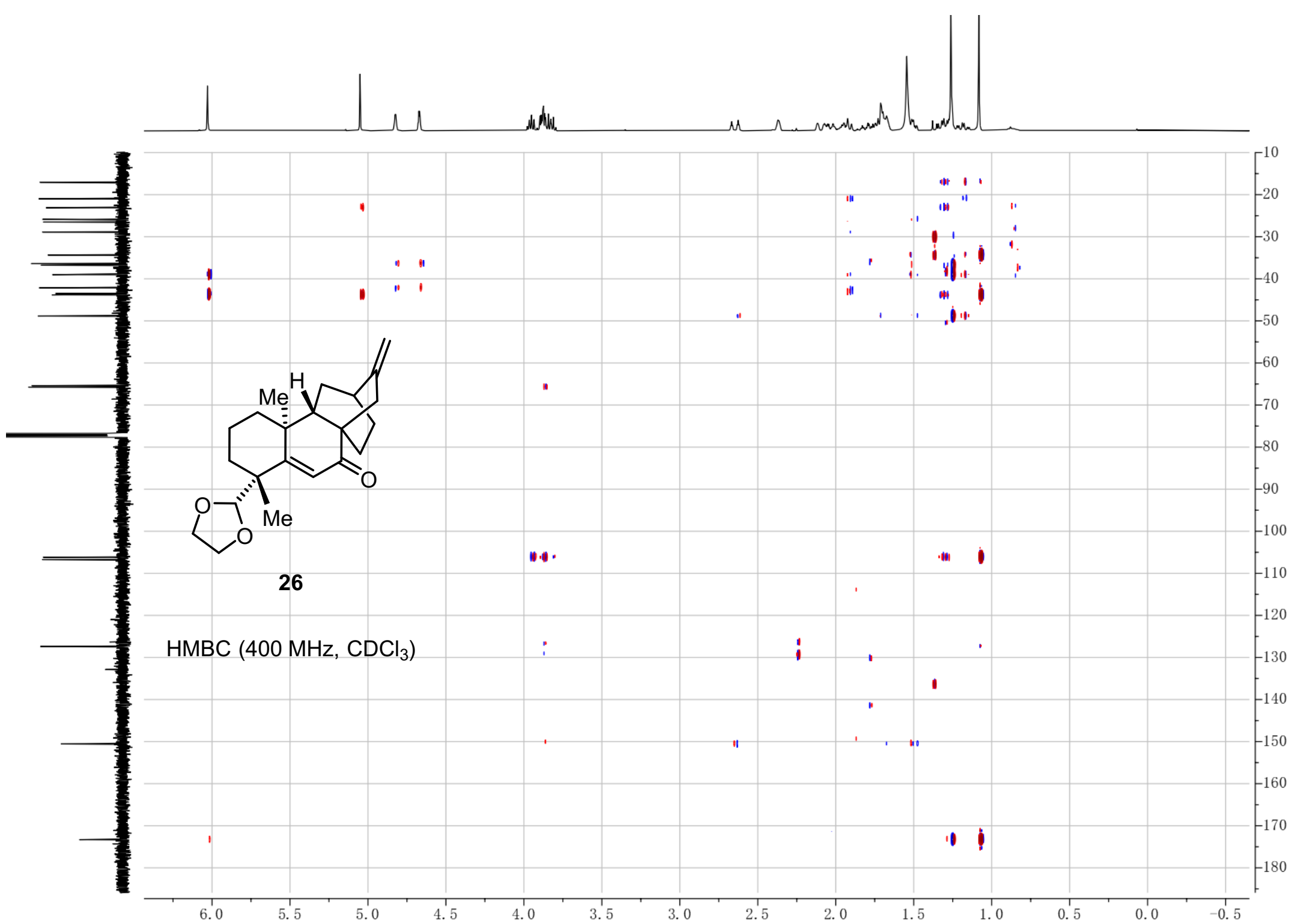
H
Me
O
Me
O
O
26
HMBC (400 MHz, CDCl3)
f2 (ppm)

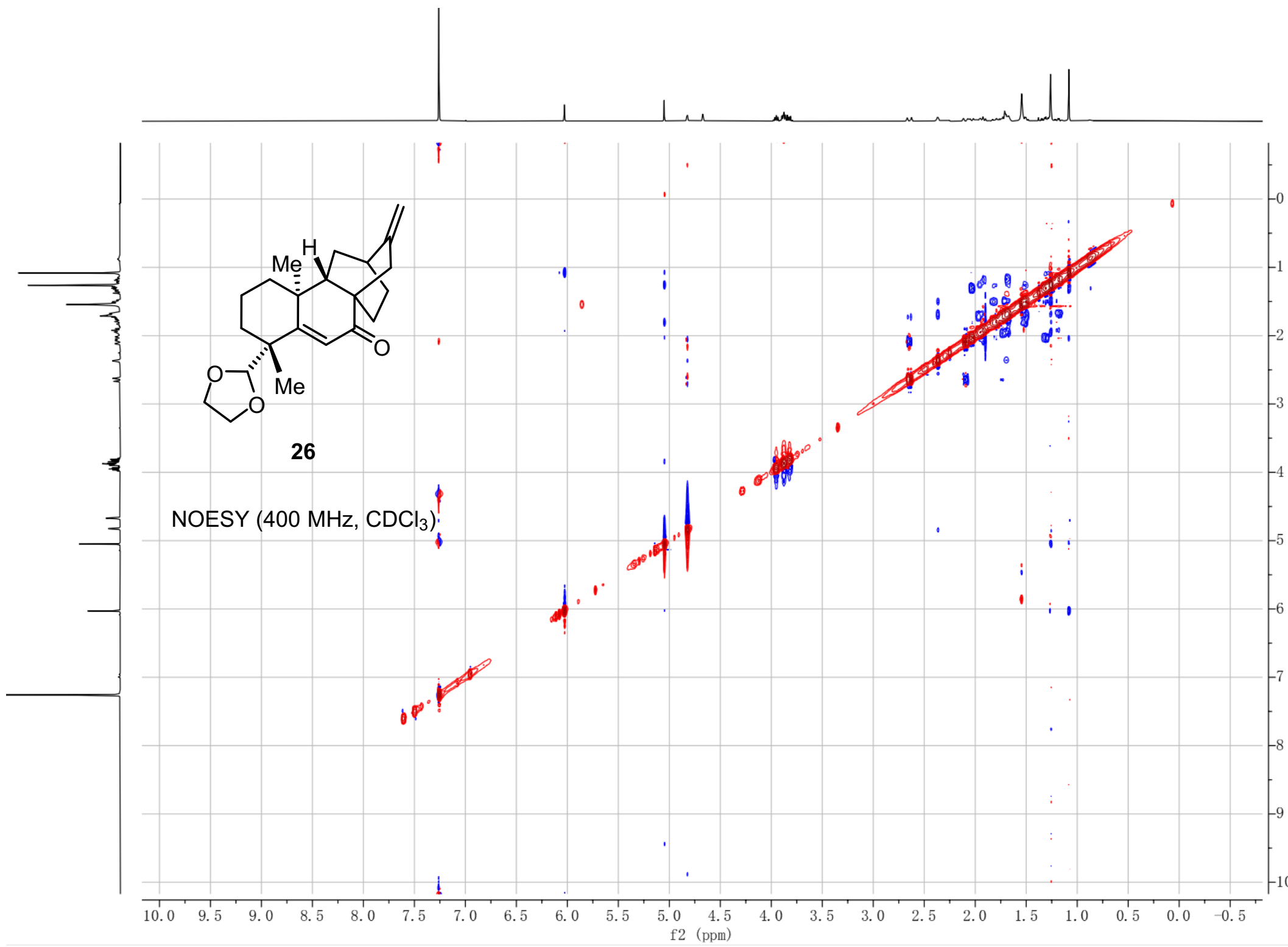

Me
H
O
O
Me
O
26
NOESY (400 MHz, CDCl3)
f2 (ppm)

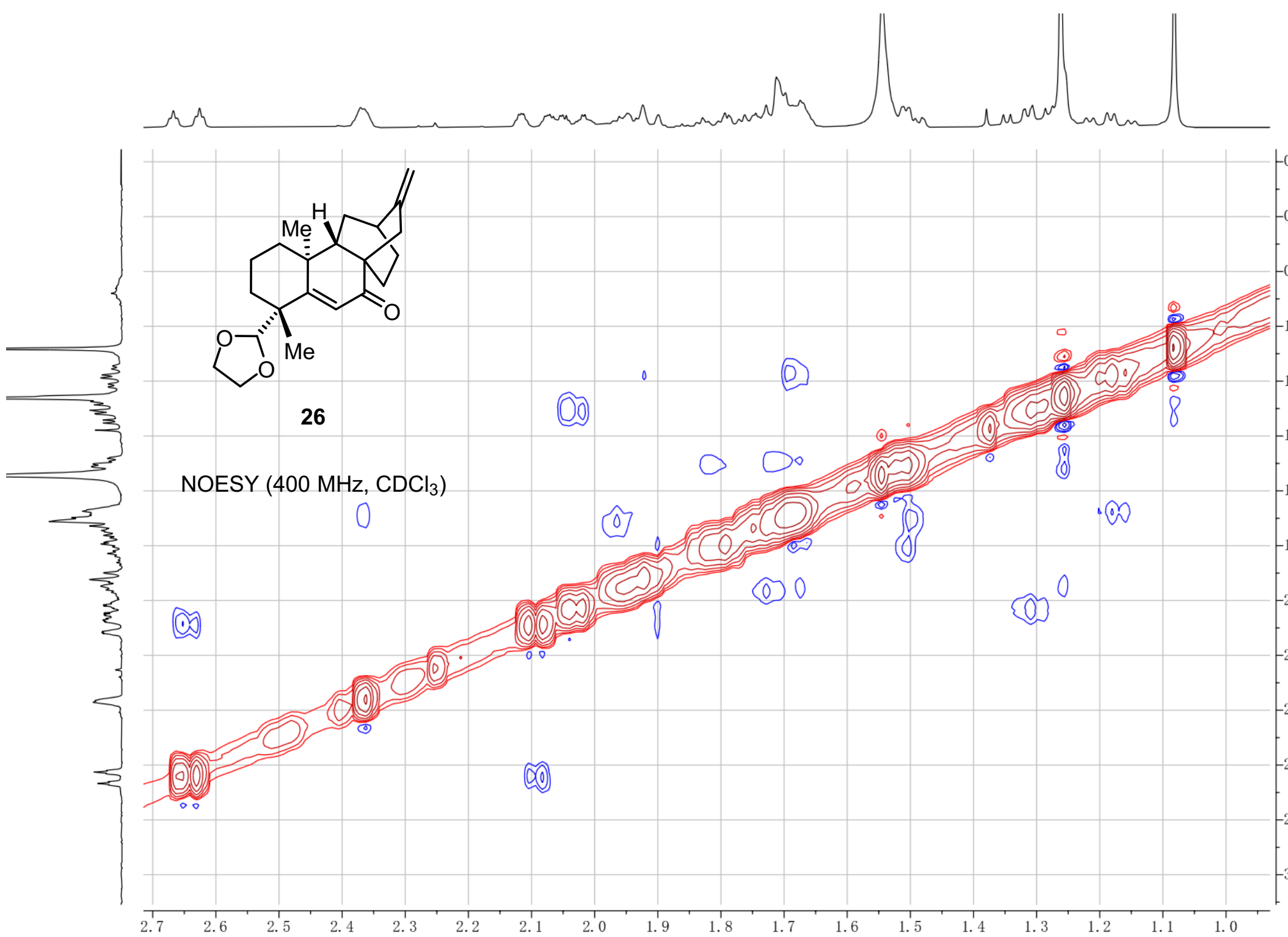

Me
H
O
O
Me
O
26
NOESY (400 MHz, CDCl3)
f2 (ppm)

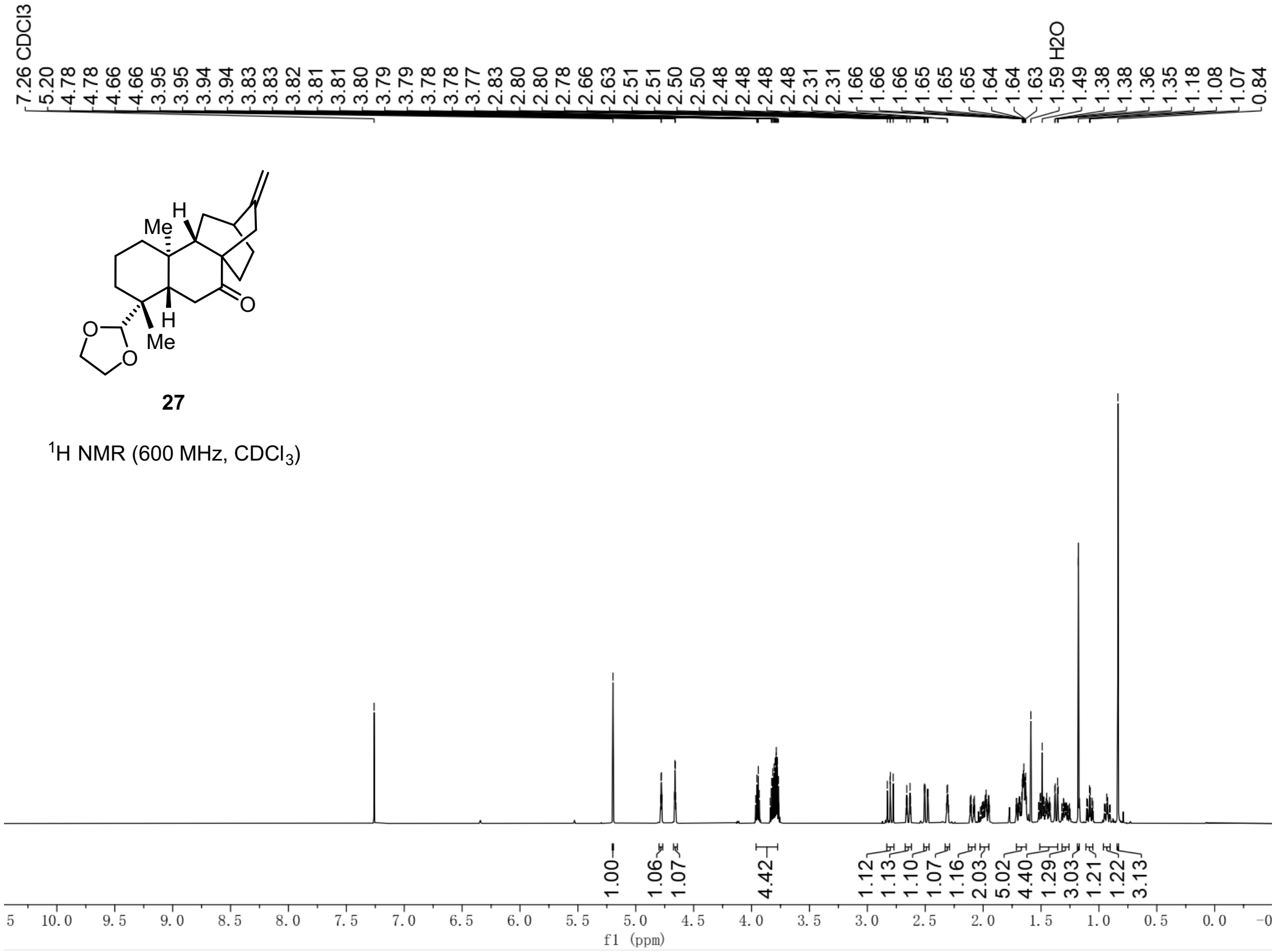

27

^{1}H NMR (600 MHz, $CDCl_3$)

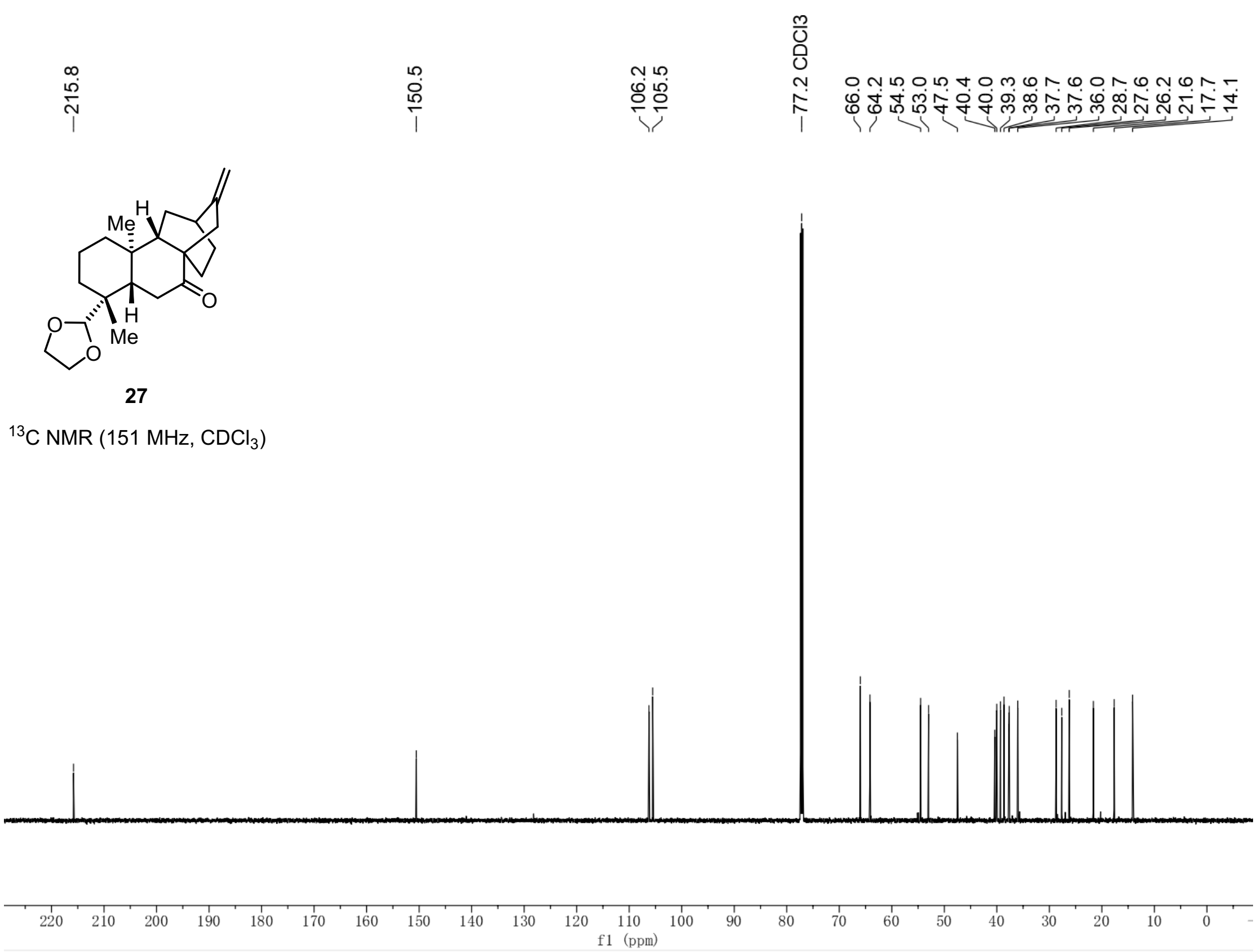

27

^{13}C NMR (151 MHz, $CDCl_3$)

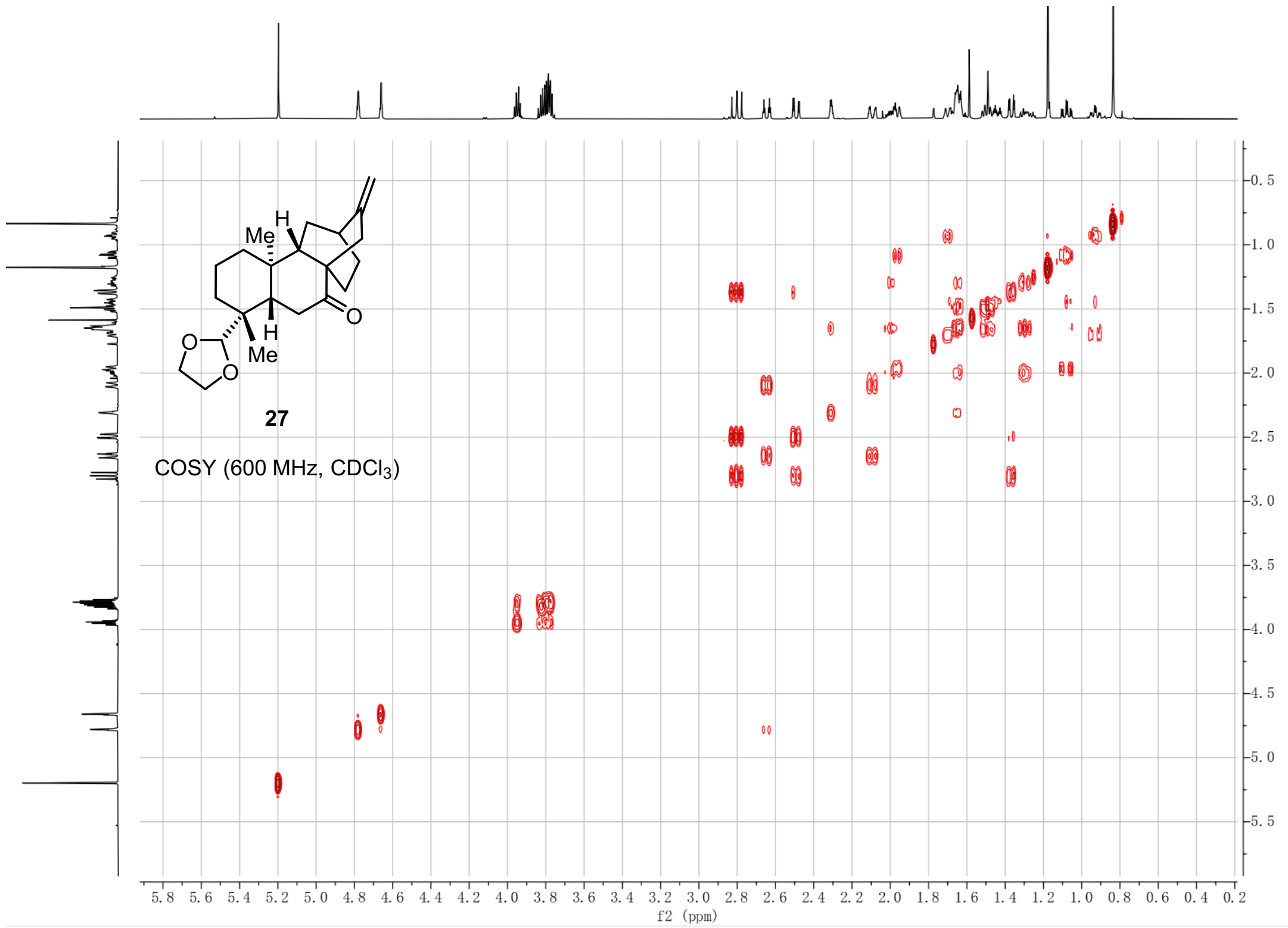

Me
H
O
H
Me
O
O
27
COSY (600 MHz, CDCl3)
f2 (ppm)

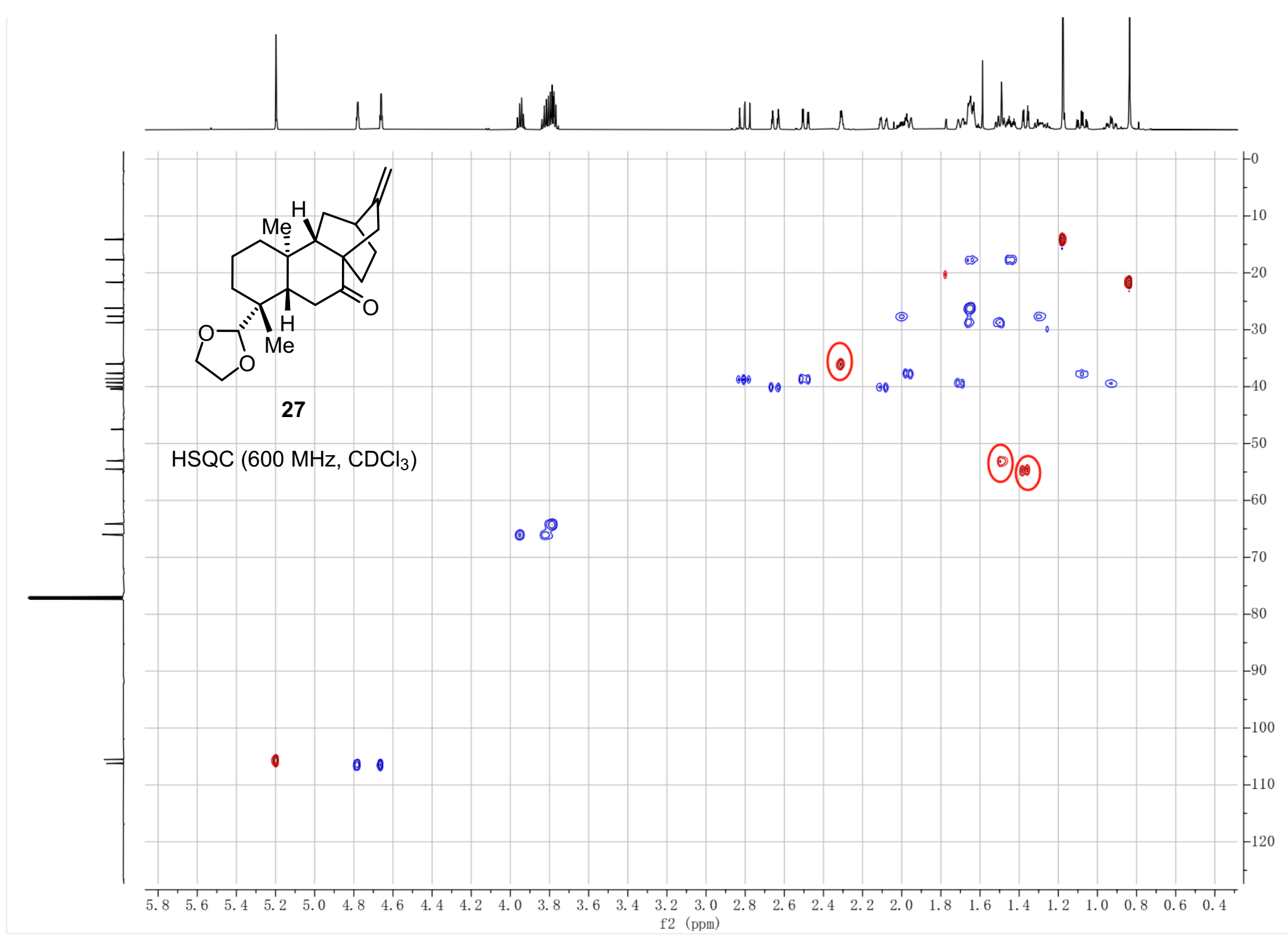

Me
H
O
H
Me
O
O
27
HSQC (600 MHz, CDCl3)
f2 (ppm)

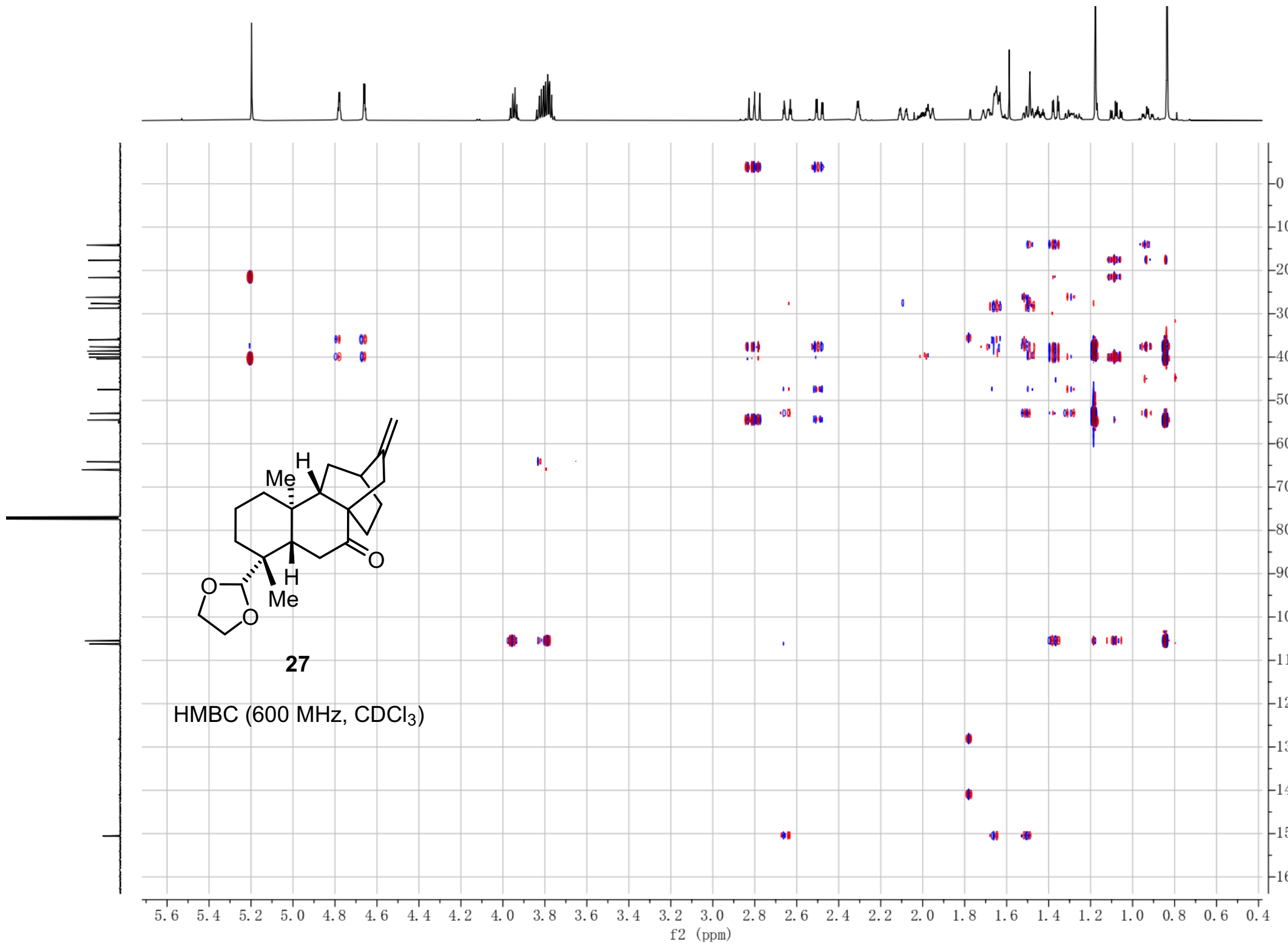

Me
H
H
Me
O
O
O
27
HMBC (600 MHz, CDCl$_3$)
f2 (ppm)

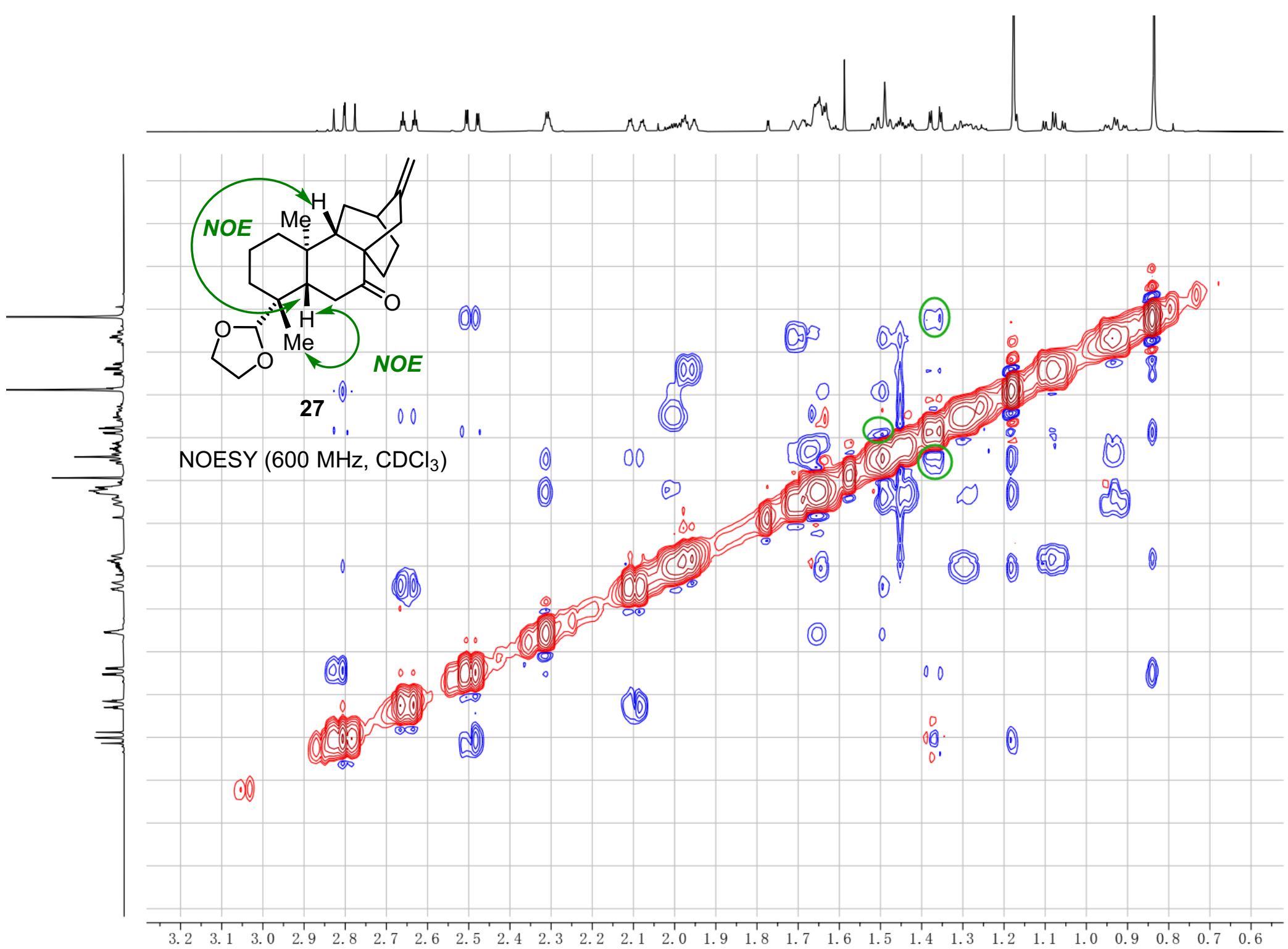

NOE
H
Me
H
Me
O
O
O
NOE
27
NOESY (600 MHz, CDCl$_3$)
f2 (ppm)

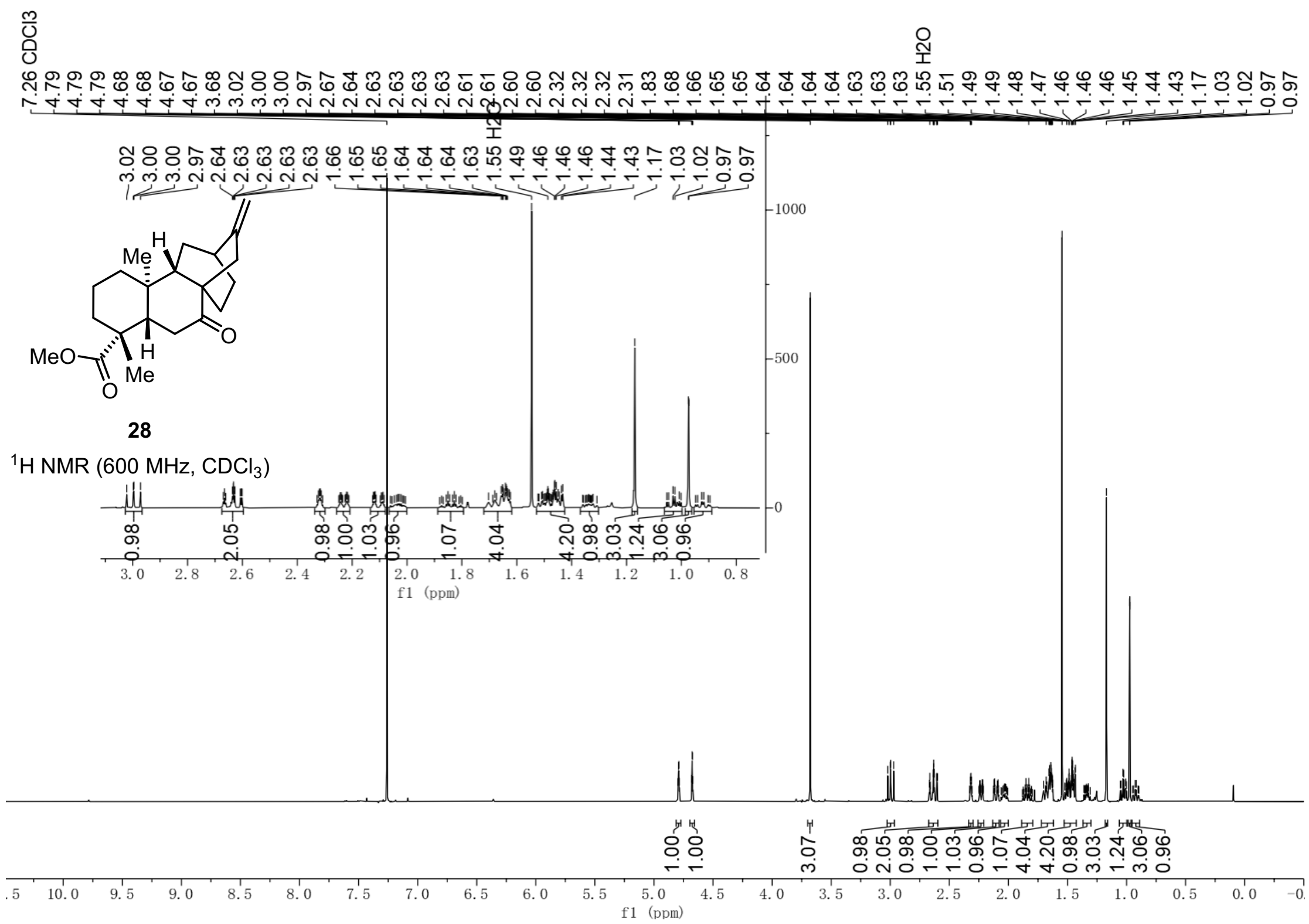

7.26 CDCl3
4.79
4.79
4.79
4.68
4.68
4.67
4.67
3.68
3.02
3.00
3.00
2.97
2.67
2.64
2.63
2.63
2.63
2.63
2.61
2.61
2.60
2.60
2.32
2.32
2.32
2.31
1.83
1.68
1.66
1.65
1.65
1.64
1.64
1.64
1.64
1.63
1.63
1.63
1.55 H2O
1.51
1.49
1.49
1.48
1.47
1.46
1.46
1.46
1.45
1.44
1.43
1.17
1.03
1.02
0.97
0.97
Me
H
MeO
O
H
Me
O
28
^{1}H NMR (600 MHz, $CDCl_3$)
f1 (ppm)

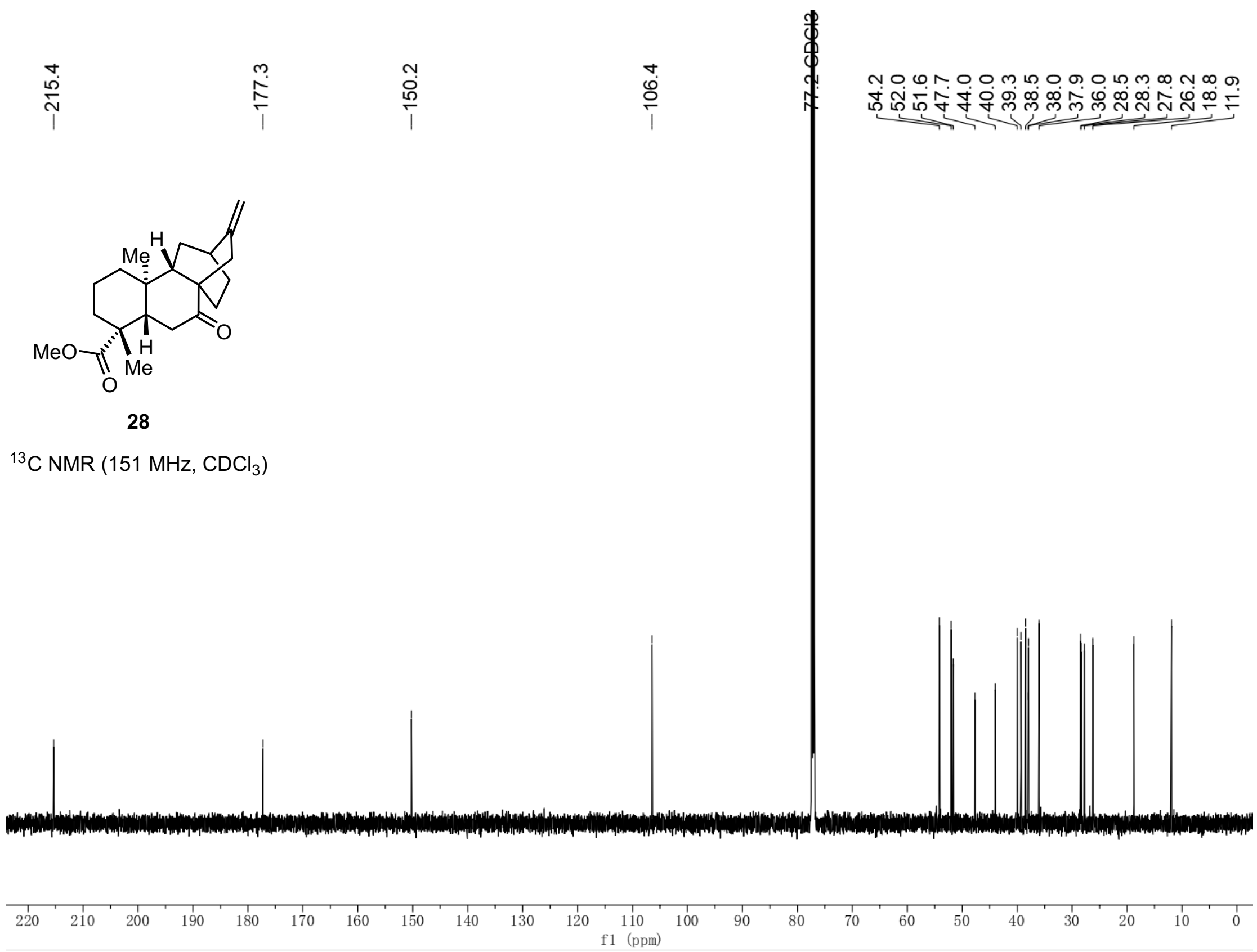

215.4
177.3
150.2
106.4
77.2 CDCl3
54.2
52.0
51.6
47.7
44.0
40.0
39.3
38.5
38.0
37.9
36.0
28.5
28.3
27.8
26.2
18.8
11.9
Me
H
MeO
O
H
Me
O
28
^{13}C NMR (151 MHz, $CDCl_3$)
f1 (ppm)

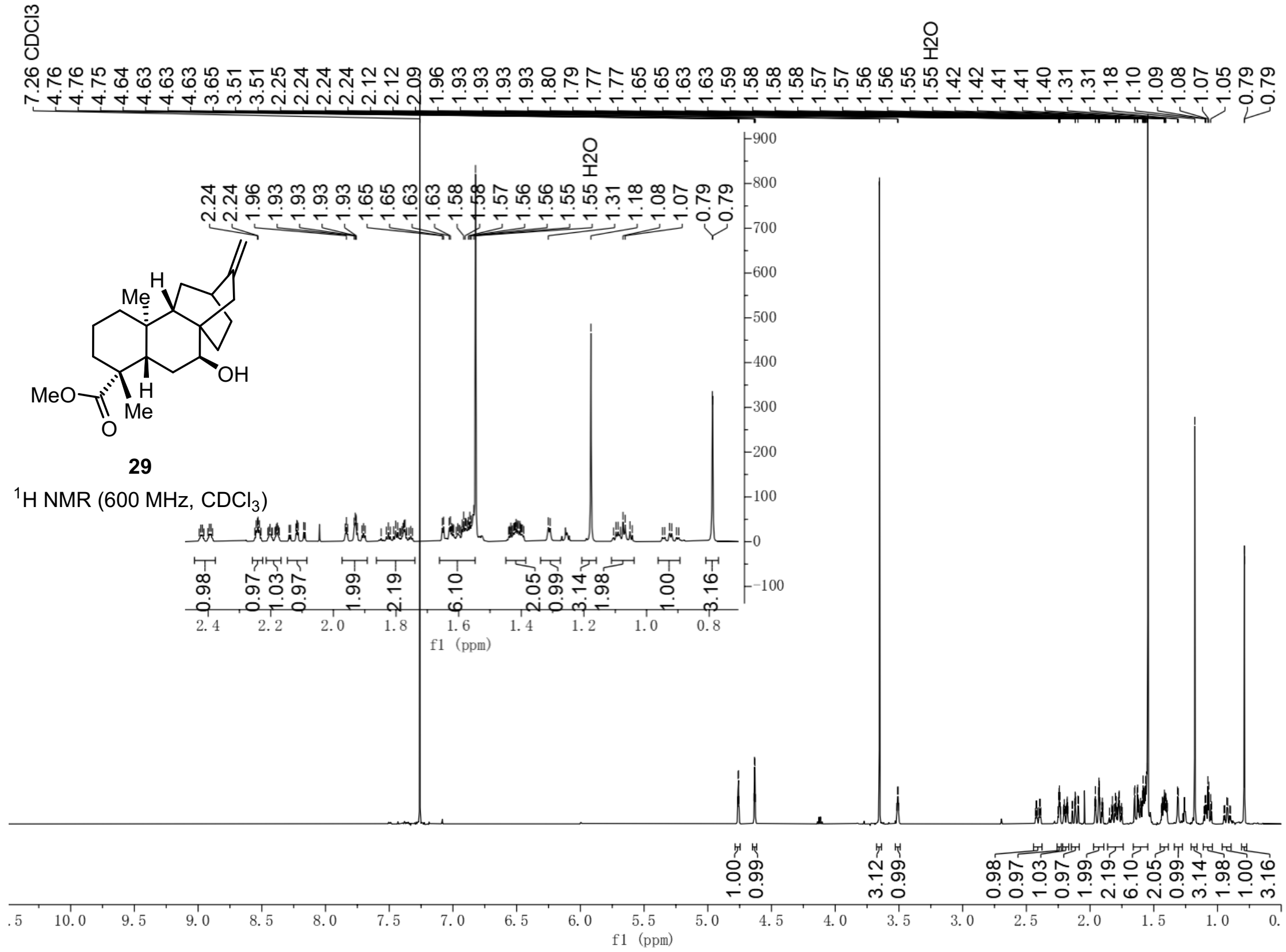

29

^{1}H NMR (600 MHz, $CDCl_3$)

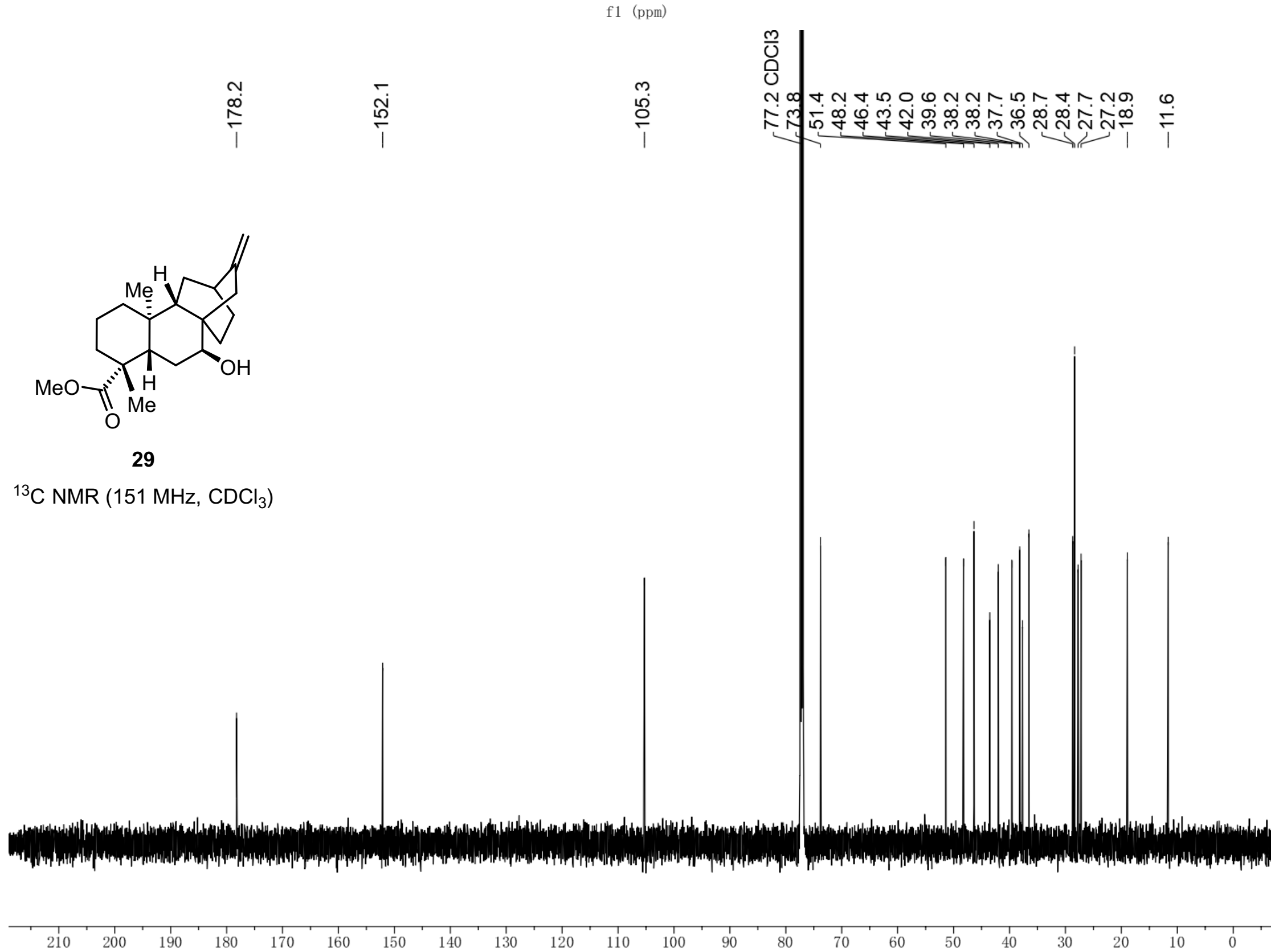

29

^{13}C NMR (151 MHz, $CDCl_3$)

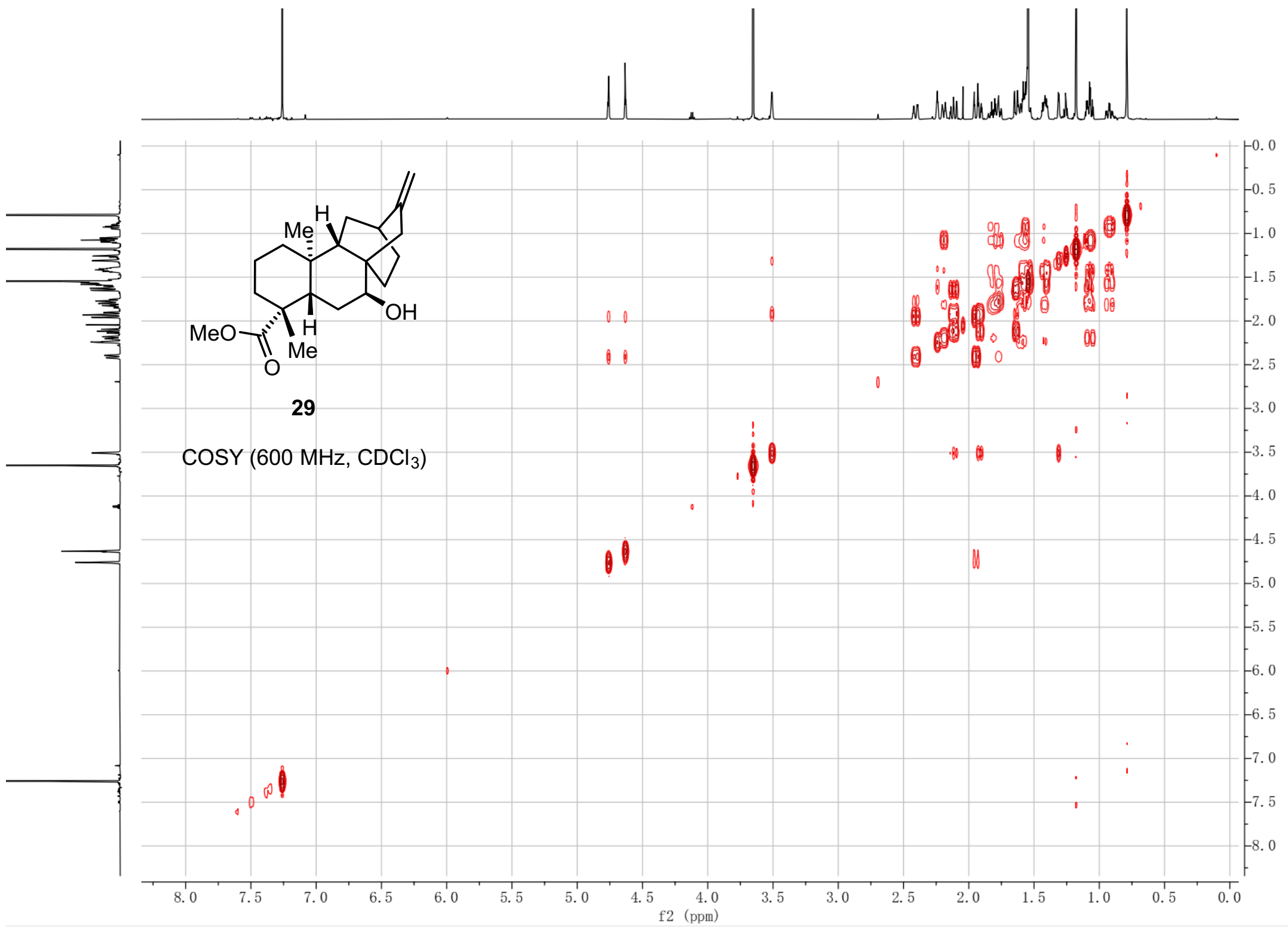
Me
H
MeO
OH
H
Me
O
29
COSY (600 MHz, CDCl3)
f2 (ppm)

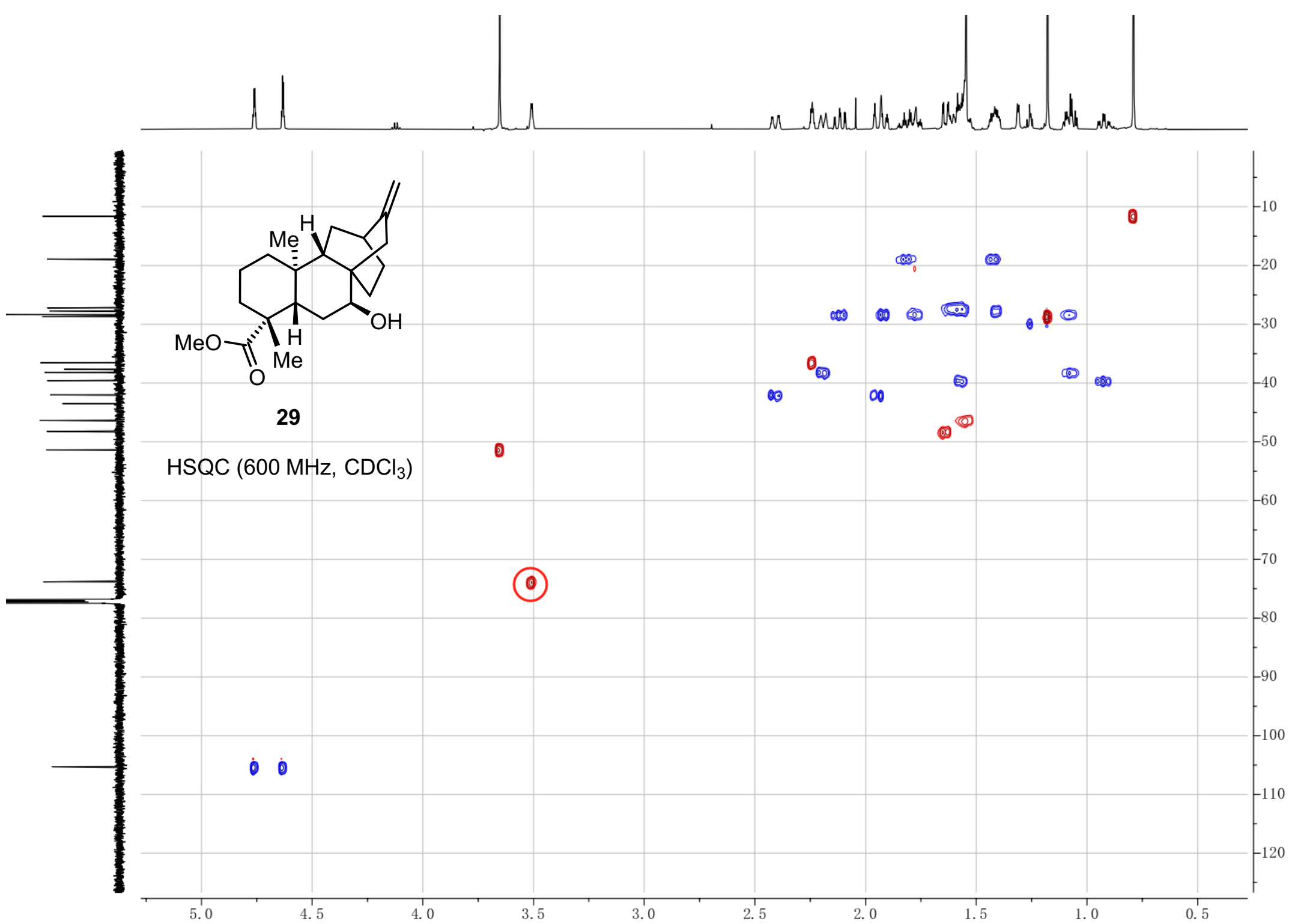
H
Me
MeO
OH
H
Me
O
29
HSQC (600 MHz, CDCl3)
f2 (ppm)

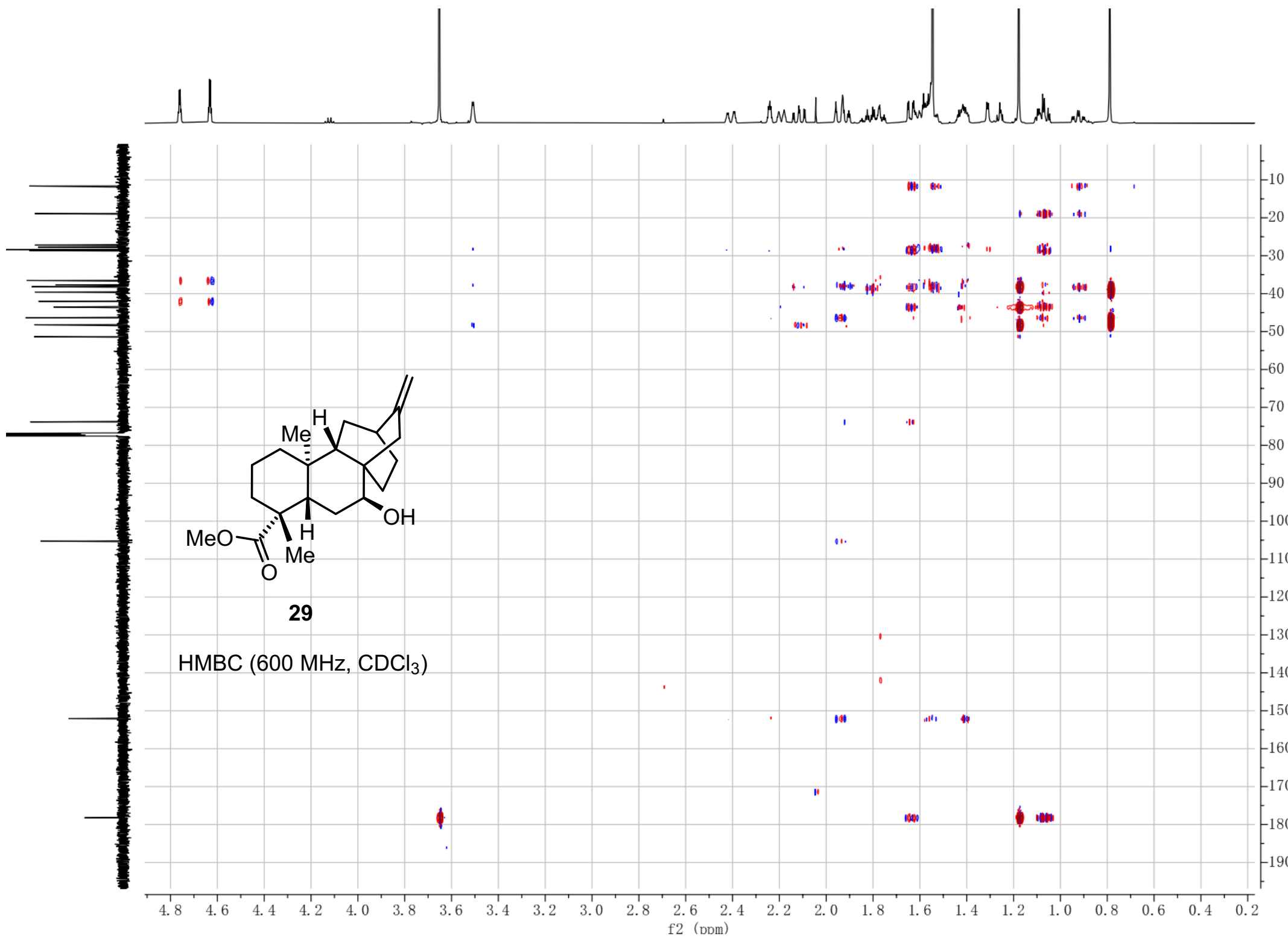
Me
H
MeO
H
OH
Me
O
29
HMBC (600 MHz, CDCl3)
f2 (ppm)

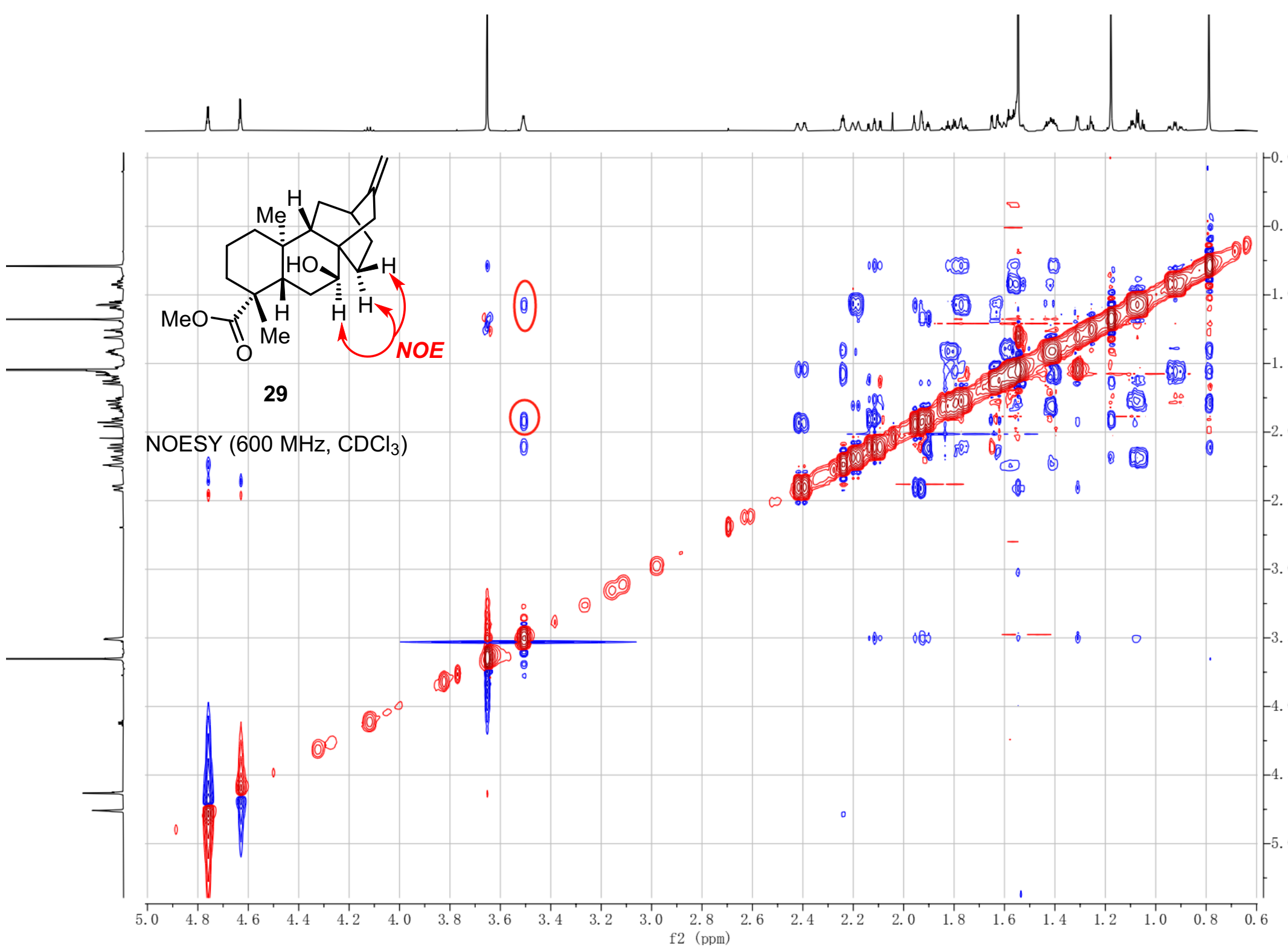
H
Me
HO
MeO
H
H
H
H
Me
O
NOE
29
NOESY (600 MHz, CDCl3)
f2 (ppm)

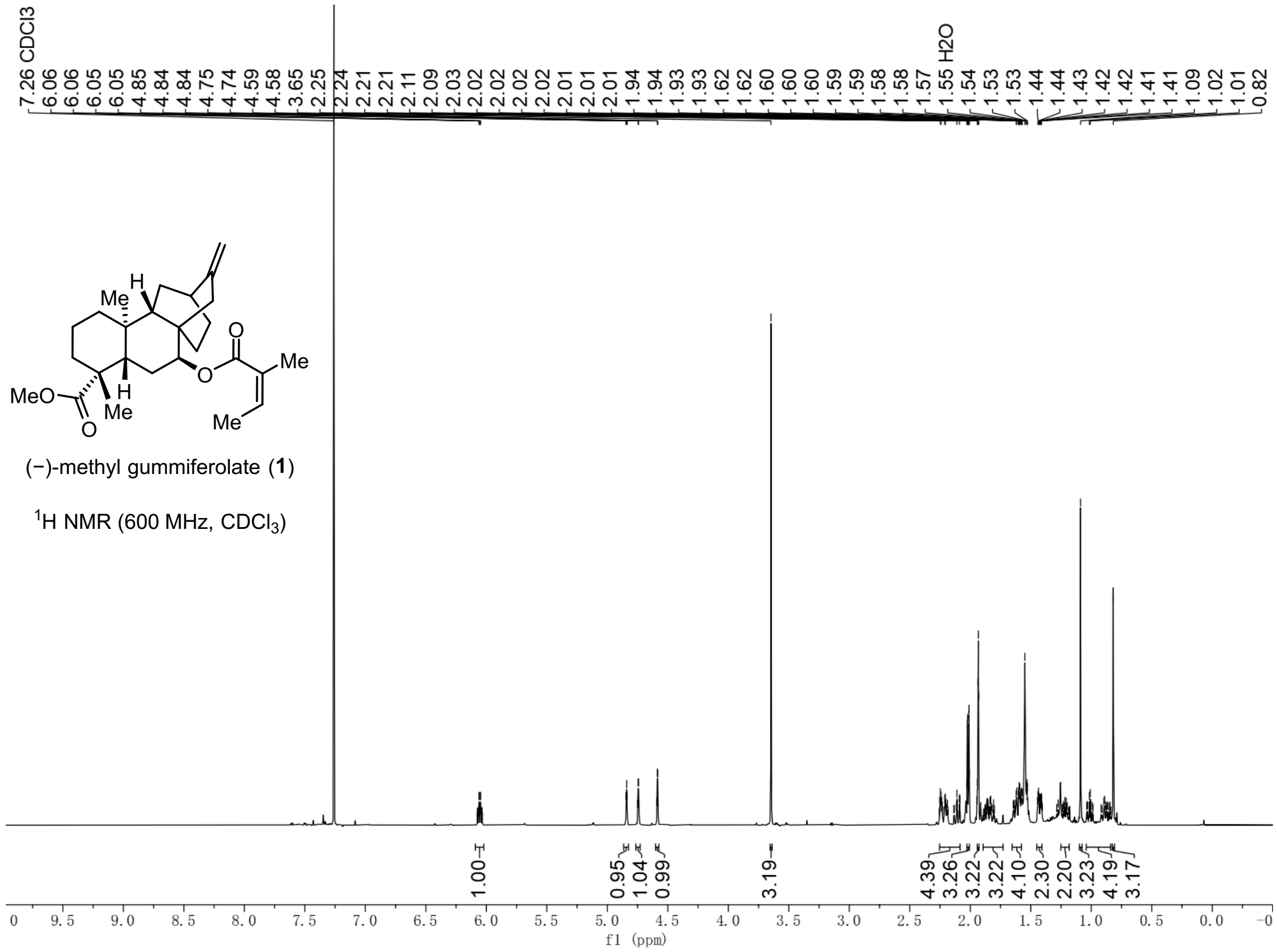
7.26 CDCl3
6.06 6.06 6.05 6.05 4.85 4.84 4.84 4.75 4.74 4.59 4.58 3.65 2.25 2.24 2.21 2.21 2.11 2.09 2.03 2.02 2.02 2.02 2.02 2.01 2.01 2.01 1.94 1.94 1.93 1.93 1.62 1.60 1.60 1.60 1.59 1.59 1.58 1.58 1.57 1.55 H2O 1.54 1.53 1.53 1.44 1.44 1.43 1.42 1.41 1.41 1.09 1.02 1.01 0.82
Me
H
MeO
O
H
Me
O
O
Me
Me
(−)-methyl gummiferolate (1)
1H NMR (600 MHz, CDCl3)
1.00
0.95 1.04 0.99
3.19
4.39 3.26 3.22 3.22 4.10 2.30 2.20 3.23 4.19 3.17
f1 (ppm)

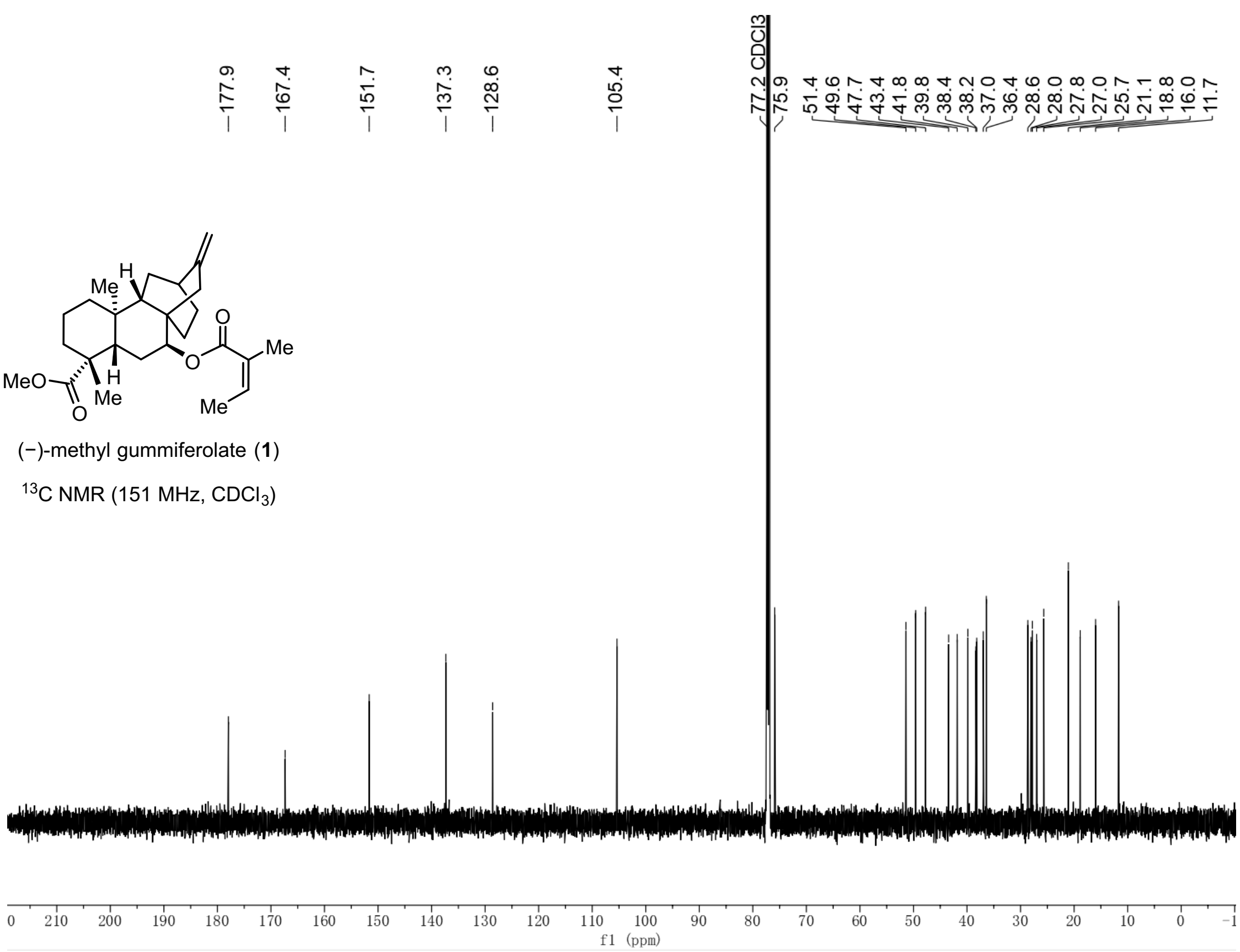
177.9
167.4
151.7
137.3
128.6
105.4
77.2 CDCl3
75.9
51.4 49.6 47.7 43.4 41.8 39.8 38.4 38.2 37.0 36.4 28.6 28.0 27.8 27.0 25.7 21.1 18.8 16.0 11.7
Me
H
MeO
O
H
Me
O
O
Me
Me
(−)-methyl gummiferolate (1)
13C NMR (151 MHz, CDCl3)
f1 (ppm)
